# Functional Imaging of CYP3A4 at Multiple Dimensions Using an AI‐Driven High Performance Fluorogenic Substrate

**DOI:** 10.1002/smll.202412178

**Published:** 2025-03-21

**Authors:** Feng Zhang, Lilin Song, Ruixuan Wang, Bei Zhao, Jian Huang, Luling Wu, Yufan Fan, Hong Lin, Zhengtao Jiang, Xiaodi Yang, Hairong Zeng, Xin Yang, Tony D. James, Guangbo Ge

**Affiliations:** ^1^ State Key Laboratory of Discovery and Utilization of Functional Components in Traditional Chinese Medicine Shanghai Frontiers Science Center of TCM Chemical Biology Institute of Interdisciplinary Integrative Medicine Research Shanghai University of Traditional Chinese Medicine Shanghai 201203 China; ^2^ Liaoning Provincial Key Laboratory of Carbohydrates Dalian Institute of Chemical Physics Chinese Academy of Sciences Dalian 116023 China; ^3^ Pharmacology and Toxicology Division Shanghai Institute of Food and Drug Control Shanghai 201203 China; ^4^ Department of Chemistry University of Bath Bath BA2 7AY UK; ^5^ Innovation Research Institute of Traditional Chinese Medicine Shanghai University of Traditional Chinese Medicine Shanghai 201203 China; ^6^ Department of Electrical and Electronic Engineering School of Engineering Cardiff University Cardiff CF24 3AA UK; ^7^ School of Chemistry and Chemical Engineering Henan Normal University Xinxiang 453007 China

**Keywords:** artificial intelligent (AI)‐powered molecular design, cytochrome P450 3A4 (CYP3A4), fluorogenic substrate, functional imaging, inhibitor screening

## Abstract

Cytochrome P450 3A4 (CYP3A4) is a key mediator in xenobiotic metabolism and drug‐drug interactions (DDI), developing orally active fluorogenic substrates for sensing and imaging of a target enzyme in biological systems remains challenging. Here, an artificial intelligence (AI)‐driven strategy is used to construct a highly specific and orally active fluorogenic substrate for imaging CYP3A4 in complex biological systems. After the fusion of an AI‐selected drug‐like fragment with a CYP3A4‐preferred fluorophore, three candidates are designed and synthesized. Among all evaluated candidates, NFa exhibits excellent isoform‐specificity, ultra‐high sensitivity, outstanding spatial resolution, favorable safety profiles, and acceptable oral bioavailability. Specifically, NFa excels at functional in situ imaging of CYP3A4 in living systems with exceptional endoplasmic reticulum (ER)‐colocalization performance and high imaging resolution, while this agent can also replace hCYP3A4 drug‐substrates for high‐throughput screening of CYP3A4 inhibitors and for assessing DDI potential in vivo. With the help of NFa, a novel CYP3A4 inhibitor (D13) was discovered, and its anti‐CYP3A4 effects are assessed in live cells, ex vivo and in vivo. Collectively, an AI‐powered strategy is adapted for developing highly‐specific and drug‐like fluorogenic substrates, resulting in the first orally available tool (NFa) for sensing and imaging CYP3A4 activities, which facilitates CYP3A4‐associated fundamental investigations and the drug discovery process.

## Graphical abstract

This study showcases a novel artificial intelligence (AI)‐driven design strategy for constructing highly specific and orally‐active fluorogenic substrates for imaging CYP3A4 in complex biological systems, while **NFa** emerged as a practical and reliable tool for sensing and imaging of CYP3A4 activity thus facilitating CYP3A4‐associated fundamental investigations and the drug discovery process.

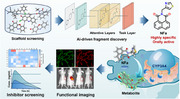

## 1 Introduction

Cytochrome P450 enzymes (CYPs), a class of heme‐containing monooxygenases, play a crucial role in oxidative metabolism and biotransformation in a variety of organisms.^[^ ^1^, ^2^, ^3^ ^]^ To date, a total of 57 functional CYPs have been identified in humans, which are classified into 18 families and 44 subfamilies based on sequence similarity.^[^ ^4^, ^5^ ^]^ Among all identified human CYPs, cytochrome P450 3A4 (CYP3A4) has stimulated great interest, owing to its high abundance in human metabolic organs (such as the liver and the small intestine), as well as its very broad substrate spectrum and biologically diverse functions.^[^ ^6^, ^7^, ^8^, ^9^ ^]^ CYP3A4 catalyzes the oxidative metabolism of numerous xenobiotics (including ≈50% of marketed drugs) and a wide range of hydrophobic endogenous substances (such as steroids). As a result, CYP3A4 modulators may strongly affect drug treatment outcomes and metabolic homeostasis.^[^ ^8^, ^10^, ^11^, ^12^ ^]^ Currently, several marketed CYP3A4 inhibitors (such as ritonavir) have been used to prolong the metabolic half‐lives of CYP3A4‐substrate drugs (such as the combination drug Kaletra and Paxlovid) for achieving more efficacious therapeutic effects.^[^ ^13^, ^14^, ^15^, ^16^ ^]^ As an over‐expressed enzyme in breast cancer cells and some cancerous tissues, CYP3A4 is also recognized as a key protein to trigger multi‐drug resistance (MDR) via inactivating some important anti‐cancer drugs (such as paclitaxel, docetaxel, and vincristine).^[^ ^17^, ^18^, ^19^, ^20^ ^]^ By contrast, CYP3A4 inhibitors or inactivators can potentiate those CYP3A4‐substrate drugs and overcome MDR via inhibiting intracellular CYP3A4.^[^ ^21^, ^22^, ^23^ ^]^

Deciphering the relevance of CYP3A4 to human diseases and discovering CYP3A4 modulators requires practical and reliable tools for sensing CYP3A4 activity in living systems. Over the past few decades, drug substrates and physiological substrates for CYP3A4 (such as testosterone, bufalin, and midazolam) are frequently used for detecting the activity levels of CYP3A4.^[^ ^24^, ^25^, ^26^, ^27^, ^28^ ^]^ Notably, liquid chromatography‐mass spectrometry based assays are routinely used for quantifying the drug/physiological substrates and their oxidative metabolites, which requires time‐consuming sample preparation procedures, high‐cost devices, and professional operators.^[^ ^29^, ^30^ ^]^ By contrast, fluorogenic substrates take advantage of ultrahigh sensitivity, rapid response, are easy‐to‐use, capable of high‐throughput evaluation and in situ imaging, therefore providing powerful tools for in situ sensing and imaging of enzyme function and for highly efficient screening of enzyme modulators.^[^ ^31^, ^32^, ^33^, ^34^ ^]^ Although several fluorogenic substrates for hCYP3A4 have been developed in recent years (Table S1, Supporting Information),^[^ ^35^, ^36^, ^37^, ^38^, ^39^, ^40^ ^]^ none of these agents could be used to replace the drug/physiological substrates for ex vivo and in vivo tests. Specifically, almost all reported fluorogenic substrates show very poor cell‐membrane permeability and extremely low oral bioavailability, which significantly limits their use in live cells, ex vivo and in vivo.^[^ ^35^, ^36^ ^]^ Notably, more than half of the CYP3A4‐substrate drugs are orally available agents, blocking intestinal hCYP3A4 may also cause clinically significant DDI.^[^ ^41^, ^42^ ^]^ Addressing these issues requires the development of more practical, and orally available visualization tools to replace CYP3A4‐drug substrates for in situ monitoring of the dynamic changes in CYP3A4 activities in complex biological systems.

Ideal orally available CYP3A4 fluorogenic substrates should meet the following requirements: 1) high specificity, 2) high turnover rate, 3) good photostability, 4) favorable safety profiles, 5) high cell‐membrane permeability enabling the substrates to cross the intestinal barrier into the circulating system. Unfortunately, almost all fluorophores have planar, rigid polyaromatic scaffolds, which may act as preferred substrates for CYP enzymes but are difficult to cross the intestinal barrier or cell membrane.^[^ ^43^, ^44^, ^45^, ^46^ ^]^ In fact, all previously reported CYP3A4 fluorogenic substrates possess planar and rigid scaffolds that show very poor cell‐membrane permeability or extremely low oral availability, which significantly blocks their ex vivo and in vivo applications (Table S1, Supporting Information). Making it an urgent requirement to develop more effective strategies for practical and orally available CYP3A4 fluorogenic substrates with improved cell‐membrane permeability and desirable pharmacokinetic properties.

**Scheme 1:**
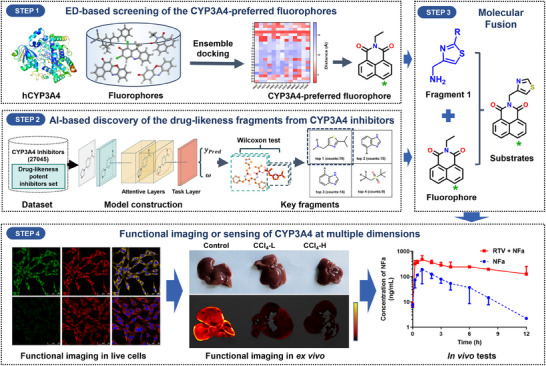
An intelligent structural design strategy for the design and development of CYP3A4 fluorogenic substrates.

Here, an intelligent strategy (**Scheme** **1**) was adopted to rationally construct highly specific and orally active fluorogenic substrates for CYP3A4 by integrating artificial intelligence (AI) with ensemble docking (ED). In brief, an AI‐driven approach was used to select drug‐like fragments from potent and orally active CYP3A4 inhibitors, which were then fused with a suitable fluorophore (selected by ED‐based screening) to generate CYP3A4 fluorogenic substrate candidates. Of which the preferred metabolic site for CYP3A4 was conserved, while the AI‐selected drug‐like fragment was used to improve the cell‐membrane permeability and oral bioavailability without affecting the selectivity toward CYP3A4. Using this strategy, several AI‐driven chemically synthesizable CYP3A4 fluorogenic substrates were developed, in which **NFa** was discovered as a practical, rapidly responding, and highly specific fluorogenic substrate for CYP3A4. These findings encouraged us to further investigate the performance of **NFa** for functional imaging of hCYP3A4 in various biological systems in multiple dimensions, as well as the applicability of **NFa** for high‐throughput inhibitor screening and precisely assessing DDI potentials in live cells, ex vivo and in vivo.

## 2 Results and Discussion

### 2.1 Virtual Screening of the Most Suitable Fluorophores as hCYP3A4 Substrates

Since hCYP3A4 prefers to catalyze the hydroxylation of hydrophobic polyaromatic compounds to form the corresponding mono‐hydroxylated metabolites, 22 chemically diverse fluorophores with polyaromatic scaffolds without any hydrophilic group were collected to determine suitable fluorophores as hCYP3A4 substrates. First, each fluorophore was docked with 13 previously reported hCYP3A4 crystal structures (PDB Bank, 3NXU, 3UA1, 4D75, 4I4G, 4K9U, 5TE8, 5VC0, 5VCG, 6BD6, 6MA6, 6MA7, 6UNJ, 7KVN) by ensemble docking (ED). The distances between the hydroxylated site of the first conformation of each fluorophore (indicated by the red arrow) and the Fe atom in the heme of hCYP3A4 were then measured. Among all evaluated fluorophores, fluorophore 4 (*N*‐ethyl‐1,8‐naphthalimide) exhibited the highest potential as a hCYP3A4 substrate, where the distances between the hydroxylated site of this fluorophore (C‐4 site) and the Fe atom in all CYP3A4 crystal structures were less than 4.0 Å (Figure S1, Supporting Information). These findings suggested that *N*‐ethyl‐1,8‐naphthalimide was the most suitable fluorophore as a hCYP3A4 substrate candidate. However, *N*‐ethyl‐1,8‐naphthalimide exhibited very poor cell‐membrane permeability, and this agent could not be easily absorbed into the circulating system when taken orally,^[^ ^47^, ^48^ ^]^ which prompted us to further modify this fluorophore to generate an orally available CYP3A4 fluorogenic substrate.

### 2.2 Searching the Drug‐Like Fragments of Orally Active CYP3A4 Inhibitors

**Figure 1:**
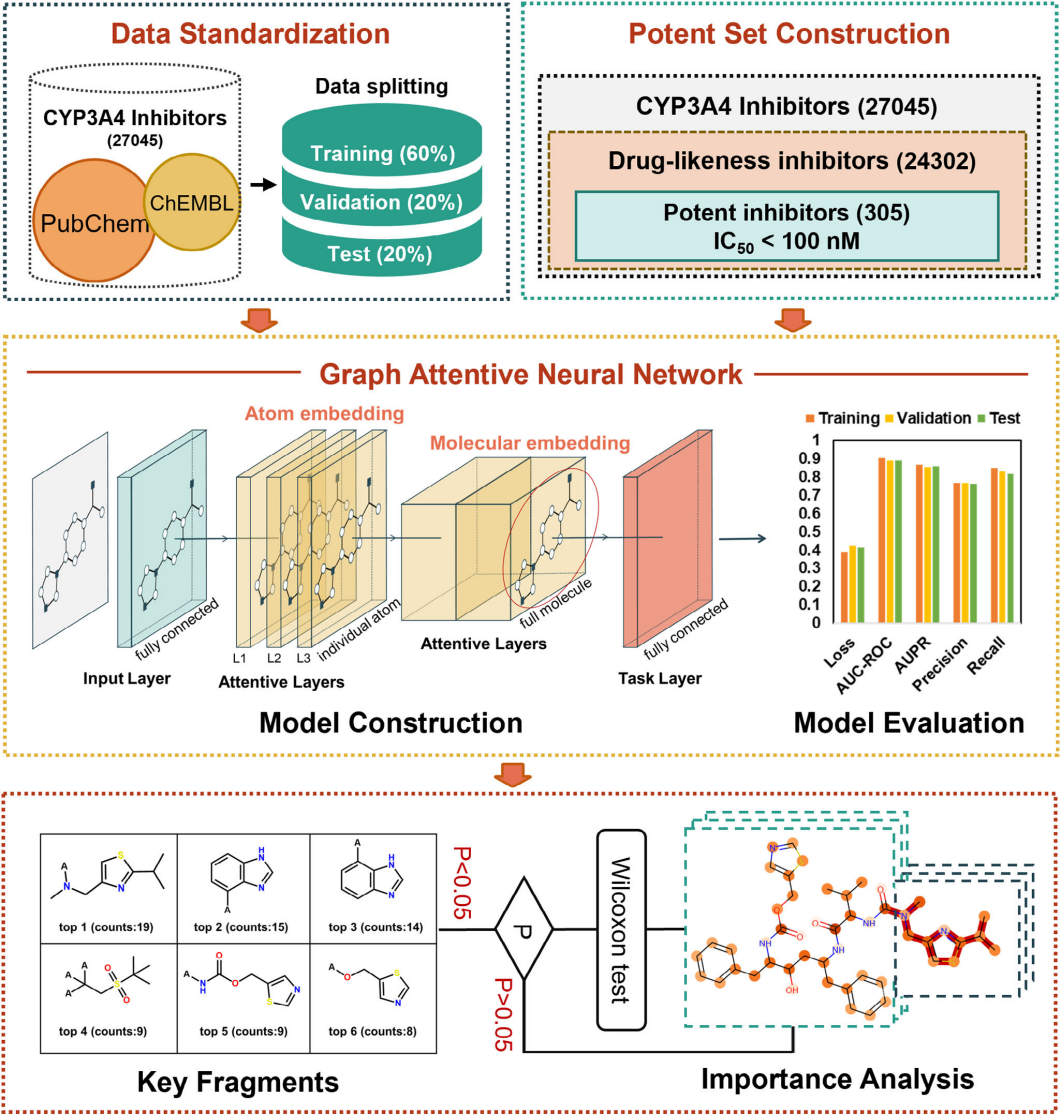
Searching for the drug‐likeness fragments from CYP3A4 inhibitors using artificial intelligence technique.

Since the north part of 1,8‐naphthalimides could be easily modified, while the north part was far away from the metabolic site (C4 site) and such modification does not affect the fluorescent properties of the 1,8‐naphthalimide derivatives, it is feasible to improve the cell‐membrane permeability and oral availability of 1,8‐naphthalimides via introducing a drug‐like fragment derived from orally active CYP3A4 ligands. To this end, a comprehensive CYP3A4 inhibitor dataset was constructed by integrating all previously reported CYP3A4 inhibitor datasets (Table S2, Supporting Information), while a classification model (AFP‐3A4) was constructed for predicting the importance of each atom/fragment for CYP3A4 inhibition (**Figure** **1**; Figure S2 and Table S3, Supporting Information). To seek a suitable drug‐like fragment with both high binding affinity toward CYP3A4 and acceptable oral availability, all assembled CYP3A4 inhibitors were preliminarily screened using the following drug‐like criteria (250 < MW < 750, number of H acceptors <10, number of H donors < 5, −2 < cLogP < 7, and TPSA < 150).^[^ ^49^, ^50^ ^]^ A total of 24 302 CYP3A4 inhibitors were found with acceptable oral availability, while part of them (305 compounds) exhibited high anti‐CYP3A4 effect (IC_50_ < 100 nm). These agents were then extracted from ChEMBL for differential fragment analysis to understand the importance of each atom for CYP3A4 inhibition using the well‐tuned AFP‐3A4 model. A total of 148 key fragments with significant differences (*P* < 0.05) were obtained and then ranked based on their frequency of occurrence as potent CYP3A4 inhibitors, then the top 6 fragments were selected (as depicted in Figure S3, Supporting Information). Among these drug‐like fragments, 2‐isopropylthiazol‐4‐yl‐methanamine exhibited the highest frequency of occurrence (19 times), which was much higher than the second‐ranked fragment (appearing 15 times). These results encouraged us to fuse the top 1 drug‐like fragment with 1,8‐naphthalimide to develop highly specific CYP3A4 fluorogenic substrates with improved drug‐like properties.

### 2.3 Rational Design of Specific Substrates for hCYP3A4

**Figure 2:**
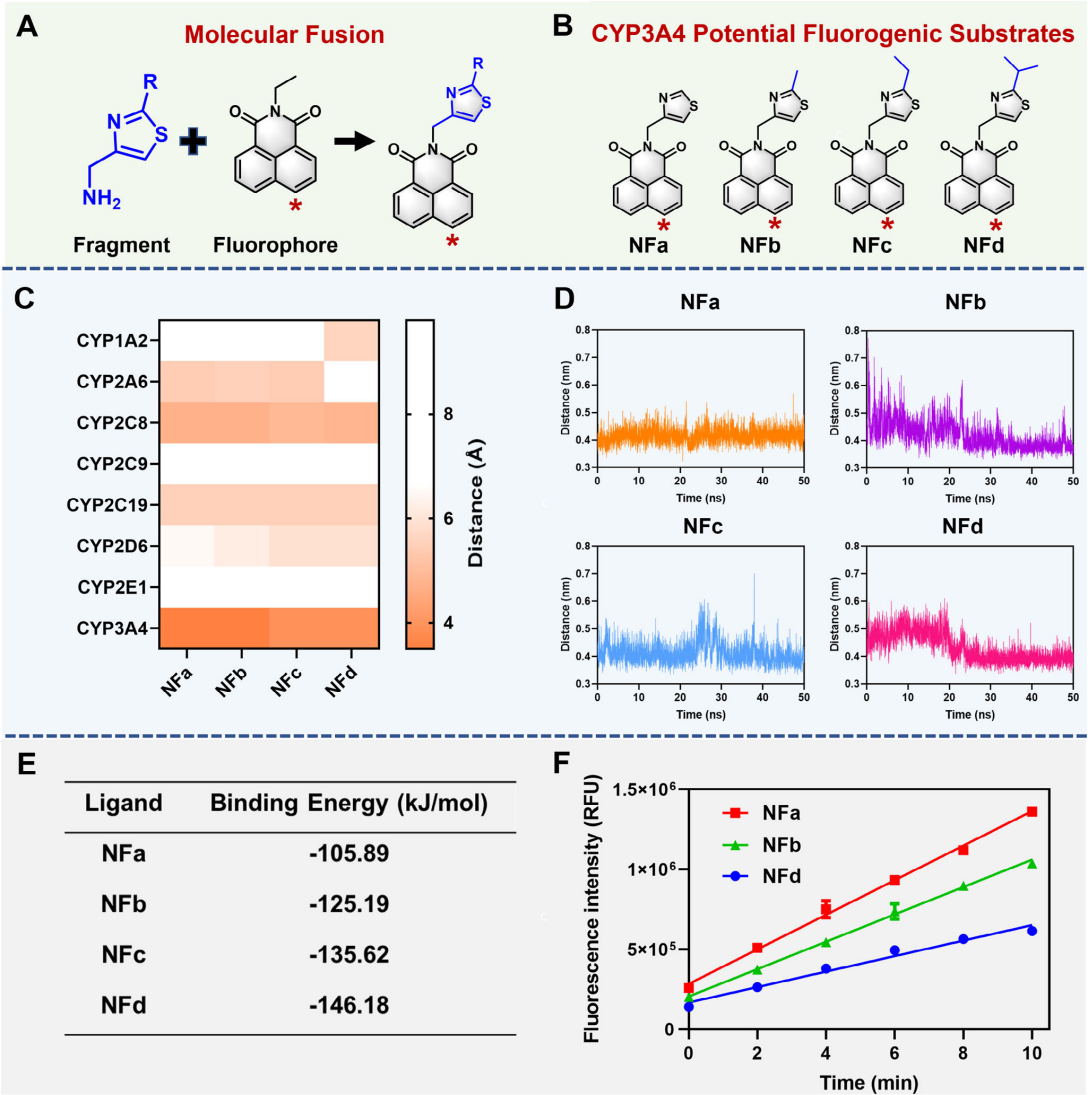
Structural splicing and property optimization of CYP3A4 drug‐likeness fragment and suitable fluorophore. A) Design of novel fluorescent substrates via molecular fusion. B) Chemical structures of four potential CYP3A4 fluorescent substrates. C) Ensemble docking of the CYP3A4 fluorogenic substrate candidates into eight major hepatic CYPs. D, E) Molecular dynamics (D, distance; E, binding energy) of CYP3A4 potential fluorescent substrates into hCYP3A4. F) Reaction rates of **NFa**, **NFb**, **NFd** in hCYP3A4. Data are expressed as mean ± SD (n = 3).

The selected drug‐like fragment and its analogs were then used to design novel CYP3A4 fluorogenic substrate candidates by fusing these drug‐like fragments on the north part of *N*‐ethyl‐1,8‐naphthalimide. As shown in **Figure** **2**A,B, four CYP3A4 fluorogenic substrate candidates (**NFa**, **NFb**, **NFc**, and **NFd**) were designed and these molecules were then docked with eight important hepatic P450 enzymes (CYP1A2, CYP2A6, CYP2C8, CYP2C9, CYP2C19, CYP2D6, CYP2E1, CYP3A4). It was found that the distances between these newly designed CYP3A4 fluorogenic substrate candidates and the heme iron of CYP3A4 were all within 4.00 Å, while the distances to the heme iron for other P450 isoenzymes were all above 4.50 Å (Figure 2C). These observations suggested that these new substrate candidates might act as highly specific CYP3A4 fluorogenic substrates. Further molecular dynamic simulations showed that these new substrate candidates could tightly bind to the cavity of CYP3A4 and form stable catalytic conformations, especially **NFa** (Figure 2D,E). These observations indicate that these newly designed 1,8‐naphthalimide derivatives are more likely to act as specific fluorogenic substrates for CYP3A4, prompting us to synthesize and characterize these derivatives.

### 2.4 NFs can be Specifically Activated by hCYP3A4

Next, **NFa**, **NFb**, **NFd**, and their mono‐hydroxylated metabolites (**4‐HNFa**, **4‐HNFb**, **4‐HNFd**) at the C‐4 site were synthesized and characterized (Scheme S1, Supporting Information, **NFc**, and **4‐HNFc** were not prepared, owing to the lack of suitable starting materials). As shown in Figures S4 and S5 (Supporting Information), **NFa**, **NFb**, and **NFd** could be readily metabolized to form the corresponding mono‐hydroxylated metabolites of hCYP3A4 or human liver microsomes (HLMs), which were identified as **4‐HNFa**, **4‐HNFb**, **4‐HNFd**, respectively, by comparing their retention times and MS/MS fragments with appropriate standards. P450 reaction phenotyping assays indicated that **NFa**, **NFb**, and **NFd** were highly specific substrates for hCYP3A4 (Figures S6 and S7A–C, Supporting Information). Meanwhile, chemical inhibition assays showed that 1‐aminobenzotriazole (ABT, a broad‐spectrum CYP inhibitor), ketoconazole (KET, a potent inhibitor of mammalian CYP3A), or CYP3cide (an isoform‐specific inhibitor of CYP3A4), could completely block the **NFs** 4‐hydroxylation in HLMs (Figure S7D–F, Supporting Information). These observations clearly confirm that **NFa**, **NFb**, and **NFd** are highly specific fluorogenic substrates for CYP3A4 and that our intelligent structural design strategy is well‐suited for developing highly specific fluorogenic substrates for target enzyme(s).

### 2.5 NFa is the Optimized Fluorogenic Substrate for CYP3A4

The enzymatic kinetics of **NFa**, **NFb**, and **NFd** with hCYP3A4 were then characterized. As shown in Figures S7G–I and S8 (Supporting Information), **Table** **1** and Table S4 (Supporting Information), **NFs** 4‐hydroxylation by hCYP3A4 displayed Michaelis–Menten kinetics, with calculated *K*_m_ values of 13.33 ± 1.01 µm (**NFa**), 7.51 ± 0.49 µm (**NFb**) and 4.17 ± 0.21 µm (**NFd**). Meanwhile, the catalytic velocity (*V*_max_) of **NFa**, **NFb**, and **NFd** 4‐hydroxylation by hCYP3A4 were also calculated, exhibiting *V*_max_ values of 11.42 ± 0.34 nmol min^−1^ nmol^−1^ hCYP3A4 (**NFa**), 4.19 ± 0.09 nmol min^−1^ nmol^−1^ hCYP3A4 (**NFb**) and 2.10 ± 0.04 nmol min^−1^ nmol^−1^ hCYP3A4 (**NFd**), respectively. These results clearly indicate that introducing a methyl or isopropyl group to the drug‐like fragment could slightly improve the binding affinity toward hCYP3A4 but significantly decrease the turnover rate with CYP3A4. Among the three newly developed CYP3A4 fluorogenic substrates, **NFa** displayed the highest metabolic clearance (857 µL min^−1^ nmol^−1^ hCYP3A4) with hCYP3A4, encouraging us to use this agent to replace CYP3A4‐substrate drugs for further investigation.

**Table 1:** Kinetic parameters of 4‐hydroxylation of **NFa**, **NFb**, and **NFd** by hCYP3A4. Data are expressed as mean ± SD (n = 3).

Substrate	Metabolite	*V*_max_ ^a)^	*K*_m_ [µm]	*CL*_int_ ^b)^
**NFa**	**4‐HNFa**	11.42 ± 0.34	13.33 ± 1.01	857
**NFb**	**4‐HNFb**	4.19 ± 0.09	7.51 ± 0.49	558
**NFd**	**4‐HNFd**	2.10 ± 0.04	4.17 ± 0.21	504

^a)^ The unit of *V*_max_ is nmol/min/nmol hCYP3A4.

^b)^ The unit of *CL*_int_ *(V*_max_/*K*_m_) is µL min^−1^/nmol hCYP3A4.

### 2.6 Sensing Properties of NFa Toward hCYP3A4

**Figure 3:**
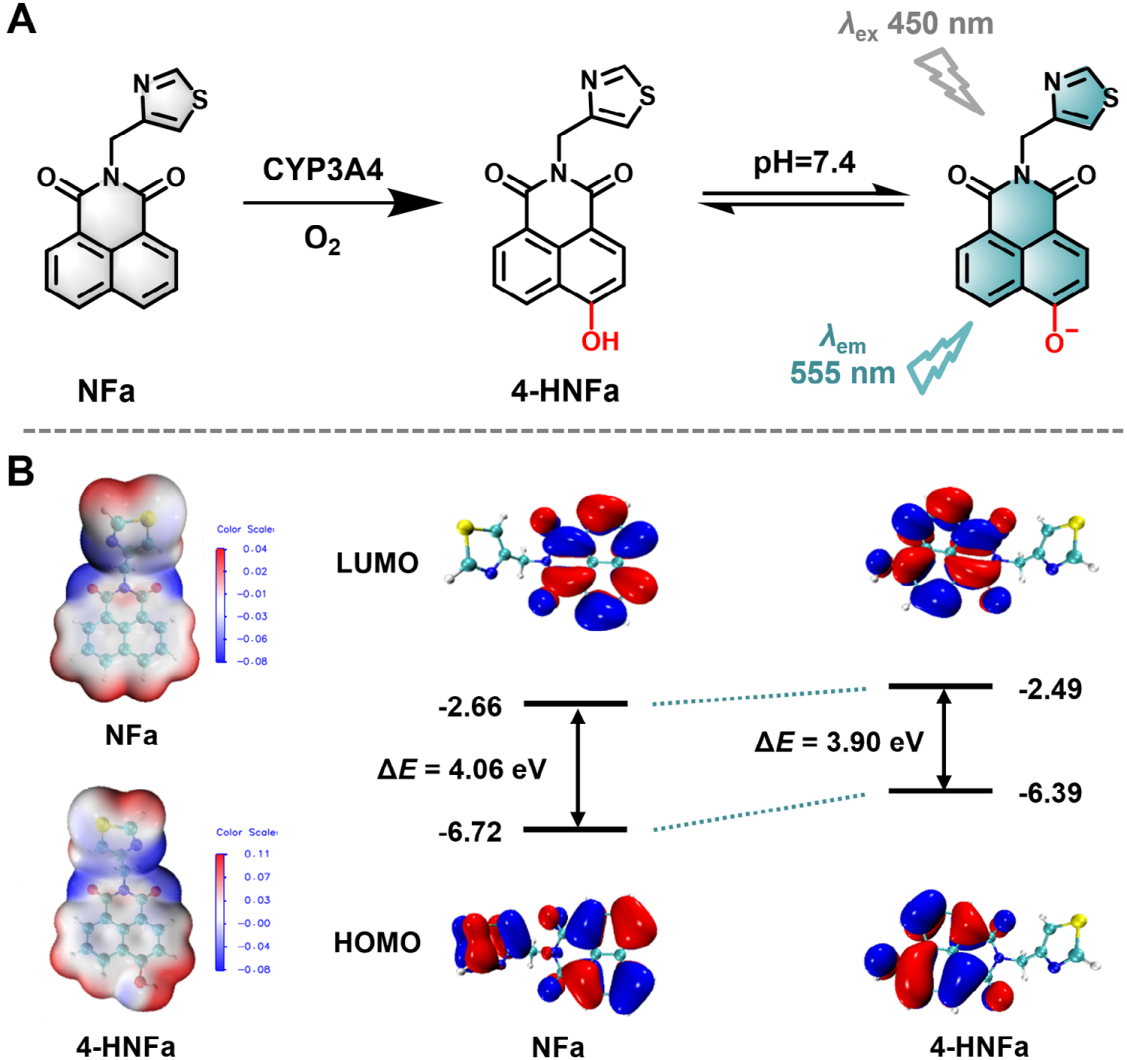
A) The sensing mechanism of **NFa** toward hCYP3A4. B) The electrostatic potential, frontier molecular orbitals, and energy levels of **NFa** and **4‐HNFa**.

**Figure 4:**
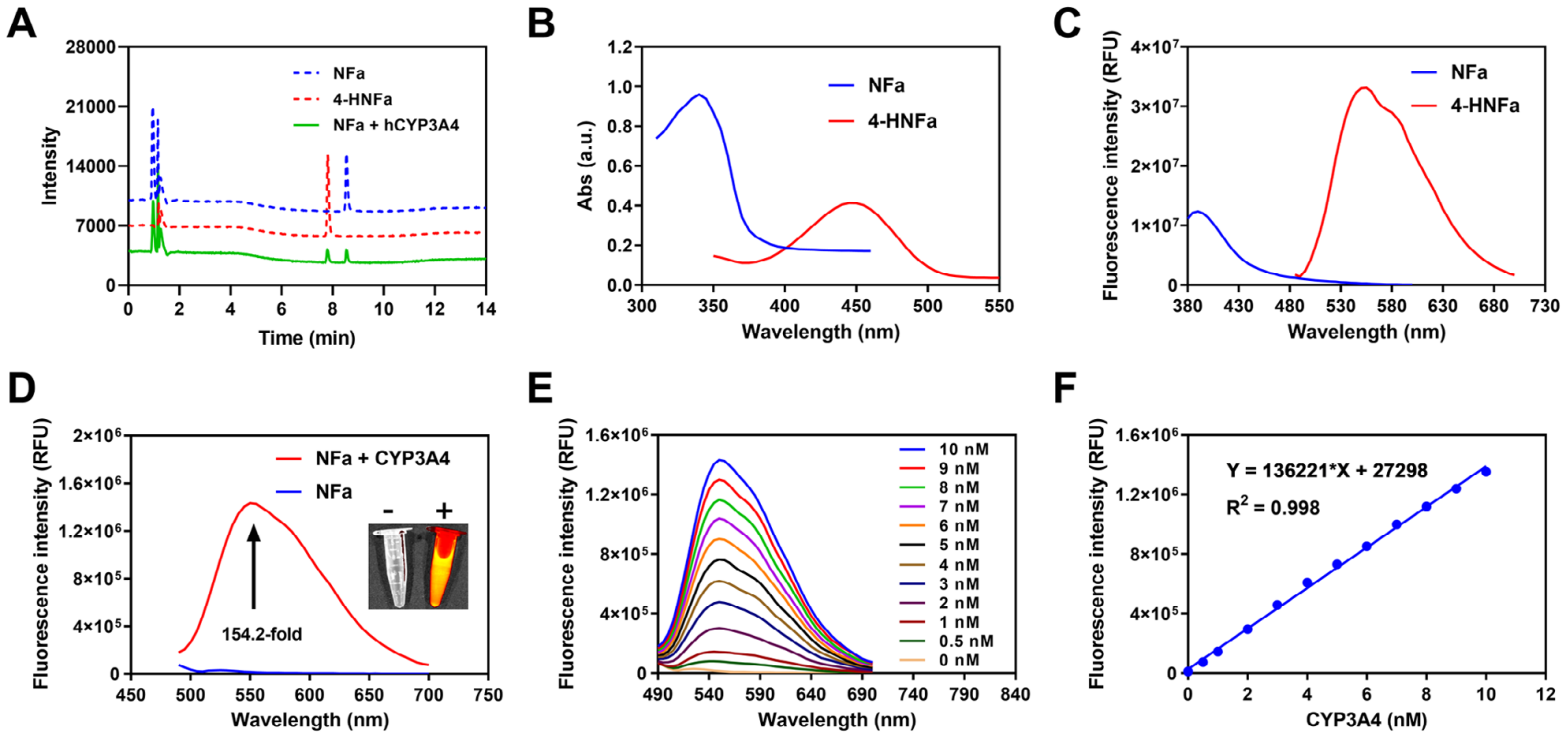
Optical and enzymatic behavior of **NFa** toward hCYP3A4. A) LC‐UV profiles of **NFa** and its hydroxylated metabolite (**4‐HNFa**). Top to bottom, **NFa** standard, **4‐HNFa** standard, **NFa** (10 µm) was incubated in HLMs (0.1 mg mL^−1^) for 30 min. The detection wavelength was set at 368 nm. B) The absorption spectrum of **NFa** and its oxidative metabolite **4‐HNFa**. C) Emission spectrum of **NFa** and **4‐HNFa**. D) The change in the emission spectrum of **NFa** (10 µm) in the presence of hCYP3A4 (10 nm) at 37 °C for 30 min, *λ*_ex_ = 450 nm. E) Fluorescence response of **NFa** (10 µm) toward increasing concentrations of hCYP3A4 (ranging from 0 to 10 nm) at 37 °C for 30 min. F) The linear relationship between the fluorescence intensity of **4‐HNFa** and the concentrations of hCYP3A4 (ranging from 0 to 10 nm). Data are expressed as mean ± SD (n = 3).

Next, the oxidative metabolite of **NFa** was biosynthesized and then purified by HPLC. The oxidative metabolite of **NFa** was fully characterized as **4‐HNFa** by both ^1^H‐NMR and ^13^C‐NMR, showing consistent spectra as the synthetic standard (Figures S9–S12, Supporting Information). These findings clearly suggested that **4‐HNFa** was the major metabolite of **NFa** in both mouse liver and human liver preparations (**Figure** **3**A). Meanwhile, density functional theory (DFT) calculations revealed that the HOMO‐LUMO gap of **NFa** was 4.06 eV, which was much higher than that of **4‐HNFa** (3.90 eV), suggesting that **NFa** acted as a typical turn‐on fluorescent substrate for CYP3A4 governed by the intramolecular charge transfer mechanism (Figure 3B). Next, the optical properties of both **NFa** and **4‐HNFa** were carefully evaluated (Tables S5 and S6, Supporting Information). As depicted in **Figure** **4**A–C, **NFa** exhibited a maximum UV absorption at 340 nm and maximum fluorogenic emission at 390 nm, while **4‐HNFa** displayed a maximum UV absorption at 450 nm and maximum fluorogenic emission at 555 nm. Following co‐incubation with hCYP3A4 for 0.5 h, a dramatic enhancement (154‐fold) in fluorescence emission ≈555 nm was observed (Figure 4D), while a good linear relationship (R^2^ = 0.998) between the fluorescence intensity and hCYP3A4 concentration (0–10 nm) was observed (Figure 4E,F). The limit of detection of **NFa** for sensing hCYP3A4 was also calculated as 0.047 nm, such sensitivity was much higher than all previously reported drug substrates and fluorogenic substrates for CYP3A4.^[^ ^34^, ^35^ ^]^ It was also found that flavin‐containing monooxygenase (FMOs) and aldehyde oxidase (AO) did not participate in the oxidative metabolism of **NFa** (Figure S13, Supporting Information). Moreover, the metabolic rate of **4‐HNFa** (t_1/2_ = 58.61 min) in HLMs in the presence of UDPGA is much lower than that of 7‐hydroxycoumarin (t_1/2_ = 16.89 min) (Figure S14, Supporting Information). Furthermore, **4‐HNFa** exhibited good photostability (Figure S15, Supporting Information) and emitted stable fluorescence signals ≈555 nm over a wide pH range (6.5–12.0) (Figure S16, Supporting Information). Additionally, **NFa** 4‐hydroxylation by hCYP3A4 exhibited no interference from commonly existing endogenous fatty acids, amino acids, and cations in biological samples (Figure S17, Supporting Information). These findings indicate that **NFa** can be specifically and rapidly metabolized by hCYP3A4 to form a brightly fluorogenic metabolite (**4‐HNFa**) with rapid response and ultra‐high sensitivity.

### 2.7 Functional Imaging of CYP3A4 in Living Cells

**Figure 5:**
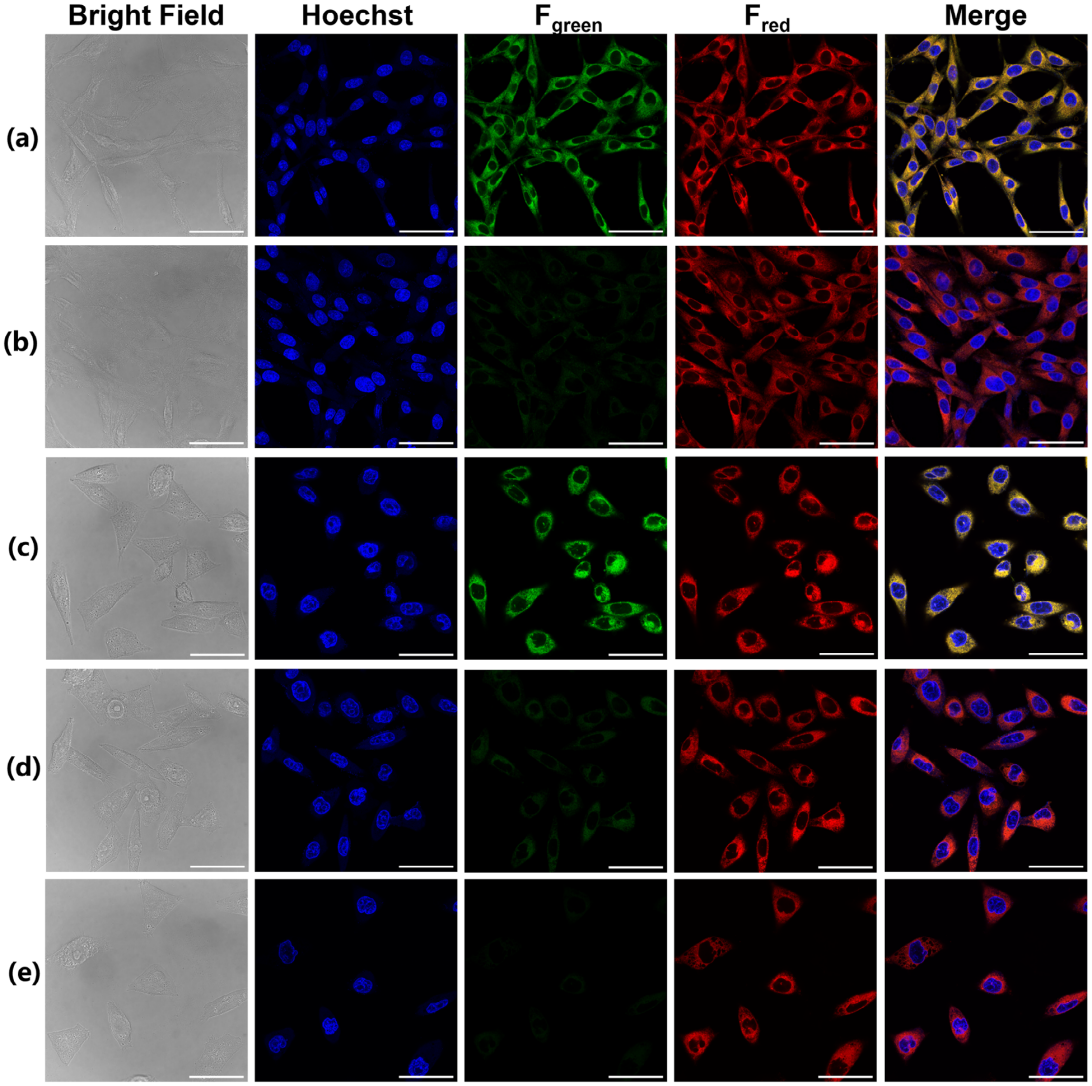
Functional imaging of hCYP3A4 in living Hep3B (a, b) and MCF‐7 (c‐e) cells using confocal laser scanning microscopy. The Hep3B (a) and MCF‐7 (c) cells were incubated with **NFa** (20 µm, green channel) for 1 h, Hoechst33342 (blue channel), and ER tracker (red channel) for 15 min. The Hep3B (b) and MCF‐7 (d) cells were treated with RTV (20 µm) for 1 h, following staining by **NFa** for 1 h and staining by Hoechst33342 and ER tracker for 15 min. The MCF‐7 (e) cells were treated with KET (20 µm) for 1 h, following staining by **NFa** for 1 h and staining by Hoechst33342 and ER tracker for 15 min. Scale bar: 50 µm.

**Figure 6:**
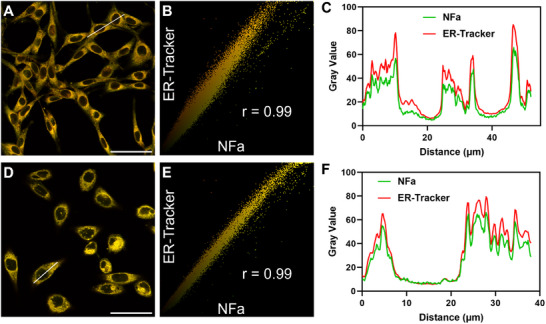
Co‐localization assay of **NFa** and ER Tracker Red in living Hep3B (A, B, C) and MCF‐7 (D, E, F) cells. A, D) Merged image of green channel for **NFa** and Red channel for ER‐Tracker. B, E) Intensity correlation scatter plot of **NFa** and ER‐Tracker Red. C, F) Intensity profiles of the linear region of interest (ROI) across the cell (white line). Scale bar: 50 µm.

Considering the excellent fluorescent substrate properties of **NFa**, which was then used for the functional imaging of CYP3A4 in living cells. Before imaging, the cell‐membrane permeability of **NFa** was evaluated under identical conditions. As shown in Figure S18 (Supporting Information), the cell‐membrane permeability of **NFa** in both NCM460 and Hep3B cells was much higher than that of **NEN**, with dramatic enhancements of 28‐fold and 5.6‐fold in NCM460 and Hep3B cells, respectively. These results suggest that **NFa** shows good cell‐membrane permeability. Furthermore, **NFa** is a non‐substrate for P‐gp, owing to the intracellular exposure levels of **NFa** cannot be elevated following the addition of a P‐gp inhibitor verapamil (Figure S19, Supporting Information). These findings encouraged us to use **NFa** for the functional imaging of CYP3A4 in tumors and in living systems. In addition, it was found that both **NFa** and **4‐HNFa** displayed weak cytotoxicity toward a panel of mammalian cells, including MCF‐7, Hep3B, and U87 cells (Figures S20–S22, Supporting Information). As shown in **Figure** **5** and Figures S23–S24 (Supporting Information), after co‐culturing with **NFa** for 1 h, bright fluorescence signals (green channel) were emitted from the endoplasmic reticulum (ER) of MCF‐7/Hep3B cells, with high spatial imaging resolution and high signal‐to‐noise ratio. Co‐localization assays of **NFa** and ER‐Tracker Red in MCF‐7 and Hep3B cells clearly showed that **NFa** possessed exceptional ER‐colocalization performance with a very high Pearson's coefficient of 0.99 (**Figure** **6**A,B,D,E). Meanwhile, the linear region of interest (ROI) intensity change was also synchronized for both channels (Figure 6C,F). Notably, the fluorescence signals (green channel) at the ER of MCF‐7/Hep3B cells could be blocked by the addition of CYP3A4 inhibitors (Ritonavir (RTV) or KET). In sharp contrast, no significant green fluorescent signals were observed in U87 cells, owing to the very low CYP3A4 expression level in these cells (Figures S23–S25, Supporting Information). These results clearly validate that **NFa** is a practical tool for the functional imaging of CYP3A4 in living cells, exhibiting exceptional ER‐colocalization performance and high spatial imaging resolution.

### 2.8 Visualization of CYP3A4 in Tissue Preparations and Organs Using NFa

**Figure 7:**
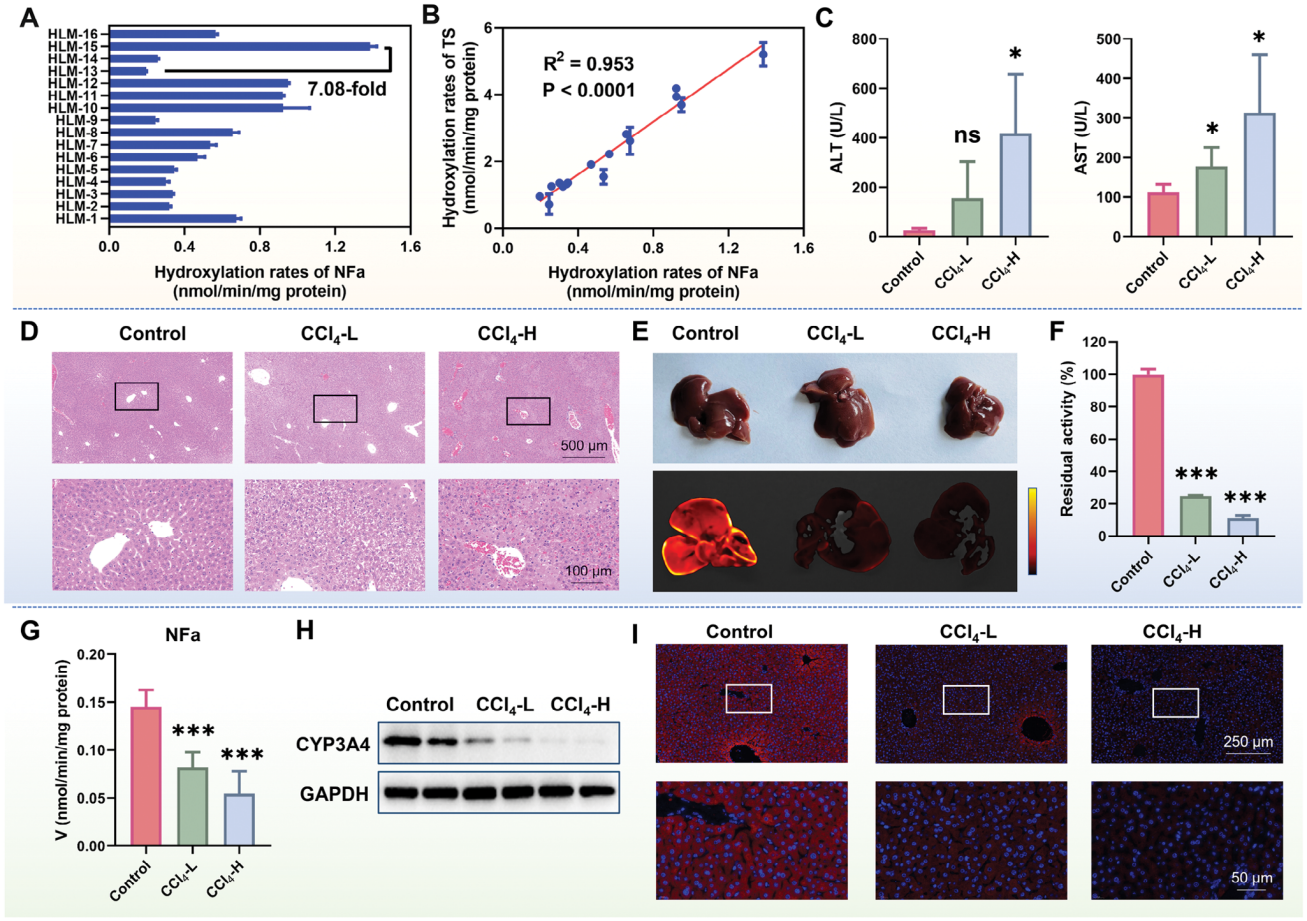
A) Sensing hCYP3A4 activities in individual HLM samples using **NFa** as the substrate. B) Correlation analysis between **NFa** 4‐hydroxylation rates and testosterone (TS) 6β‐hydroxylation rates in a panel of individual HLM samples (n = 16). C) The ALT and AST levels in control and CCl_4_‐induced acute liver injury mice. D) Hematoxylin and eosin (H&E) staining of the liver slices from both control mice and CCl_4_‐induced acute liver injury mice. E) Visualization of CYP3A activity in the liver tissue of control and CCl_4_‐induced acute liver injury mice using **NFa**. F) The activity levels of CYP3A in the liver were determined for both control and CCl_4_‐induced acute liver injury mice. G) The activity levels of CYP3A4 using **NFa** as the fluorogenic substrate. H) CYP3A4 protein expression in liver tissues. I) Immunofluorescence plots of CYP3A4 in liver tissue of control and CCl_4_‐induced acute liver injury mice. Data are expressed as mean ± SD (n = 3–5). ns *P* > 0.05, *P < 0.05, and ***P < 0.001 versus Control.

Subsequently, **NFa** was used as a visualization tool for imaging CYP3A4 activities in liver tissue preparations and organs. First, **NFa** was used as a fluorogenic tool to quantify the real activities of CYP3A4 in a suite of individual HLM samples, and testosterone (a known endogenous substrate for CYP3A4) was also used as a positive substrate. A high correlation coefficient (R^2^ = 0.953) was observed for **NFa** 4‐hydroxylation rates and testosterone 6β‐hydroxylation rates (**Figure** **7**A,B). **NFa** was then used for fluorogenic imaging of endogenous CYP3A in mouse liver tissue slices. As shown in Figure S26 (Supporting Information), following co‐incubation of **NFa** with mouse liver slices under physiological conditions for 1 h, strong green fluorescence signals were observed. By contrast, the green signals could be significantly blocked in liver slices pre‐incubated with RTV or KET (two potent CYP3A inhibitors) for 1 h. It was also found that the fluorescence signals of liver organs collected from CCl_4_‐induced acute liver injury mice (Figure 7C–E) were significantly weakened compared to the control group when using **NFa** as the fluorogenic substrate. Further LC‐FD analysis revealed a significant reduction in the metabolite (**4‐HNFa**) of **NFa** in the liver collected from CCl_4_‐induced acute liver injury mice (Figure 7F), while biochemical and Immunofluorescence (IF) analysis confirmed that the expression levels of CYP3A4 in mice liver could be significantly down‐regulated by CCl_4_ (Figure 7G–I). These findings demonstrate that **NFa** is a practical and high‐performance tool for visualizing CYP3A4 in liver preparations, liver tissue slices, and organs (ex vivo).

### 2.9 In situ Imaging of CYP3A4 in Tumor Xenografted Mice

**Figure 8:**
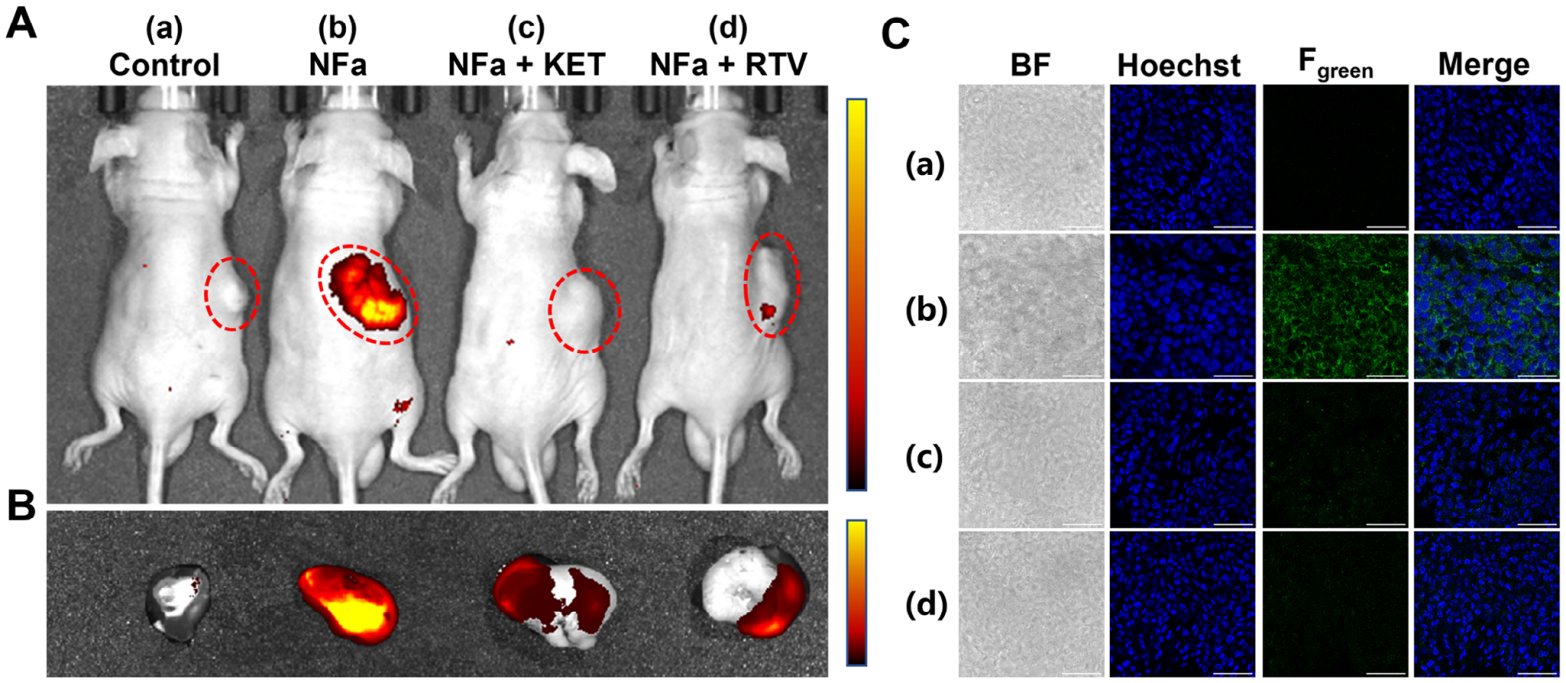
A) In situ imaging of CYP3A in MCF‐7 tumor‐xenografted mice. Mice were intratumorally injected with PBS (a, 100 µL) or **NFa** (b, 50 µm, 100 µL), while the fluorescence images were recorded at 1 h. Mice treated with KET (c, 50 µm, 100 µL) or RTV (d, 50 µm, 100 µL) for 1 h and then incubated with **NFa** (50 µm, 100 µL) for 1 h. The red dotted circle indicates the location of the tumors. B) Fluorescence imaging of tumors removed from mice. C) Fluorescence imaging of CYP3A4 in tumor slices. Tumor slices were treated with PBS (a) or **NFa** (20 µm) for 1 h. Tumor slices in the inhibitor groups were pretreated with KET or RTV for 1 h and subsequently incubated with **NFa** (20 µm) for another 1 h. Scale bar: 50 µm.

Since **NFa** showed exceptional spatial imaging resolution in living cells, tissue slices, and liver organs (ex vivo), **NFa** was further used for in situ imaging of CYP3A4 in tumor‐bearing mice (a subcutaneous tumor model constructed using MCF‐7 cells). As shown in **Figure** **8**A, after intratumoral injection of **NFa** after 1 h, brightly fluorogenic signals from the tumor could be clearly observed. In contrast, fluorogenic signals from the subcutaneous tumors were significantly reduced following treatment with RTV or KET. The subcutaneous tumors were then dissected for fluorescence imaging of CYP3A4. As shown in Figure 8B, upon the addition of **NFa**, strong green fluorogenic signals were generated from the tumors, while such signals could be significantly blocked following treatment with RTV or KET. Meanwhile, the tumors were sliced and then stained with **NFa** for functional imaging of CYP3A4. As expected, bright green fluorogenic signals were observed from tumor slices following incubation with **NFa** for 1 h (Figure 8C), while the green fluorogenic signals could be significantly attenuated by either RTV or KET. These observations clearly demonstrate that **NFa** is a powerful and practical fluorogenic tool for in situ imaging of CYP3A4 in tumors, which can be utilized to in real‐time monitor the dynamical changes of CYP3A4 activity in cancerous tissues and tumor‐xenograft animals. Given that CYP3A4 is one of the key metabolizing enzymes participating in the metabolic clearance of anti‐cancer drugs, the activity levels of CYP3A4 in cancerous tissues varies significantly among different patients. Thus, sensing the activity levels of CYP3A4 in tumors using **NFa** helps facilitate personalized treatment and precision dosing in the clinic.

### 2.10 NFa is used for Screening and Characterizing hCYP3A4 Inhibitors In Vitro and Ex Vivo

**Figure 9:**
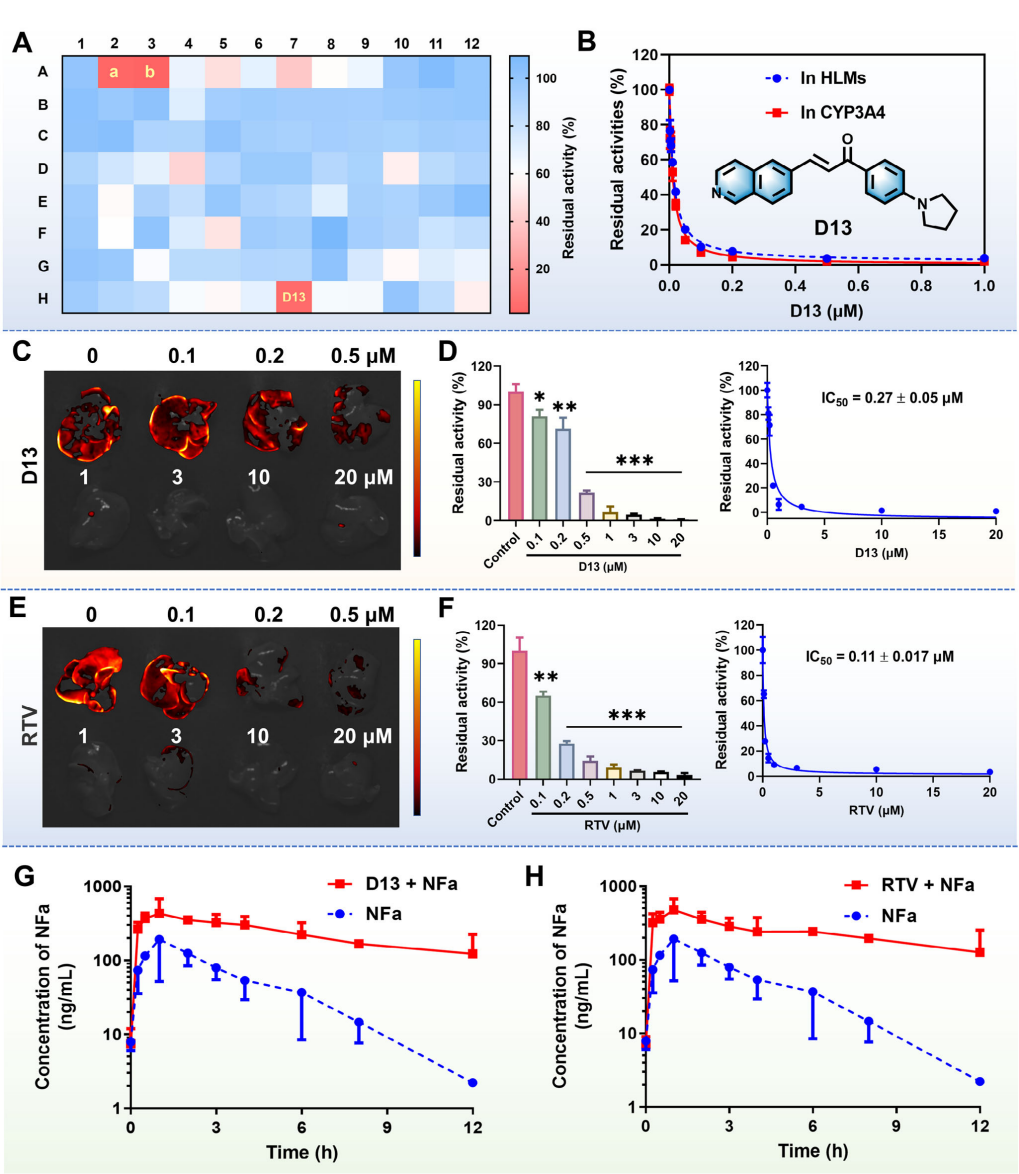
A) The heatmaps for the inhibitory potentials of an in‐house compound library against hCYP3A4 using **NFa** as the fluorogenic substrate (a, KET; b, RTV). B) Dose‐inhibition curves of **D13** against hCYP3A4 in HLMs and hCYP3A4. C) The inhibitory effects of **D13** on CYP3A4 at the liver organ level. D) The metabolite levels (**4‐HNFa**) of **NFa** in liver tissue after treatment with different doses of **D13**. E) The inhibitory effects of RTV on CYP3A4 at the liver organ level. F) The metabolite levels (**4‐HNFa**) of **NFa** in liver tissue after treatment with different doses of RTV. G) The mean plasma exposure‐time curves of **NFa** (20 mg kg^−1^, i.g.) in the control group (CMC‐Na, n = 6) and experimental group (50 mg kg^−1^ of **D13**, *i.g*., n = 6). H) The mean plasma exposure‐time curves of **NFa** (20 mg kg^−1^, *i.g*.) in the control group (CMC‐Na, n = 6) and experimental group (50 mg kg^−1^ of RTV, *i.g*., n = 6). Data are expressed as mean ± SD (n = 3/6). *P < 0.05, **P < 0.01, and ***P < 0.001 versus Control.

**NFa** was then used to construct a fluorescence‐based biochemical assay for high‐throughput screening and characterization of hCYP3A4 inhibitors. As illustrated in Figure S27 and Table S7 (Supporting Information), two positive inhibitors (KET and RTV) could inhibit CYP3A4‐catalyzed **NFa** 4‐hydroxylation in both HLMs and hCYP3A4, validating the applicability of the **NFa**‐based biochemical assay for high‐throughput screening of CYP3A4 inhibitors. Then, the inhibitory potentials of 93 natural products and their derivatives against CYP3A4 were assayed using a final inhibitor concentration of 1 µm. Notably, a chalcone derivative **D13** completely inhibited CYP3A4‐catalyzed **NFa** 4‐hydroxylation at 1 µm in HLMs and displayed similar anti‐hCYP3A4 potency as RTV and KET (**Figure** **9**A; Table S8, Supporting Information). Further investigations confirmed the dose‐dependent inhibition by **D13** of the catalytic activities of CYP3A4 in both HLMs and CYP3A4, with calculated IC_50_ values of 13.58 and 10.88 nm, respectively (Figure 9B). Meanwhile, **D13** demonstrated similar inhibitory effects (25.40 nm) in living cells (Figure S28, Supporting Information). Next, the inhibitory effects of **D13** and RTV on CYP3A4 were visually evaluated at the organ level. As shown in Figure 9C–F, following co‐incubation with N**Fa** in the whole mice liver for 0.5 h, strong fluorescence signals were observed. By contrast, upon the addition of **D13** or RTV to whole mice livers, the fluorescence signals could be dose‐dependently inhibited by **D13** or RTV. These findings suggest that the **NFa**‐based functional imaging assay offers a highly efficient and easy‐to‐use approach for screening and characterizing hCYP3A4 inhibitors at multiple dimensions (in vitro‐living cells‐ex vivo), and the above results encouraged us to further evaluate the inhibitory effect of **D13** on CYP3A4 in vivo.

### 2.11 NFa can be used to Assess In Vivo Anti‐CYP3A4 Effects

As mentioned above, orally available CYP3A4 fluorogenic substrates have not been previously reported. As such, **NFa** was used as an in vivo CYP3A4 fluorogenic substrate to assess the DDI potentials of two CYP3A4 inhibitors (**D13** and RTV). Before in vivo studies, the safety profiles of **NFa** in mice were carefully evaluated. As shown in Figure S29A,B (Supporting Information), no mortality was observed in healthy mice following oral administration of **NFa** at a high dose (100 mg kg^−1^ per day) for 14 consecutive days, while the body weight of the mice showed a gradual increase. Biochemical assays and hematoxylin and eosin (H&E) staining (Figure S29C,D, Supporting Information) demonstrated no haematotoxicity, organ injuries, or apparent abnormalities in the **NFa**‐treated mice, indicating that **NFa** showed favorable safety profiles. Meanwhile, the oral bioavailability of **NFa** in mice was also assayed. As expected, **NFa** (20 mg kg^−1^) could be rapidly absorbed into the circulating system and then metabolized by CYP3A in mice following oral administration (Figure S30 and Table S9, Supporting Information). The oral bioavailability of **NFa** was determined to be 15.3% in mice. Encouraged by these findings, **NFa** was then used to replace CYP3A4‐substrate drugs (such as midazolam) for assessing the in vivo inhibitory effects of two CYP3A4 inhibitors (**D13** and RTV) using mice as surrogate animals. The results indicated that **D13** (50 mg kg^−1^) could significantly elevate the circulating exposure of **NFa** (i.g.) by 7.22‐fold (AUC_(0‐inf)_ ranged from 642 to 4633 ng mL^−1^·h) compared with the control group (CMC‐Na instead of **D13**), while the *C*_max_ value of **NFa** (i.g.) in mice plasma was increased 2.37‐fold (from 210 to 498 ng mL^−1^). Moreover, the metabolic half‐life (*t*_1/2_) of **NFa** (i.g.) was significantly prolonged by 2.87‐fold (from 2.22 h to 6.37 h) (Figure 9G and **Table** **2**). Similarly, compared with the control group, RTV (50 mg kg^−1^, a potent inhibitor of CYP3A4) also significantly elevated the AUC_(0‐inf)_ (9.03‐fold, from 642 to 5795 ng mL^−1^ h) and *C*_max_ (2.35‐fold, from 210 to 494 ng mL^−1^), as well as prolonging the metabolic half‐life (3.64‐fold, from 2.22 h to 8.09 h) of **NFa** (i.g.) in mice plasma (Figure 9H). These findings are consistent with a previous report using midazolam as a drug substrate, in which the AUC_inf_ of midazolam (MDZ, oral administration) is increased by five‐fold when MDZ is co‐administered with RTV (50 mg kg^−1^) in mice.^[^ ^51^ ^]^ These findings clearly show that **D13** is an efficacious CYP3A4 inhibitor in various systems from liver preparations to ex vivo and in vivo, suggesting that this agent can significantly modulate the pharmacokinetic behaviors and the therapeutic outcomes of CYP3A4‐substrate drugs in vivo. More importantly, it is particularly gratifying to note that **NFa** acts as a highly specific and orally available CYP3A4 fluorogenic substrate, which offers a practical and reliable mass‐spectrometry‐independent tool for assessing the in vivo effects of CYP3A4 inhibitors.

**Table 2:** The effects of either **D13** (50 mg kg^−1^, i.g.) or RTV (50 mg kg^−1^, i.g.) on the pharmacokinetic behaviors of **NFa** (20 mg kg^−1^, i.g.) in mice. Data are expressed as mean ± SD (n = 6).

Group	*T*_max_ [h]	*C*_max_ [ng/mL]	AUC_(0‐inf)_ [ng/mL*h]	*t*_1/2_ [h]
CMC‐Na + **NFa**	1.25 ± 0.61	210 ± 125	642 ± 146	2.22 ± 0.94
**D13**+ **NFa**	0.70 ± 0.27	498 ± 209	4633 ± 2231	6.37 ± 4.65
Ratio	0.56	2.37	7.22	2.87
RTV + **NFa**	0.95 ± 0.67	494 ± 131	5795 ± 5005	8.09 ± 7.52
Ratio	0.76	2.35	9.03	3.64

## 3 Conclusion

In summary, an AI‐driven strategy was adapted to rationally develop highly specific and orally active fluorogenic substrates for CYP3A4, resulting in three CYP3A4 substrate candidates bearing drug‐like fragments being designed and synthesized. Specifically, **NFa** was validated as an ideal fluorogenic substrate for hCYP3A4, showing a rapid response, excellent isoform‐specificity, ultra‐high sensitivity, exceptional ER‐colocalization performance, high spatial imaging resolution, and favorable safety profiles. **NFa** exhibited exceptional performance for functional imaging of CYP3A4 in living cells, tissue slices, liver organs, and tumor xenograft mice, as well as good performance for high‐throughput screening of CYP3A4 inhibitors in live cells and organs and assessing the DDI potentials at multiple dimensions. Following oral administration, **NFa** could be rapidly absorbed into the circulating system and then metabolized by CYP3A in mice, while the CYP3A inhibitors (**D13** and RTV) significantly elevated the plasma exposure levels of **NFa**. Collectively, this study demonstrates a novel AI‐driven strategy for constructing highly specific and orally available fluorogenic substrates for a target enzyme, generating **NFa** as a practical and reliable tool for sensing and imaging of CYP3A4 activities in various living systems, which will greatly facilitate CYP3A4‐associated fundamental investigations, inhibitor discovery, and translational research.

## 4 Experimental Section

A detailed description of materials and experimental methods can be found in the Supporting Information.

### Data Collection and preparation

Chemical data for model development and evaluation were sourced from the PubChem Bioassay Database and the ChEMBL Database. Within the PubChem Database, all data were classified as either “active” (inhibitors) or “inactive” (non‐inhibitors), with a cutoff set at pIC_50_ = 5. In the ChEMBL dataset, each compound had specific pIC_50_ labels. To enhance the dataset, the same cutoff criteria were applied in the PubChem dataset to partition the data points in the ChEMBL database. Subsequently, “active” (inhibitors) and “inactive” (non‐inhibitors) categories were classified. Standardization procedures were implemented on all SMILES in the dataset to ensure consistency and facilitate analysis. This involved removing fragments, metal ions, and stereochemical information, as well as converting the data to the Canonical SMILES format and normalizing it. Following these processing steps, a comprehensive dataset comprising 27045 compounds with classification labels was assembled.

### AFP‐3A4 Framework and Training

The overall model architecture of AFP‐3A4 is illustrated in Figure 1. A model architecture and training approach was used based on the open‐source AttentiveFP (AFP) framework. The AFP algorithm represents a specialized graph neural network with a graph attention mechanism, demonstrating outstanding predictive performance across various datasets. Notably, this algorithm excels in capturing nonlocal intramolecular interactions and visualizing acquired knowledge. The hyperparameters and retrained model were adjusted using the complete CYP3A4 dataset. The ultimate output of the model was the inhibition probability of CYP3A4, where values exceeding 0.5 were classified as “active” (inhibitors), while values below 0.5 were deemed “inactive” (non‐inhibitors). Additionally, the atomic weights within each compound were updated post‐model prediction. The model's performance was evaluated using classification metrics such as Area Under the Receiver Operating Characteristic Curve (AUC‐ROC), Area Under the Precision–Recall Curve (AUPR), Precision (PR), and Recall (RE). In essence, an optimal model should exhibit high AUC‐ROC, AUPR, PR, and RE values, as illustrated in Figure S31 (Supporting Information).

### Key Fragment Analysis

The well‐trained AFP‐3A4 model can provide the contribution score of each atom by utilizing atom attention weights. These weights measure the significance of each atom in the final molecular representation, and the sum of attention weights for all atoms in a molecule was equal to 1. Consequently, key fragments were defined as substructures composed of atoms with significantly higher atom attention weights than the rest. In this study, a candidate dataset was initially selected from the complete CYP3A4 dataset using the improved Lipinski's rule of five (250 < MW < 750; H Acceptors < 10; H Donors < 5; −2 < cLogP < 7; TPSA < 150 Å^2^)^[^ ^49^, ^50^ ^]^ and CYP3A4 inhibitor activity screening (IC_50_ < 100 nm). The approach outlined was followed in the IDL‐PPB and extracted key fragments from the candidate dataset using the well‐tuned AFP model and the Wilcoxon test method (Figure S32, Supporting Information). This method consisted of three main steps: molecular fragmentation, statistical testing, and redundancy removal. First, the molecules were fragmented into segments of 5–13 carbon atoms without breaking any ring bonds (denoted as A at breakpoints).^[^ ^52^ ^]^ Second, a statistical test was conducted to determine if the atom weights of each substructure were significantly higher than the remaining part of the molecule (i.e., *P* < 0.05). Only those fragments passing the one‐sided Wilcoxon test were recorded for subsequent analysis. For substructure matching and the Wilcoxon test, the Python packages RDKit (version 2018.09.3.0) and SciPy (version 1.6.2) were utilized, respectively. Finally, in cases where redundant substructure fragments were encountered, only the largest substructure was retained as it contained more structural information.

### Animal Experiments

SPF grade C57BL/6J mice were purchased from Shanghai SLAC Laboratory Animal Co. Ltd and housed under controlled environmental conditions (22 ± 2 °C; 40–80% relative humidity; 12 h light/dark cycle) with free access to food and water. The ethical approval was provided by the Animal Care and Use Committee of the Shanghai Institute of Food and Drug Control (the approval number: IACUC‐SIFDC23066; IACUC‐SIFDC24002). All mice were subjected to adaptive feeding for 7 days before the experiment.

### Functional Imaging of hCYP3A4 in CCl_4_‐Induced Liver Injury in Mice

C57BL/6J mice (n = 18, ♂, weighing ≈20 g) were housed under controlled environmental conditions (22 ± 2 °C; 40–80% relative humidity; 12 h light/dark cycle) for one week with free access to food and water. The mice were randomly divided into three groups: the control group, CCl_4_ low dose group (CCl_4_‐L, 0.15%), and CCl_4_ high dose group (CCl_4_‐H, 0.3%). CCl_4_ was dissolved in vegetable oil and administered via intraperitoneal injection. The control group was injected intraperitoneally with an equal volume of vegetable oil. After administration, mice were fasted and allowed to drink water freely. After 18 h, blood and liver tissue were collected. Liver tissues were washed 2–3 times with PBS and then soaked in a solution containing **NFa** (50 µm, 5 mL). After 1 h, liver tissues were removed for organ imaging. The imaged liver tissues were ground, centrifuged, and the supernatant was taken, and 10‐fold the volume of acetonitrile was added to precipitate the protein. After centrifugation again, the supernatant was taken and the amount of metabolite of **NFa** in the liver tissue was quantified.

### Safety Test of NFa in Healthy Mice

C57BL/6J mice (n = 12, 6♂6♀, weighing ≈20 g) were housed under controlled environmental conditions (22 ± 2 °C; 40–80% relative humidity; 12 h light/dark cycle) for one week with free access to food and water. **NFa** was suspended in 0.5% CMC‐Na before oral administration. The mice were randomly divided into two groups (n = 6, CMC‐Na and **NFa**). Mice were orally administered **NFa**/CMC‐Na (100 mg kg^−1^) continuously for 14 days, and their status was observed, and their weight was measured daily. After the last oral administration of **NFa**/CMC‐Na for 24 h, blood samples were collected to test the biochemical index, while the mice organs, including heart, liver, spleen, lung, kidney, large intestine, small intestine, and brain were harvested for hematoxylin and eosin (H&E) staining to identify histopathological abnormalities.

### Liver Organ Imaging in the Presence of CYP3A4 Inhibitor

The mice were executed, and all liver tissues were removed and soaked in RTV or **D13** solution (0, 0.1, 0.2, 0.5, 1, 3, 10, 20 µm) at 37 °C for 1 h. Next, **NFa** (50 µm) was added to the solution containing liver tissues and incubated at 37 °C for 30 min. After that, the liver tissues were removed, and fluorescence imaging of the liver was performed using VISQUE Invivo Smart‐LF. After organ imaging, the tissues were ground, and centrifuged, and the supernatant was diluted with 10‐fold acetonitrile to precipitate the protein. After centrifugation again, the supernatant was determined to quantify the level of **4‐HNFa**.

### Statistical Analysis

All data were expressed as the mean ± standard deviation. The IC_50_, *K*_m_, and *V*_max_ values were determined via non‐linear regression analysis using GraphPad Prism software. The t‐test was employed for group comparisons, with statistical significance set at *P* < 0.05. All statistical analyses were carried out using GraphPad Prism software (GraphPad Prism 8.0).

## Conflict of Interest

The authors declare no conflict of interest.

## Author Contributions

F.Z., L.S., and R.W. contributed equally to this work. G.G. developed the concepts and designed the experiments; F.Z., L.S., R.W., and J.H. performed the experiments and processed the experimental data; F.Z., G.G., Y.F., and H.L. analyzed the results and drafted the manuscript. G.G., T.D.J., X.Y., H.Z., Z.J., B.Z., X.Y., and L.W. contributed to the writing of the final version of the manuscript. All authors read and approved the final manuscript.

## Supporting information



Supporting Information

## Data Availability

The data that support the findings of this study are available in the supplementary material of this article.
