# Supplementary material for: Functional Imaging of CYP3A4 at Multiple Dimensions Using an AI‐Driven High Performance Fluorogenic Substrate
Source: Small. 2025 Mar 21;21(17):2412178. doi: 10.1002/smll.202412178 (PMC12036557; doi:10.1002/smll.202412178)
Supplement: Supplementary file 1 — Supporting Information [file SMLL-21-2412178-s001.docx]

**Supplementary Information**

***for***

**Functional Imaging of CYP3A4 at Multiple Dimensions Using an AI-driven High Performance Fluorogenic Substrate**

*Feng Zhang ^†^, Lilin Song ^†^, Ruixuan Wang ^†^, Bei Zhao, Jian Huang, Luling Wu, Yufan Fan, Hong Lin, Zhengtao Jiang, Xiaodi Yang, Hairong Zeng, Xin Yang, Tony D. James*, and Guangbo Ge**

F. Zhang, R. Wang, B. Zhao, J. Huang, Y. Fan, Z. Jiang, H. Zeng, Prof. G. Ge

State Key Laboratory of Discovery and Utilization of Functional Components in Traditional Chinese Medicine, Shanghai Frontiers Science Center of TCM Chemical Biology, Institute of Interdisciplinary Integrative Medicine Research, Shanghai University of Traditional Chinese Medicine, Shanghai 201203, China

Email: [geguangbo@shutcm.edu.cn](mailto:geguangbo@shutcm.edu.cn)

L. Song

Liaoning Provincial Key Laboratory of Carbohydrates, Dalian Institute of Chemical Physics, Chinese Academy of Sciences, Dalian, 116023, China

J. Huang

Pharmacology and Toxicology Division, Shanghai Institute of Food and Drug Control, Shanghai, 201203, China

Dr. L. Wu, Prof. TD James

Department of Chemistry, University of Bath, Bath BA2 7AY, United Kingdom. E-mail: [t.d.james@bath.ac.uk](mailto:t.d.james@bath.ac.uk)

Prof. TD James

School of Chemistry and Chemical Engineering. Henan Normal University, Xinxiang 453007, China.

H. Lin, Prof. X. Yang

Innovation Research Institute of Traditional Chinese Medicine, Shanghai University of Traditional Chinese Medicine, Shanghai, 201203, China

Prof. X. Yang

Department of Electrical and Electronic Engineering, School of Engineering, Cardiff University, Cardiff, CF24 3AA, United Kingdom

^[†]^ These authors contributed equally to this work.

**This file contains one scheme, eighty supplementary figures and nine supplementary tables.**

**1. Chemical synthesis**

**Scheme S1**. Synthetic routes of both **NFs** and **4-HNFs**.

**Protocol A**

1.0 eq of naphthalic anhydride and 2.0 eq of 4-thiazolemethanamine hydrochloride or its derivatives were dissolved in ethanol, add equal equivalents of DIEA to neutralize HCl. The mixture was refluxed at 80 ℃ for 2 h and cooled down to room temperature. The precipitate was collected by filtration and purified by column chromatography on silica gel (1% methanol/dichloromethane) to obtain pure products.

**Protocol B**

1.0 eq of 4-bromo-1,8-naphthalic anhydride and 2.0 eq of 4-thiazolemethanamine hydrochloride or its derivatives were dissolved in ethanol, add equal equivalents of DIEA to neutralize HCl. The mixture was refluxed at 80 ℃ for 2 h and cooled down to room temperature. The precipitate was collected by filtration to obtain intermediate 4-bromo-*N*-(4-thiazolemethanamino)-naphtahlimide or its derivates, it was used for the next reaction without purification.

1.0 eq of 4-bromo-*N*-(4-thiazolemethanamino)-naphtahlimide or its derivates, 3.0 eq of *N*-hydroxy succinimide (NHS), and 3.0 eq of potassium carbonate were dissolved in dimethyl sulfoxide, the mixture was refluxed at 80 ℃ for 2 h and poured into 0.1 M hydrochloric acid aqueous solution, the precipitate was collected by filtration and purified by column chromatography on silica gel (ethyl acetate/petroleum ether) to obtain pure product.

**2. Experimental Section**

***Chemicals, enzymes, and reagents***

All chemical reagents were obtained from Sinopharm Chemicals (Shanghai, China). D-glucose-6-phosphate (G-6-P), glucose-6-phosphate dehydrogenase (G-6-PDH), and β-NADP^+^, ABT, furafylline, tranylcypromine, montelukast, sulfaphenazolum, quinidine, clomethiazole were obtained from Sigma-Aldrich (St. Louis, MO, USA). CYP3cide was purchased from MedChemExpress (USA). MgCl_2_ was supplied by Sinopharm Chemical Reagent (Shanghai, China). Pooled human liver microsomes from 50 donors (HLMs, lot No. X008067) were purchased from Bioreclamation IVT (Baltimore, MD, USA). A panel of human recombinant cytochrome P450 enzymes (such as CYP1A1, CYP1A2, CYP1B1, CYP2A6, CYP2A13, CYP2B6, CYP2C8, CYP2C9, CYP2C18, CYP2C19, CYP2D6, CYP2E1, CYP2J2, CYP3A4, CYP3A5, CYP4A11, CYP4F2) were obtained from Cypex. 16 individual-donor HLM were supplied by the Research Institute for Liver Diseases (RILD, Shanghai, China). LC grades of methanol, acetonitrile, and formic acid were ordered from Fisher Scientific Co. (Fair Lawn, NJ, USA), while ultra-purified water was prepared using a Millipore purification system. Ketoconazole, ritonavir, DMEM Medium, RPMI 1640 Medium, and PBS were obtained from Meilun Bio. Tech (Dalian, China). Cell Counting Kit-8 (CCK-8) was supplied by Yeasen Biotechnology (Shanghai) Co., Ltd. Fetal bovine serum (FBS) and trypsin were purchased from Gibco. Hoechst 33342 was supplied by Shanghai Yuanye Biotechnology Co., Ltd (Shanghai, China), while ER-tracker Red was purchased from Beyotime Biotechnology. Antibody against CYP3A4 was provided by Proteinteck. Antibody against GAPDH was purchased by Cell Signaling Technology. All natural products and derivatives were synthesized using previously reported protocols.

***Molecular docking simulations***

The X-ray crystal structures of several hCYP3A4 enzymes were utilized for docking simulations. The MM2 force field in ChemBio 3D Ultra 15.0 was utilized to obtain the energy-minimized conformation of the compounds. The proteins and ligands were prepared utilizing AutoDock Vina (1.1.2), with adjustments limited to polar hydrogen atoms, Kollman atomic charges, and AD4 atom types. Molecular docking simulations were performed by utilizing AutoDock Vina 1.1.2 in a grid covering the entire active site. The catalytic conformation was depicted in PyMol (Version 2.3, Schrödinger, LLC, New York City, USA).

***Metabolic profiling of NFs in HLMs***

The incubation system (200 μL) contained PBS (pH 7.4, 100 mM), enzyme sources, NADPH regeneration system (1.0 mM β-NADP^+^, 4.0 mM MgCl_2_, 10 mM G-6-P, 1.0 U/mL G-6-PDH) and **NFs** (20 μM). After incubating for 30 min, the reaction was terminated by adding 200 μL ice-cold acetonitrile. All samples were kept on ice until centrifugation at 20,000 ×g and 4 °C for 30 min. After that, the supernatants were taken for further analysis.

The metabolites of **NFs** in HLMs were identified by the Shimadzu LC system coupled with a Triple TOF 5600 mass spectrometer (SCIEX). The mobile phase consisted of 0.1% formic acid in water (A) and acetonitrile (B) with the following elution program: 0-2 min, 2% B; 2-10 min, 2%-90% B; 10-12 min, 90% B; 12-12.5 min, 90%-2% B; 12.5-15.0 min, 2% B. The flow rate was set at 0.4 mL/min, while the column temperature was maintained at 40 ºC. The full-scan mass was acquired over the range from 50-1500 (*m/z*). The ion source gas1 and gas2 were 50 psi, the curtain gas was 35 psi, the ion spray voltage was set at +5000 kV/-4500 kV, and the ion source temperature was 500/450 ºC. The declustering potential (DP) and collision energy (CE) were ±80 V and ±45 V, respectively.

***P450*** ***reaction phenotyping analysis and chemical inhibi******tion assays***

The metabolic rates of **NFs** in a panel of human recombinant cytochrome P450 enzymes were determined in the presence of NADPH regeneration system (1.0 mM β-NADP^+^, 4.0 mM MgCl_2_, 10 mM G-6-P, 1.0 U/mL G-6-PDH). The final concentration of each human recombinant CYP enzyme in the incubation system was 2.5 nM. Each sample was co-incubated with **NFs** at 37 °C for 30 min, the supernatants were utilized to quantify the formation rates of **4-HNFs** using HPLC-FD.

A group of specific inhibitors of human P450s were utilized to assess the inhibitory effects on **NFs** 4-hydroxylation in HLMs. The final concentrations of ABT, furafylline, tranylcypromine, thiotepa, montelukast, sulfaphenazolum, quinidine, clomethiazole, ketoconazole and CYP3cide were 500 μM, 10 μM, 1 μM, 5 μM, 1 μM, 10 μM, 10 μM, 10 μM, 1 μM, 2 μM, respectively. LC-FD was utilized to determine the formation rate of **4-HNFs** in the presence or absence of inhibitors.

***Density functional theory (DFT) calculations***

Preliminary, a GMMX conformational analysis is performed by the Gaussian program using a DFT/B3LYP/6-31G(d) pre-optimized 3D geometry. The resulting GMMX conformers are optimized at the B3LYP/6-31G(d) level. And all conformers are real minima, as no imaginary vibrational frequencies are found. The energy ranking is performed to find the minimum energy conformer. The minima conformer is optimized at B3LYP /6-311G(d,p) level.^[1-3]^

***Oxidative metabolism of NFa in FMOs***

The incubation system (200 μL) contained PBS (pH 7.4, 100 mM), enzyme sources (FMO1, FMO3, FMO5), NADPH regeneration system (1.0 mM β-NADP^+^, 4.0 mM MgCl_2_, 10 mM G-6-P, 1.0 U/mL G-6-PDH) and **NFa** (20 μM).^[4]^ After incubating for 30 min, the reaction was terminated by adding 200 μL acetonitrile. All samples were kept on ice until centrifugation at 20,000 ×g and 4 °C for 30 min. After that, **NFa** and **4-HNFa** in supernatants were quantified using HPLC-UV.

***Oxidative metabolism of NFa in AO***

The incubation system (200 μL) contained PBS (pH 7.4, 100 mM, containing 0.1 mM EDTA), human liver cytosol, and **NFa** (20 μM).^[5]^ After incubating for 30 min, the reaction was terminated by adding 200 μL acetonitrile. All samples were kept on ice until centrifugation at 20,000 ×g and 4 °C for 30 min. After that, the remaining **NFa** and the formation rates of **4-HNFa** in the supernatants were quantified using HPLC-UV.

***Biosynthesis and purification of the oxidative metabolite of NFa***

The oxidative metabolite of **NFa** was biosynthesized using mice liver microsomes mixed with human liver microsomes (95:5) and then purified by using HPLC equipped with an ODS column. Briefly, the incubation system (2 L) contained PBS (pH 7.4, 100 mM), liver microsomes (0.05 mg/mL, final concentration), NADPH regeneration system (1.0 mM β-NADP^+^, 4.0 mM MgCl_2_, 10 mM G-6-P, 1.0 U/mL G-6-PDH) and **NFa** (12 mg). After incubating for 2 h, all samples were centrifugated (20,000 ×g) at 4 °C for 30 min. The supernatants were concentrated and then isolated by HPLC equipped with an ODS column (2.0 × 250 mm, 5 μm, Shimadzu) and a UV detector, while methanol and water were used as the mobile phase. The LC fraction containing **4-HNFa** was collected and then dried under a vacuum. The oxidative metabolite of **NFa** was then characterized by both ^1^H-NMR and ^13^C-NMR.

***Metabolic stability assay of 4-HNFa in phase II metabolizing system***

For the phase II metabolic stability assay, Tris-HCl (pH 7.4, 50 mM), HLMs (0.1 mg/mL), Brij58 (0.5 mg/mg protein), MgCl_2_ (5 mM), while **4-HNFa** (20 μM) were mixed at 37 ºC for 3 min, followed by addition of UDPGA (2 mM, final concentration) to initiate the reaction. Samples were collected following incubation at 37 ºC at different times (0, 5, 15, and 30 min) and then mixed with an equal volume of ice-cold acetonitrile. Subsequently, the samples were centrifuged at 20,000 g for 30 min at 4 ºC, and the supernatant (10 μL) was subjected to LC-UV for quantifying **NFa**. All tests were conducted in triplicates throughout the study.

***Enzyme kinetic analyses***

The kinetic parameters of **NFs** 4-hydroxylation in CYP3A4 were determined by performing a set of hydroxylation kinetic assays. Kinetic parameters (*K*_m_ and *V*_max_) for **NFs** 4-hydroxylation were fitted by the following equation (a):

V=(V_max_*×*[S])/(K_m_+[S]) (a)

Here, *K_m_* is the Michaelis constant of **NFs** 4-hydroxylation, *V_max_* is the maximum velocity of **NFs** 4-hydroxylation, *V_max_*/*K_m_* is utilized to calculate the intrinsic clearance (*CL_int_*).

***Cell-membrane permeability***

NCM460 cells were cultured with the RPMI 1640 medium containing 10% FBS in a humidified atmosphere of 5% CO_2_ at 37 °C, Hep3B cells were cultured with the DMEM medium containing 10% FBS in a humidified atmosphere of 5% CO_2_ at 37 °C. After the cells were grown to about 80% confluent in the culture dish, the cells were trypsinized and resuspended with the RPMI 1640/DMEM basal medium to density at 2×10^5^ cells/mL. 200 μL cell suspension was incubated with **NFa**/**NEN/F8** (20 μM) at 37℃ for 60 min. Then, the cells were centrifuged (1000 rpm, 3 min), resuspended in 1640/DMEM basal medium, and centrifuged again to wash out the left **NFa**/**NEN/F8**. Next, the cells were lysed with ice-cold methanol (200 μL) and centrifuged (20000 rpm, 30 min, 4 ℃). The supernatant was then analyzed by HPLC-FD.

***Identification of the P-gp substrates in living cells***

To assess whether **NEN**, **F8**, **and NFa** are the substrates for P-gp, MDR1-MDCK cells were grown in DMEM medium supplemented with 10% fetal bovine serum (FBS), in a humidified atmosphere (95% air and 5% CO_2_) at 37 °C. Then, the cells were seeded in 96-well plates. After 24 h incubation, verapamil (a potent P-gp inhibitor, 100 μL) was added and cultured for 15 min. After that, the cells were treated with **NEN/F8/NFa** (100 μL) for 2 h, the supernatant was then discarded. The cells were washed three times with PBS, followed by 200 µL ice-cold acetonitrile to lyse the cells, and the cell suspension was collected by centrifugation at 20,000 g, 4 °C for 30 min. The supernatant was subjected to LC-FD analysis.

***Functional imaging of hCYP3A4 in living cells***

The functional imaging of hCYP3A4 in living cells was also conducted by confocal microscopy. In brief, Hep3B or MCF-7 cells were seeded in a 35 mm glass bottom dish with 4 chambers at a density of 1 × 10^5^ cells per chamber and cultured in DMEM medium containing 10% FBS for 24 h. With/without ketoconazole or ritonavir (20 μM, final concentration), Hep3B or MCF-7 cells were cultured in basal medium containing DMSO/**NFa** (20 μM, final concentration, 1 h) and Hoechst 33342 (10 μg/mL, final concentration, 15 min), respectively. Then, the cells were imaged using confocal microscopy after fixation with 4% paraformaldehyde (PFA). Blue channel for Hoechst 33342: λ_ex_ 405 nm, λ_em_ 425-475 nm; green Channel for **4-HNFa**: λ_ex_ 488 nm, λ_em_ 530-580 nm; Red channel for ER-Tracker Red: λ_ex_ 552 nm, λ_em_ 590-640 nm. The Pearson's coefficient was calculated utilizing Image Pro Plus.

***Functional imaging of CYP3A4 in tissue slices***

The functional imaging of CYP3A4 in tissue slices was also conducted using confocal microscopy. After fixing the liver tissue of the mouse, slice them into 150 μm with the freezing microtome and place them in the confocal dishes with 2 mL PBS. The inhibitor groups were pre-incubated with ketoconazole or ritonavir (20 μM, final concentration) for 1 h, DMSO/**NFa** (20 μM, final concentration, 1 h), and Hoechst 33342 (10 μg/mL, final concentration, 15 min) was then added. Next, the fluorogenic images were acquired using a confocal microscope. Blue channel for Hoechst: λ_ex_ 405 nm, λ_em_ 425-475 nm; Green channel for **4-HNFa**: λ_ex_ 488 nm, λ_em_ 530-580 nm.

***Tumor Imaging of NFa in BALB/c nude mice***

The expression of CYP3A4 in MCF-7 cell lines was much higher than that in other cells, so MCF-7 cells were selected to prepare the tumor model of BALB/c nude mice. The ethical approval was provided by the Animal Care and Use Committee of Shanghai University of Traditional Chinese Medicine (the approval number: PZSHUTCM2301050004). To prepare the MCF-7 tumor model of BALB/c nude mice, 100 μL PBS containing 1 × 10^6^ MCF-7 cells were subcutaneously injected into the right axilla of each BALB/c nude mouse. After 1 week, MCF-7 subcutaneous tumors formed. Imaging and resection were performed by intratumoral injection **NFa** on tumors. The inhibitor group was injected with KET and RTV (50 μM, 100 μL) 1 h in advance, and the blank group and probe group were injected with equal volume PBS. λ_ex_ 465 nm.

***Detection of the plasma levels of both NFa and 4-HNFa***

For **NFa**: the plasma samples (20 μL) were mixed with acetonitrile in a ratio of 1:5 and fractionated at 20,000 ×g for 30 min at 4 °C. And the supernatant was utilized for HPLC-FD analysis. The quantitative calibration of the **NFa** range was 0-1000 ng/mL.

For **4-HNFa**: all plasma samples were collected from C57 mice and added without/with β-glucuronidase (GUS), and incubated for 30 min. Then, the samples were mixed with ice-cold acetonitrile in a ratio of 1:5 and fractionated at 20,000 ×g for 30 min at 4 °C. The supernatant was then subjected for HPLC-FD analysis. The quantitative calibration of the **4-HNFa** range was 0-1000 ng/mL.

***Screening and characterizing of hCYP3A4 inhibitors***

The inhibitory effects of over 93 natural constituents and their derivatives against CYP3A4 were assessed using **NFa** as substrate. In brief, PBS (pH 7.4, 100 mM), inhibitors (1 μM)/solvent, NADPH regeneration system, and HLMs were sequentially added to a 96-well plate. The samples were then vortexed and pre-incubated for 3 min at 37 °C. Subsequently, the reactions were initiated by adding **NFa** to achieve a final volume of 200 μL. Then, the formation rates of **4-HNFa** were continuously monitored for 30 min at 37 °C utilizing a fluorescence microplate reader (SpectraMax® iD3, Molecular Devices, Austria). The percentage of organic solvent in all incubations was not over 1%.

***The inhibitory effect of D13 in*** ***CHO-3A4 stably transfected cell line***

To assess the inhibitory effects of **D13** on CYP3A4 in living cells, CHO-3A4 stably transfected cell line was grown in F12 medium supplemented with 10% fetal bovine serum (FBS), in a humidified atmosphere (95% air and 5% CO_2_) at 37 °C. CHO-3A4 stably transfected cell line was seeded in 96-well plates. When the cells in 96-well plates were about 60% confluent, they were treated with **D13** for 1 h. After that, the cells were treated with **NFa** (10 μM, final concentration) for 30 min, then terminated by adding 100 μL ice-cold acetonitrile. The reaction mixture was centrifuged at 20,000 ×g for 30 min. The supernatant was subjected to LC-FD analysis.

***In vivo inhibitory effect of D13 against CYP3A4***

The ethical approval was provided by the Animal Care and Use Committee of the Shanghai Institute of Food and Drug Control (the approval number: IACUC-SIFDC23066). The 108 C57 mice (weighing around 20 g) were housed under controlled environmental conditions (22 ± 2°C; 40%-80% relative humidity; 12 h light/dark cycle) for one week with free access to food and water. **NFa**, **D13**, and RTV were suspended in CMC-Na. **D13** and RTV were administered orally at a dose of 50 mg/kg. The oral dose of **NFa** is 20 mg/kg. The 108 mice were randomly divided into three groups (CMC-Na + **NFa; D13** + **NFa;** RTV + **NFa**), with 36 mice in each group. Then 36 mice were immediately divided into 6 cages, 6 mice in each cage. CMC-Na, **D13,** or RTV was administered orally. After 30 min, **NFa** was administered orally. Blood samples were collected at 0, 5, 15, 30 min, 1, 2, 3, 4, 6, 8, and 12 h, respectively. Then, blood samples were centrifuged at 8000 rpm, 4 °C for 10 min, the supernatant was stored at -80 °C until analysis.

***Western blotting***

Cell/tissue samples were initially rinsed twice with PBS before being lysed in RIPA Lysis Buffer, supplemented with a protease inhibitor cocktail. Protein concentrations in the lysates were determined using the Enhanced BCA Protein Assay Kit. For protein analysis, 30 μg of protein per lane was loaded onto 10% sodium dodecyl sulfate-polyacrylamide gels for separation, followed by transfer onto PVDF membranes. The membranes were blocked using 5% nonfat milk and then incubated overnight at 4°C with primary antibodies: anti-CYP3A4, and anti-GAPDH. Subsequently, they were incubated with horseradish peroxidase-conjugated goat anti-rabbit secondary antibodies. Protein bands were visualized using Enhanced Chemiluminescence.

*N*-(4-Thiazolemethanamino)-naphtahlimide (**NFa**)

**NFa** was synthesized by protocol A, white powder, yield 27%, obtain 0.0827 g. **^1^H NMR** (400 MHz, DMSO-*d*_6_) *δ* 9.01 (d, *J* = 2.1 Hz, 1H), 8.54 – 8.48 (m, 4H), 7.90 (dd, *J* = 8.3, 7.2 Hz, 2H), 7.57 (dd, *J* = 2.1, 1.1 Hz, 1H), 5.40 (d, *J* = 1.1 Hz, 2H). **^13^C NMR** (101 MHz, DMSO) *δ* 163.75, 154.48, 153.01, 135.00, 131.86, 131.40, 128.02, 127.76, 122.52, 115.50, 40.24. **HRMS** (ESI): C_16_H_10_N_2_O_2_S [M+H]^+^, *m/z*: calculated: 295.0542, found: 295.0559.

*N*-(2-Methyl-4-thiazolemethanamino)-naphtahlimide (**NFb**)

**NFb** was synthesized by protocol A, white powder, yield 49%, obtain 0.233 g. **^1^H NMR** (400 MHz, DMSO-*d*_6_) *δ* 8.53 – 8.47 (m, 4H), 7.89 (dd, *J* = 8.3, 7.2 Hz, 2H), 7.29 (d, *J* = 1.1 Hz, 1H), 5.29 (d, *J* = 1.1 Hz, 2H), 2.59 (s, 3H). **^13^C NMR** (101 MHz, DMSO) *δ* 165.71, 163.68, 151.66, 134.97, 131.84, 131.39, 128.00, 127.74, 122.51, 114.91, 40.27, 40.24, 19.17. **HRMS** (ESI): C_17_H_12_N_2_O_2_S [M+H]^+^, *m/z*: calculated: 309.0698, found: 309.0696.

*N*-(2-Isopropyl-4-thiazolemethanamino)-naphtahlimide (**NFd**)

**NFd** was synthesized by protocol A, white powder, yield 73%, obtain 0.277 g. **^1^H NMR** (400 MHz, DMSO-*d*_6_) *δ* 8.50 (ddd, *J* = 10.9, 7.8, 1.2 Hz, 4H), 7.89 (dd, *J* = 8.3, 7.3 Hz, 2H), 7.28 (d, *J* = 1.2 Hz, 1H), 5.30 (d, *J* = 1.2 Hz, 2H), 1.30 (d, *J* = 7.0 Hz, 6H). **^13^C NMR** (101 MHz, DMSO) *δ* 177.32, 163.75, 151.53, 134.97, 131.85, 131.38, 128.05, 127.73, 122.54, 113.74, 40.65, 32.88, 23.36. **HRMS** (ESI): C_19_H_16_N_2_O_2_S [M+H]^+^, *m/z*: calculated: 337.1011, found: 337.1014.

4-Hydroxyl-*N*-(4-thiazolemethanamino)-naphtahlimide (**4-HNFa**)

**4-HNFa** was synthesized by protocol B, yellow powder, yield 47% (the final step), obtain 0.203 g. **^1^H NMR** (400 MHz, DMSO-*d*_6_) *δ* 11.95 (s, 1H), 9.00 (d, *J* = 2.0 Hz, 1H), 8.57 (dd, *J* = 8.3, 1.3 Hz, 1H), 8.50 (dd, *J* = 7.3, 1.3 Hz, 1H), 8.38 (d, *J* = 8.2 Hz, 1H), 7.79 (dd, *J* = 8.4, 7.2 Hz, 1H), 7.49 (d, *J* = 2.0 Hz, 1H), 7.19 (d, *J* = 8.2 Hz, 1H), 5.37 (d, *J* = 1.0 Hz, 2H). **^13^C NMR** (101 MHz, DMSO) *δ* 164.04, 163.31, 160.94, 154.38, 153.33, 134.27, 131.83, 129.88, 129.59, 126.17, 122.95, 122.29, 115.34, 113.03, 110.50. **HRMS** (ESI): C_16_H_10_N_2_O_3_S [M+H]^-^, *m/z*: calculated: 309.0333, found: 308.9988.

4-Hydroxyl-*N*-(2-methyl-4-thiazolemethanamino)-naphtahlimide (**4-HNFb**)

**4-HNFb** was synthesized by protocol B, yellow powder, yield 32% (the final step), obtain 0.141 g. ^1^H NMR (400 MHz, DMSO-*d*_6_) *δ* 11.95 (s, 1H), 8.57 (dd, *J* = 8.4, 1.3 Hz, 1H), 8.50 (dd, *J* = 7.2, 1.3 Hz, 1H), 8.38 (d, *J* = 8.2 Hz, 1H), 7.79 (dd, *J* = 8.3, 7.2 Hz, 1H), 7.23 – 7.15 (m, 2H), 5.29 – 5.24 (m, 2H), 2.59 (s, 3H). **^13^C NMR** (101 MHz, DMSO) *δ* 165.61, 163.99, 163.26, 160.91, 151.98, 134.27, 131.84, 129.87, 129.58, 126.17, 122.94, 122.29, 114.74, 113.04, 110.49, 19.17. **HRMS** (ESI): C_17_H_12_N_2_O_3_S [M+H]^-^, *m/z*: calculated: 323.0490, found: 323.0139.

4-Hydroxyl-*N*-(2-isopropyl-4-thiazolemethanamino)-naphtahlimide (**4-HNFd**)

**4-HNFd** was synthesized by protocol B, yellow powder, yield 51% (the final step), obtain 0.0879 g. **^1^H NMR** (400 MHz, DMSO-*d*_6_) *δ* 11.95 (s, 1H), 8.58 (dd, *J* = 8.4, 1.3 Hz, 1H), 8.50 (dd, *J* = 7.3, 1.3 Hz, 1H), 8.39 (d, *J* = 8.3 Hz, 1H), 7.79 (dd, *J* = 8.4, 7.3 Hz, 1H), 7.19 (d, *J* = 8.4 Hz, 2H), 5.27 (d, *J* = 1.1 Hz, 2H), 1.30 (d, *J* = 7.0 Hz, 6H). **^13^C NMR** (101 MHz, DMSO) *δ* 177.25, 164.04, 163.31, 160.94, 151.85, 134.29, 131.85, 129.91, 129.59, 126.17, 122.96, 122.31, 113.56, 113.05, 110.49, 32.88, 23.37. **HRMS** (ESI): C_17_H_12_N_2_O_3_S [M+H]^-^, *m/z*: calculated: 351.0803, found: 351.0833.

(*E*)-2-benzylidene-5,6-dimethoxy-2,3-dihydro-1H-inden-1-one (**D1**)

Light yellow solid, 62.8% yield, 98.1% HPLC purity. **^1^HNMR** (400 MHz, CDCl_3_) *δ* 7.64 (d, *J* = 7.6 Hz, 2H, 2 × Ar-H), 7.59 (s, 1H, C=CH), 7.45 (t, *J* = 7.6 Hz, 2H, 2 × Ar-H), 7.38 (t, *J* = 7.2 Hz, 1H, Ar-H), 7.33 (s, 1H, Ar-H), 6.97 (s, 1H, Ar-H), 3.99 (s, 3H, OCH_3_), 3.95-3.94 (m, 5H, OCH_3_ + phCH_2_). **^13^C NMR** (100 MHz, CDCl_3_) *δ* 1931.1, 155.5, 149.7, 144.9, 135.6, 135.4, 132.4, 131.1, 130.5, 129.4, 128.9, 107.2, 105.1, 56.3, 56.2, 32.2. **HRMS** (ESI): C_18_H_16_O_3_ [M+H]^+^, *m/z*: 281.1.

(*E*)-2-(Furan-2-ylmethylene)-5,6-dimethoxy-2,3-dihydro-1H-inden-1-one (**D2**)

Light yellow solid, 50.3% yield, 97.6% HPLC purity. **^1^HNMR** (400 MHz, CDCl_3_) *δ* 7.60 (s, 1H, C=CH), 7.38 (s, 1H, Ar-H), 7.33 (s, 1H, Ar-H), 6.98 (s, 1H, Ar-H), 6.72 (d, *J* = 2.8 Hz, 1H, Ar-H), 6.55 (d, *J* = 1.6 Hz, 1H, Ar-H), 4.00 (s, 3H, OCH_3_), 3.97 (s, 2H, phCH_2_), 3.95 (s, 3H, OCH_3_). **^13^C NMR** (100 MHz, CDCl_3_) *δ* 192.8, 155.3, 152.4, 149.5, 145.1, 145.0, 133.3, 131.5, 118.7, 115.9, 112.5, 107.3, 105.0, 56.3, 56.2, 32.0. **HRMS** (ESI): C_16_H_14_O_4_ [M+H]^+^, *m/z*: 270.1.

(*E*)-2-([1,1'-Biphenyl]-4-ylmethylene)-5,6-dimethoxy-2,3-dihydro-1H-inden-1-one (**D3**)

Light yellow solid, 70.2% yield, 98.2% HPLC purity. **^1^HNMR** (400 MHz, CDCl_3_) *δ* 7.74 (d, *J* = 8.4 Hz, 2H, 2 × Ar-H), 7.69 (d, *J* = 8.4 Hz, 2H, 2 × Ar-H), 7.65-7.63 (m, 3H, 3 × Ar-H), 7.47 (t, *J* = 7.6 Hz, 1H, Ar-H), 7.37 (d, *J* = 10.8 Hz, 1H, Ar-H), 7.36 (s, 1H, Ar-H), 7.00 (s, 1H, Ar-H), 4.01 (s, 5H, OCH_3_ + phCH_2_), 3.96 (s, 3H, OCH_3_). **^13^C NMR** (100 MHz, CDCl_3_) *δ* 193.1, 155.5, 149.7, 144.9, 142.0, 140.2, 135.4, 134.6, 132.0, 131.1, 128.9, 127.9, 127.5, 127.1, 107.2, 105.1, 56.3, 56.2, 32.3. **HRMS** (ESI): C_24_H_20_O_3_ [M+H]^+^, *m/z*: 357.1.

(*E*)-5,6-Dimethoxy-2-(2-methylbenzylidene)-2,3-dihydro-1H-inden-1-one (**D4**)

Light yellow solid, 57.3% yield, 98.6% HPLC purity. **^1^H NMR** (400 MHz, CDCl_3_ ) *δ* 7.86 (s, 1H, C=CH), 7.63 (t, *J* = 2.4 Hz, 1H, Ar-H), 7.38 (s, 1H, Ar-H), 7.29-7.28 (m, 3H, 3 × Ar-H), 6.97 (s, 1H, Ar-H), 4.01 (s, 3H, OCH_3_), 3.98 (s, 3H, OCH_3_), 3.91 (s, 2H, phCH_2_), 2.50 (s, 3H, phCH_3_). **^13^C NMR** (100 MHz, CDCl_3_) *δ* 93.0, 155.4, 149.6, 145.3, 139.0, 136.2, 134.4, 131.2, 130.8, 130.1, 129.1, 128.5, 126.0, 107.2, 105.1, 56.3, 56.2, 31.8, 20.2. **HRMS** (ESI): C_19_H_18_O_3_ [M+H]^+^, *m/z*: 295.1.

(*E*)-5,6-dimethoxy-2-(3-methylbenzylidene)-2,3-dihydro-1H-inden-1-one (**D5**)

Light yellow solid, 70.2% yield, 97.3% HPLC purity. **^1^H NMR** (400 MHz, CDCl_3_ ) *δ* 7.54 (s, 1H, C=CH), 7.43 (d, *J* = 8.8 Hz, 2H, 2 × Ar-H), 7.34-7.27 (m, 2H, 2 × Ar-H), 7.18 (d, *J* = 7.6 Hz, 1H, Ar-H), 6.96 (s, 1H, Ar-H), 3.98 (s, 3H, OCH_3_), 3.93 (s, 3H, OCH_3_), 3.92 (s, 2H, CH_2_), 2.40 (s, 3H, phCH_3_). **^13^C NMR** (100 MHz, CDCl_3_) *δ* 193.1, 149.6, 144.9, 138.5, 135.2, 132.5, 131.3, 131.1, 130.2, 128.7, 127.6, 107.2, 105.0, 56.3, 56.1, 32.2, 31.5. **HRMS** (ESI): C_19_H_18_O_3_ [M+H]^+^, *m/z*: 294.35.

(*E*)-5,6-Dimethoxy-2-(4-methoxybenzylidene)-2,3-dihydro-1H-inden-1-one (**D6**)

Light yellow solid, 67.2% yield, 98.1% HPLC purity. **^1^HNMR** (400 MHz, CDCl_3_ ) *δ* 7.62 (d, *J* = 8.8 Hz, 2H, 2 × Ar-H), 7.57 (s, 1H, C=CH), 7.35 (s, 1H, Ar-H), 6.99 (s, 2H, 2 × Ar-H), 6.97 (s, 1H, Ar-H), 4.00 (s, 3H, OCH_3_), 3.95 (s, 3H, OCH_3_), 3.94 (s, 2H, phCH_2_), 3.87 (s, 3H, OCH_3_). **^13^C NMR** (100 MHz, CDCl_3_) *δ* 193.3, 160.6, 155.2, 149.6, 144.7, 133.2, 132.3, 131.3, 128.4, 114.4, 107.2, 105.1, 56.3, 55.4, 32.2. **HRMS** (ESI): C_19_H_18_O_4_ [M+H]^+^, *m/z*: 311.1.

(*E*)-1-(2-methoxyphenyl)-3-(naphthalen-2-yl)prop-2-en-1-one (**D7**)

Yellow oil. 89.0% yield. **^1^H NMR** (500 MHz, Chloroform-*d*) *δ* 7.96 (s, 1H), 7.88 – 7.81 (m, 3H), 7.78 (d, *J* = 15.8 Hz, 1H), 7.74 (dd, *J* = 8.6, 1.8 Hz, 1H), 7.65 (dd, *J* = 7.6, 1.8 Hz, 1H), 7.54 – 7.44 (m, 4H), 7.10 – 6.97 (m, 2H), 3.92 (s, 3H). **^13^C NMR** (125 MHz, Chloroform-*d*) *δ* 193.05, 158.14, 143.43, 134.28, 133.39, 132.88, 132.68, 130.47, 130.37, 129.39, 128.65, 128.60, 127.80, 127.30, 127.24, 126.70, 123.78, 120.79, 111.69, 55.82, 53.46. **HRMS** (ESI): C_20_H_16_O_2_ [M+H]^+^ *m/z*: calculated: 289.1229, found: 289.1221.

(*E*)-1-(2-hydroxyphenyl)-3-(naphthalen-2-yl)prop-2-en-1-one (**D8**)

Yellow solid. 66.1% yield.**^1^H NMR** (800 MHz, Chloroform-*d*) *δ* 12.89 (s, 1H), 8.15 – 8.09 (m, 2H), 8.02 (dd, *J* = 7.9, 1.7 Hz, 1H), 7.96 – 7.90 (m, 2H), 7.90 – 7.87 (m, 1H), 7.85 (dd, *J* = 8.4, 1.8 Hz, 1H), 7.81 (d, *J* = 15.3 Hz, 1H), 7.61 – 7.52 (m, 3H), 7.08 (dd, *J* = 8.2, 1.1 Hz, 1H), 7.03 – 6.96 (m, 1H). **^13^C NMR** (200 MHz, Chloroform-*d*) *δ* 145.61, 136.45, 131.17, 129.71, 128.88, 128.79, 127.88, 127.66, 126.93, 123.69, 120.25, 118.90, 118.70. **HRMS** (ESI): C_19_H_14_O_2_ [M+H]^+^ *m/z*: calculated: 275.1073, found: 275.1065.

(*E*)-1-(6-hydroxy-2,3,4-trimethoxyphenyl)-3-(naphthalen-2-yl)prop-2-en-1-one (**D9**)

Yellow solid. 89.4% yield.**^1^H NMR** (500 MHz, Chloroform-*d*) *δ* 13.71 (s, 1H), 8.07 (d, *J* = 15.6 Hz, 1H), 8.04 – 7.98 (m, 2H), 7.92 – 7.82 (m, 3H), 7.80 (dd, *J* = 8.6, 1.7 Hz, 1H), 7.55 – 7.50 (m, 2H), 6.32 (s, 1H), 3.96 (s, 3H), 3.92 (s, 3H), 3.86 (s, 3H).**^13^C NMR** (125 MHz, Chloroform-*d*) *δ* 192.89, 162.76, 160.22, 155.04, 143.40, 135.36, 134.31, 133.43, 132.90, 130.57, 128.75, 128.66, 127.81, 127.30, 126.75, 126.71, 123.77, 108.83, 96.64, 62.02, 61.36, 56.15. **HRMS** (ESI): C_22_H_20_O_5_ [M+H]^+^ *m/z*: calculated: 365.1390, found: 365.1381.

(*E*)-1-(4-aminophenyl)-3-(naphthalen-2-yl)prop-2-en-1-one (**D10**)

Yellow solid. 92.5% yield.**^1^H NMR** (500 MHz, Chloroform-*d*) *δ* 8.08 – 7.95 (m, 4H), 7.93 – 7.85 (m, 3H), 7.83 (dd, *J* = 8.6, 1.7 Hz, 1H), 7.69 (d, *J* = 15.6 Hz, 1H), 7.57 – 7.51 (m, 2H), 6.75 (d, *J* = 8.5 Hz, 2H).**^13^C NMR** (125 MHz, Chloroform-*d*) *δ* 188.06, 151.07, 143.25, 134.21, 133.44, 132.84, 131.13, 130.17, 128.70, 128.60, 127.79, 127.13, 126.67, 123.81, 122.19, 113.98, 32.77, 31.94, 30.05, 29.72, 29.38, 22.71, 14.14, 1.03. **HRMS** (ESI): C_19_H_15_NO [M+H]^+^ *m/z*: calculated: 274.1223, found: 274.1228.

(*E*)-1-(4-methoxyphenyl)-3-(quinolin-6-yl)prop-2-en-1-one (**D11**)

Yellow solid. 79.1% yield.**^1^H NMR** (500 MHz, DMSO-*d*_6_) *δ* 9.17 (dd, *J* = 4.9, 1.6 Hz, 1H), 8.88 (dd, *J* = 8.5, 1.6 Hz, 1H), 8.65 – 8.57 (m, 2H), 8.29 (d, *J* = 8.9 Hz, 1H), 8.23 – 8.12 (m, 3H), 7.93 (dd, *J* = 8.4, 4.9 Hz, 1H), 7.88 (d, *J* = 15.6 Hz, 1H), 7.14 – 7.06 (m, 2H), 3.87 (s, 3H). **^13^C NMR** (125 MHz, DMSO-*d*_6_) *δ* 187.16, 163.45, 147.65, 142.89, 142.01, 141.16, 134.86, 131.36, 131.09, 130.17, 130.15, 128.49, 124.77, 124.31, 122.64, 114.12, 55.62. **HRMS** (ESI): C_19_H_15_NO_2_ [M+H]^+^ *m/z*: calculated: 290.1182, found: 290.1176.

(*E*)-1-(6-hydroxy-2,3,4-trimethoxyphenyl)-3-(quinolin-6-yl)prop-2-en-1-one (**D12**)

Yellow solid. 73.5% yield.**^1^H NMR** (500 MHz, Chloroform-*d*) *δ* 13.45 (s, 1H), 9.12 – 8.94 (m, 2H), 8.88 (s, 1H), 8.35 (d, *J* = 7.9 Hz, 1H), 8.22 (s, 1H), 8.15 (d, *J* = 15.6 Hz, 1H), 7.92 (d, *J* = 15.6 Hz, 2H), 6.32 (s, 1H), 3.98 (s, 3H), 3.93 (s, 3H), 3.86 (s, 3H). **^13^C NMR** (125 MHz, Chloroform-*d*) *δ* 191.87, 163.04, 161.07, 154.85, 145.42, 143.22, 138.68, 137.81, 135.38, 132.73, 131.54, 129.37, 128.57, 123.33, 121.91, 108.59, 96.64, 62.02, 61.39, 56.27. **HRMS** (ESI): C_21_H_19_NO_5_ [M+H]^+^ *m/z*: calculated: 366.1342, found: 366.1331.

(E)-3-(isoquinolin-6-yl)-1-(4-(pyrrolidin-1-yl)phenyl)prop-2-en-1-one (**D13**)

Yellow solid. 87.9% yield. **^1^H NMR** (600 MHz, Chloroform-*d*) *δ* 9.26 (s, 1H), 8.56 (d, *J* = 5.7 Hz, 1H), 8.04 (d, *J* = 8.7 Hz, 2H), 8.02 – 7.98 (m, 2H), 7.94 – 7.89 (m, 2H), 7.77 (d, *J* = 15.6 Hz, 1H), 7.70 (d, *J* = 5.8 Hz, 1H), 6.59 (d, *J* = 8.7 Hz, 2H), 3.44 – 3.38 (m, 4H), 2.08 – 2.04 (m, 4H). **^13^C NMR** (151 MHz, CDCl_3_) *δ* 187.03, 151.99, 151.24, 143.48, 140.91, 137.67, 136.14, 131.17, 128.76, 128.25, 127.65, 125.62, 125.25, 124.85, 120.89, 111.04, 47.64, 25.46. **HRMS** (ESI): C_22_H_20_N_2_O [M+H]^+^ *m/z*: 329.1.

(*E*)-1-(3-methoxyphenyl)-3-(quinoxalin-6-yl)prop-2-en-1-one (**D14**)

White solid. 87.9% yield.**^1^H NMR** (500 MHz, Chloroform-*d*) *δ* 8.88 (dd, *J* = 13.1, 1.8 Hz, 2H), 8.33 (d, *J* = 1.9 Hz, 1H), 8.16 (d, *J* = 8.8 Hz, 1H), 8.08 (dd, *J* = 8.8, 2.0 Hz, 1H), 8.01 (d, *J* = 15.6 Hz, 1H), 7.73 (d, *J* = 15.7 Hz, 1H), 7.66 (dt, *J* = 7.6, 1.2 Hz, 1H), 7.59 (dd, *J* = 2.7, 1.5 Hz, 1H), 7.46 (t, *J* = 7.9 Hz, 1H), 7.18 (ddd, *J* = 8.2, 2.7, 0.9 Hz, 1H), 3.91 (s, 3H). **^13^C NMR** (125 MHz, Chloroform-*d*) *δ* 189.73, 160.04, 145.85, 145.62, 144.01, 143.19, 142.96, 139.25, 136.68, 130.55, 130.27, 129.74, 128.50, 124.58, 121.16, 119.76, 112.89, 55.56. **HRMS** (ESI): C_18_H_14_N_2_O_2_ [M+H]^+^ *m/z*: calculated: 291.1134, found: 291.1132.

(*E*)-1-(2-hydroxyphenyl)-3-(quinoxalin-6-yl)prop-2-en-1-one (**D15**)

Yellow solid. 72.3% yield.**^1^H NMR** (500 MHz, Chloroform-*d*) *δ* 12.71 (s, 1H), 8.90 (dd, *J* = 12.8, 1.8 Hz, 2H), 8.36 (d, *J* = 1.9 Hz, 1H), 8.18 (d, *J* = 8.8 Hz, 1H), 8.15 – 8.06 (m, 2H), 7.98 (dd, *J* = 8.0, 1.7 Hz, 1H), 7.87 (d, *J* = 15.5 Hz, 1H), 7.55 (ddd, *J* = 8.6, 7.2, 1.6 Hz, 1H), 7.07 (dd, *J* = 8.4, 1.2 Hz, 1H), 7.00 (ddd, *J* = 8.2, 7.1, 1.2 Hz, 1H). **^13^C NMR** (125 MHz, Chloroform-*d*) *δ* 193.32, 163.74, 145.95, 145.79, 144.14, 143.58, 143.17, 136.82, 136.34, 130.89, 130.39, 129.71, 128.49, 122.89, 122.83, 119.94, 119.10, 119.06, 118.81, 118.71, 1.03. **HRMS** (ESI): C_17_H_12_N_2_O_2_ [M+H]^+^ *m/z*: calculated: 277.0978, found: 277.0971.

(*E*)-3-(2-chloroquinolin-6-yl)-1-(2-hydroxy-6-methoxyphenyl)prop-2-en-1-one (**D16**)

Yellow solid. 91.1% yield.**^1^H NMR** (500 MHz, Chloroform-*d*) *δ* 13.10 (s, 1H), 8.18 (d, *J* = 8.5 Hz, 1H), 8.10 (d, *J* = 8.8 Hz, 1H), 8.06 (dd, *J* = 8.9, 1.8 Hz, 1H), 8.04 – 7.92 (m, 3H), 7.47 (d, *J* = 8.5 Hz, 1H), 7.42 (t, *J* = 8.3 Hz, 1H), 6.67 (d, *J* = 8.4 Hz, 1H), 6.49 (d, *J* = 8.2 Hz, 1H), 4.02 (s, 3H). **^13^C NMR** (125 MHz, Chloroform-*d*) *δ* 194.13, 164.98, 161.02, 151.66, 148.48, 141.22, 139.31, 136.29, 134.17, 129.52, 129.30, 129.20, 128.56, 127.00, 123.22, 111.94, 111.09, 101.61, 56.10. **HRMS** (ESI): C_19_H_14_ClNO_3_ [M+H]^+^ *m/z*: calculated: 340.0741, found: 340.0738.

(*E*)-3-(2-chloroquinolin-6-yl)-1-(2-hydroxy-4-methoxyphenyl)prop-2-en-1-one (**D17**)

Yellow solid. 72.6% yield. **^1^H NMR** (600 MHz, Chloroform-*d*) *δ* 13.35 (s, 1H), 8.14 (d, *J* = 8.5 Hz, 1H), 8.06 (s, 2H), 8.03 – 7.97 (m, 2H), 7.85 (d, *J* = 8.9 Hz, 1H), 7.70 (d, *J* = 15.5 Hz, 1H), 7.44 (d, *J* = 8.5 Hz, 1H), 6.53 – 6.46 (m, 2H), 3.86 (s, 3H). **^13^C NMR** (150 MHz, Chloroform-*d*) *δ* 191.38, 166.87, 166.50, 151.95, 148.72, 142.80, 139.21, 133.54, 131.26, 129.52, 129.41, 128.62, 126.97, 123.33, 122.03, 114.06, 108.02, 101.14, 55.69. **HRMS** (ESI): C_19_H_14_ClNO_3_ [M+H]^+^ *m/z*: calculated: 340.0741, found: 340.0736.

(*E*)-1-(2-hydroxy-4,6-dimethoxyphenyl)-3-(quinoxalin-6-yl)prop-2-en-1-one (**D18**)

Yellow oil. 74.0% yield.**^1^H NMR** (500 MHz, Chloroform-*d*) *δ* 14.19 (s, 1H), 8.88 (dd, *J* = 13.5, 1.9 Hz, 2H), 8.31 (d, *J* = 1.9 Hz, 1H), 8.15 (d, *J* = 8.8 Hz, 1H), 8.10 (d, *J* = 15.6 Hz, 1H), 8.03 (dd, *J* = 8.8, 2.0 Hz, 1H), 7.95 (d, *J* = 15.6 Hz, 1H), 6.14 (d, *J* = 2.4 Hz, 1H), 6.00 (d, *J* = 2.4 Hz, 1H), 3.97 (s, 3H), 3.86 (s, 3H). **^13^C NMR** (125 MHz, Chloroform-*d*) *δ* 192.12, 168.57, 166.64, 162.57, 145.53, 145.25, 143.71, 143.05, 140.22, 137.61, 130.45, 130.02, 129.73, 128.99, 106.37, 93.87, 91.44, 56.04, 55.69. **HRMS** (ESI): C_19_H_16_N_2_O_4_ [M+H]^+^ *m/z*: calculated: 337.1189, found: 337.1185.

**3. Supplementary Tables and Figures**

**Table S1. The properties and shortages of the previously reported CYP3A4 fluorescent substrates.**

| **Name** | **λ_ex_/λ_em_ (nm)** | **Specificity** | **Application scope*** | **Shortages** | **Ref.** |
| --- | --- | --- | --- | --- | --- |
| 7-methoxyquinoline | 410/505 | untested | *in vitro* (total activity of CYPs in microsomes) | Short detection wavelength; cannot distinguish CYP isoform enzymes; unavailable *in vivo* | ^[6]^ |
| 7-Benzyloxyquinoline (BQ) | 410/538 | only detect its metabolism in 9 CYP isoform enzymes (1A2, 2A6, 2B6, 2C8, 2C9, 2C19, 2D6, 2E1, 3A4) | *in vitro* (rCYPs, microsomes) | short detection wavelength; unavailable *in vivo* | ^[7]^ |
| 7-benzyloxy-4-trifluoromethylcoumarin (BFC) | 410/538 | CYP3A4 non-specific substrate |  |  |  |
| 7-benzyloxyresorufin (BzRes) | 530/590 | CYP3A4 non-specific substrate | *in vitro* (rCYPs, microsomes) | low cell-membrane permeability; unavailable *in vivo* | ^[8]^ |
| 7-benzyloxy-4-trifluoromethylcoumarin (BFC) | 410/538 | CYP3A4 non-specific substrate |  | short detection wavelength; unavailable *in vivo* |  |
| 7-benzyloxyquinoline (BQ) | 410/538 | only detect its metabolism in 9 CYP isoform enzymes (1A2, 2A6, 2B6, 2C8, 2C9, 2C19, 2D6, 2E1, 3A4) |  | short detection wavelength; unavailable *in vivo* |  |
| dibenzylfluorescein (DBF) | 485/530 | CYP2C8, CYP3A4, CYP3A5 |  | short detection wavelength; unavailable *in vivo* |  |
| **NEN** | 450/558 | CYP3A4 specific substrate | *in vitro* (rCYP3A4, microsomes) | low cell-membrane permeability; unavailable *in vivo* | ^[9]^ |
| **F8** | 450/555 | CYP3A4 specific substrate | *in vitro* (rCYP3A4, microsomes, living cells, and tissues) and *in vivo* (*i.v.*) | Oral unavailable | ^[10]^ |

* rCYPs means recombinant CYP enzymes; *i.v.*, Means intravenous injection.

**Table S2.** Data splitting for model development and evaluation.

| **Data set** | **Data class** | **Training** | **Validation** | **Test** | **total** |
| --- | --- | --- | --- | --- | --- |
| CYP3A4 | inactivate | 9196 | 3066 | 3065 | 15327 |
|  | activate | 7031 | 2344 | 2343 | 11718 |
|  | total | 16227 | 5410 | 5626 | 27045 |

**Table S3.** Performance of AFP-3A4 on the training, validation, and test set.

|  | **Loss** | **AUC-ROC** | **AUPR** | **Precision** | **Recall** |
| --- | --- | --- | --- | --- | --- |
| **Training** | 0.3887 | 0.9025 | 0.8685 | 0.7667 | 0.8503 |
| **Validation** | 0.4236 | 0.8878 | 0.8544 | 0.7654 | 0.8315 |
| **Test** | 0.4176 | 0.8900 | 0.8558 | 0.7638 | 0.8161 |

**Table S4.** Kinetic parameters of **NFa, NFb, NFd** 4-hydroxylation determined in HLMs. Data are expressed as mean ± SD (n = 3).

| **Enzyme source** | **Substrate** | **Metabolites** | ***V*_max_*^a^*** | ***K*_m_ (μM)** | ***CL*_int_*^b^*** |
| --- | --- | --- | --- | --- | --- |
| HLMs | **NFa** | **4-HNFa** | 1.75 ± 0.09 | 13.78 ± 1.71 | 127.00 |
|  | **NFb** | **4-HNFb** | 1.04 ± 0.05 | 9.51 ± 1.20 | 109.36 |
|  | **NFd** | **4-HNFd** | 0.46 ± 0.01 | 3.44 ± 0.20 | 133.72 |

^a)^ The unit of *V*_max_ is nmol/min/nmol hCYP3A4.

^b)^ The unit of *V*_max_/*K*_m_ is μL/min/nmol hCYP3A4.

**Table S5.** The molar extinction coefficient and fluorescence quantum yield of **NFa** and **4-HNFa**.

|  | **Molar extinction coefficient**  **ε (M^-1^ cm^-1^)** | **Fluorescence quantum yield**  ***Φ*_f_** |
| --- | --- | --- |
| **NFa** | 44300 | -- |
| **4-HNFa** | 25300 | 0.129 |

**Table S6.** The lipophilicity of **NEN**, **F8,** and **NFa** (Prediction by ADMETlab 2.0).

|  | **logS** | **logP** | **logD** |
| --- | --- | --- | --- |
| **NEN** | -4.473 | 2.812 | 2.431 |
| **F8** | -6.448 | 3.902 | 3.507 |
| **NFa** | -4.577 | 2.582 | 2.308 |

logS: Log of the aqueous solubility. Optimal: -4~0.5 log mol/L

logP: Log of the octanol/water partition coefficient. Optimal: 0~3

logD: logP at physiological pH 7.4. Optimal: 1~3

**Table S7.** Inhibitory effects of CYP3A4 positive inhibitors (ritonavir and ketoconazole) on hCYP3A4 in HLMs and CYP3A4. Data are expressed as mean ± SD (n = 3). λ_ex_ = 450 nm

| **Enzyme sources** | **Ritonavir (nM)** | **Ketoconazole (nM)** |
| --- | --- | --- |
| HLMs | 4.02 ± 0.35 | 9.42 ± 1.07 |
| CYP3A4 | 4.87 ± 0.37 | 8.61 ± 0.66 |

**Table S8.** The inhibitory potentials of 93 natural products and their derivatives against hCYP3A4 using **NFa** as the fluorogenic substrate in HLMs. Data are expressed as mean ± SD (n = 3).

| **No.** | **Compound name** | **CAS/Structure** | **Residual activity (%) ^*^** |
| --- | --- | --- | --- |
| 1 | KET | 65277-42-1 | 1.91 |
| 2 | RTV | 155213-67-5 | 1.50 |
| 3 | Kaempferol | 520-18-3 | 67.86 |
| 4 | Echinatin | 34221-41-5 | 47.57 |
| 5 | Oleanolic acid | 508-02-1 | 70.22 |
| 6 | Licochalcone A | 58749-22-7 | 39.20 |
| 7 | Licochalcone C | 144506-14-9 | 59.04 |
| 8 | Glycyrrhetinic acid | 471-53-4 | 66.89 |
| 9 | Formononetin | 485-72-3 | 98.92 |
| 10 | Magnolol | 528-43-8 | 105.98 |
| 11 | Ursolic acid | 77-52-1 | 99.33 |
| 12 | Arctigenin | 7770-78-7 | 102.87 |
| 13 | Pectolinarigenin | 520-12-7 | 98.03 |
| 14 | Pimpinellin | 131-12-4 | 102.72 |
| 15 | Resveratrol | 501-36-0 | 72.13 |
| 16 | Sophocarpine | 6483-15-4 | 94.11 |
| 17 | Oxysophocarpine | 26904-64-3 | 96.07 |
| 18 | Aconitine | 302-27-2 | 94.81 |
| 19 | Hypaconitine | 6900-87-4 | 94.90 |
| 20 | Lappaconitine | 32854-75-4 | 94.46 |
| 21 | Strychnine | 57-24-9 | 96.35 |
| 22 | Hyoscyamine | 101-31-5 | 97.96 |
| 23 | Scopolamine | 51-34-3 | 98.83 |
| 24 | Fuziline | 80665-72-1 | 102.53 |
| 25 | Songorine | 509-24-0 | 104.37 |
| 26 | Oxynitidine | 509-24-0 | 89.98 |
| 27 | Chelerythrine | 34316-15-9 | 89.61 |
| 28 | Harmine | 442-51-3 | 101.14 |
| 29 | Camptothecin | 7689-03-4 | 93.84 |
| 30 | Lycorine | 2188-68-3 | 92.09 |
| 31 | Cytisine | 485-35-8 | 94.14 |
| 32 | Dauricine | 524-17-4 | 92.80 |
| 33 | Daurisoline | 70553-76-3 | 91.85 |
| 34 | Tetrandrine | 518-34-3 | 95.25 |
| 35 | Stachydrine | 515-24-2 | 94.28 |
| 36 | Epiberberine | 6873-09-2 | 89.82 |
| 37 | Coptisine | 3486-66-6 | 77.91 |
| 38 | Protopanaxadiol | 7755-01-3 | 69.66 |
| 39 | Protopanaxatriol | 1453-93-6 | 43.85 |
| 40 | Silybin | 22888-70-6 | 89.00 |
| 41 | Phloretin | 60-82-2 | 97.31 |
| 42 | Daidzein | 486-66-8 | 97.43 |
| 43 | Anhydroicaritin | 38226-86-7 | 87.82 |
| 44 | Aromadendrin | 480-20-6 | 91.70 |
| 45 | Isobavachalcone | 20784-50-3 | 54.20 |
| 46 | Norwogonin | 4443-09-8 | 85.31 |
| 47 | Icaritin | 118525-40-9 | 90.12 |
| 48 | Tectorigenin | 548-77-6 | 95.51 |
| 49 | Genkwanin | 437-64-9 | 58.20 |
| 50 | Liquiritigenin | 578-86-9 | 90.60 |
| 51 | Morin | 480-16-0 | 82.26 |
| 52 | Calycosin | 20575-57-9 | 99.33 |
| 53 | Bavachalcone | 28448-85-3 | 90.43 |
| 54 | Neobavaisoflavone | 41060-15-5 | 87.90 |
| 55 | Bavachin | 19879-32-4 | 71.61 |
| 56 | Sanggenone C | 80651-76-9 | 92.64 |
| 57 | Herbacetin | 527-95-7 | 91.06 |
| 58 | Hesperetin | 520-33-2 | 96.85 |
| 59 | Glycitein | 40957-83-3 | 104.35 |
| 60 | Lysionotin | 152743-19-6 | 96.88 |
| 61 | Xanthohumol | 6754-58-1 | 59.86 |
| 62 | Nobiletin | 478-01-3 | 101.53 |
| 63 | Farrerol | 24211-30-1 | 73.49 |
| 64 | Acacetin | 480-44-4 | 49.92 |
| 65 | Alpinetin | 1090-65-9 | 95.70 |
| 66 | Galangin | 548-83-4 | 93.98 |
| 67 | Protocatechuic acid | 99-50-3 | 109.40 |
| 68 | Anthraquinone | 84-65-1 | 94.28 |
| 69 | 1-Hydroxy anthraquinone | 129-43-1 | 90.54 |
| 70 | 2-Ethyl anthraquinone | 84-51-5 | 79.76 |
| 71 | 2-Methyl anthraquinone | 84-54-8 | 83.58 |
| 72 | 1,5- Dihydroxyanthraquinone | 117-12-4 | 99.84 |
| 73 | Anthraquinone-2-carboxylic acid | 117-78-2 | 98.05 |
| 74 | 1-Aminoanthraquinone | 82-45-1 | 62.61 |
| 75 | 2,6-Dibromoanthraquinone | 633-70-5 | 85.49 |
| 76 | 1,8-Dichloroanthraquinone | 82-43-9 | 85.47 |
| 77 | 2,6-Dihydroxyanthraquinone | 84-60-6 | 88.82 |
| 78 | **D1** | **** | 88.05 |
| 79 | **D2** | **** | 102.09 |
| 80 | **D3** | **** | 75.93 |
| 81 | **D4** | **** | 58.11 |
| 82 | **D5** | **** | 72.34 |
| 83 | **D6** | **** | 67.76 |
| 84 | **D7** | **** | 95.65 |
| 85 | **D8** | **** | 88.04 |
| 86 | **D9** | **** | 85.00 |
| 87 | **D10** | **** | 62.72 |
| 88 | **D11** | **** | 57.66 |
| 89 | **D12** | **** | 70.04 |
| 90 | **D13** | **** | 4.08 |
| 91 | **D14** | **** | 63.85 |
| 92 | **D15** | **** | 64.49 |
| 93 | **D16** | **** | 100.04 |
| 94 | **D17** | **** | 83.59 |
| 95 | **D18** | **** | 54.03 |

^*^The final concentration of each tested inhibitor is 1 μM, while the residual activities were expressed as the mean of duplicates.

**Table S9.** The pharmacokinetic parameters of **NFa** when **NFa** was administered orally (20 mg/kg, *i.g.*, n = 6) and intravenously (2 mg/kg, *i.v.*, n = 6).

| **Mode of administration** | ***T_max_* (h)** | ***C_max_* (ng/mL)** | ***AUC*_(0-inf)_ (ng/mL*h)** | ***t*_1/2_ (h)** | **Oral bioavailability (%)** |
| --- | --- | --- | --- | --- | --- |
| i.g. | 1.17 ± 0.41 | 249.88 ± 90.01 | 669.74 ± 231.93 | 1.55 ± 0.61 | 15.27 |
| i.v. | 0.083 ± 0.00 | 912.65 ± 115.73 | 438.55 ± 40.72 | 1.24 ± 0.39 |  |


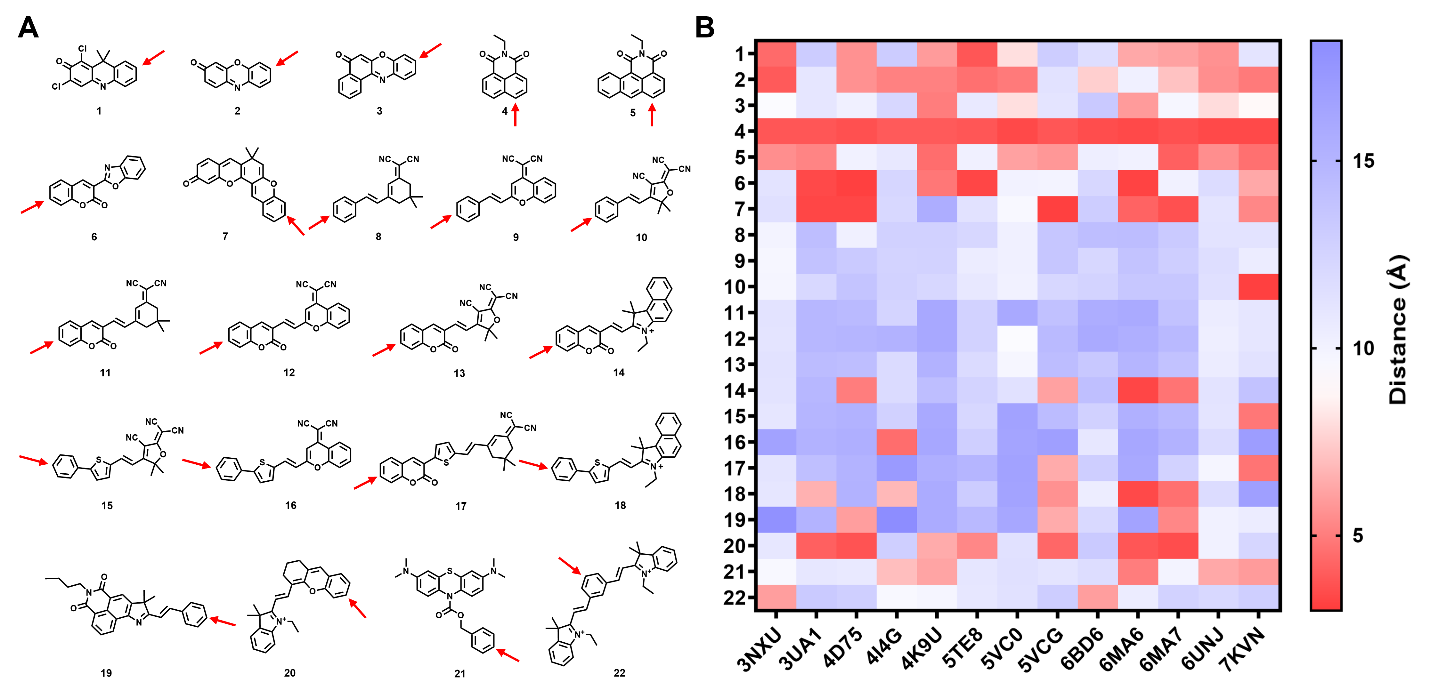


**Figure S1.** (a) Chemical structures of 22 fluorophores. (b) Ensemble docking prediction of catalytic distances (the first conformation) between the Fe atom of hCYP3A4 and the hydroxylation site (the atom indicated by the red arrow) of 22 fluorophores.


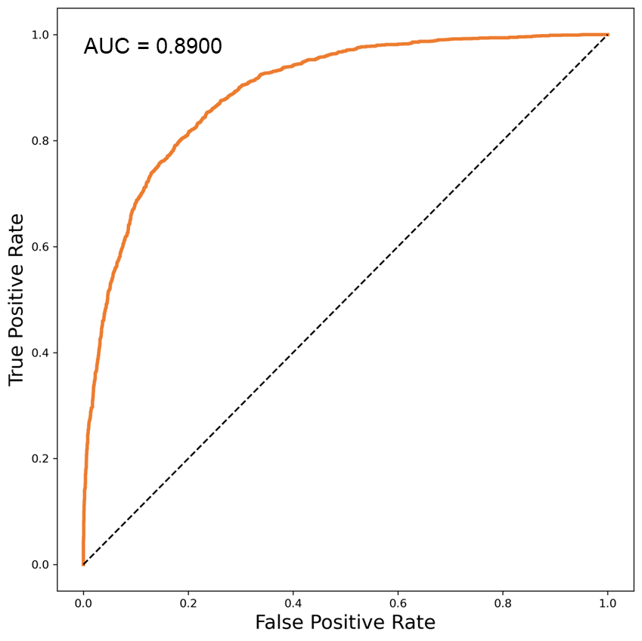


**Figure S2.** The AUC-ROC curve of test results for AFP-3A4.


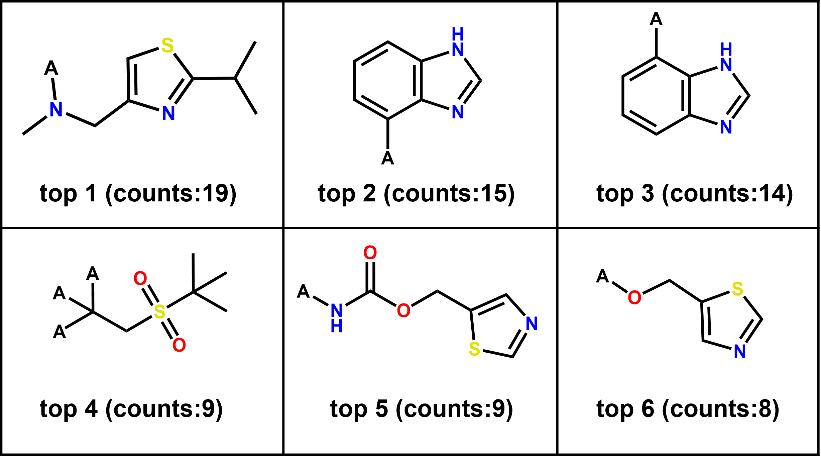


**Figure S3.** The top 6 fragments with significant differences from the CYP3A4 inhibitor database.


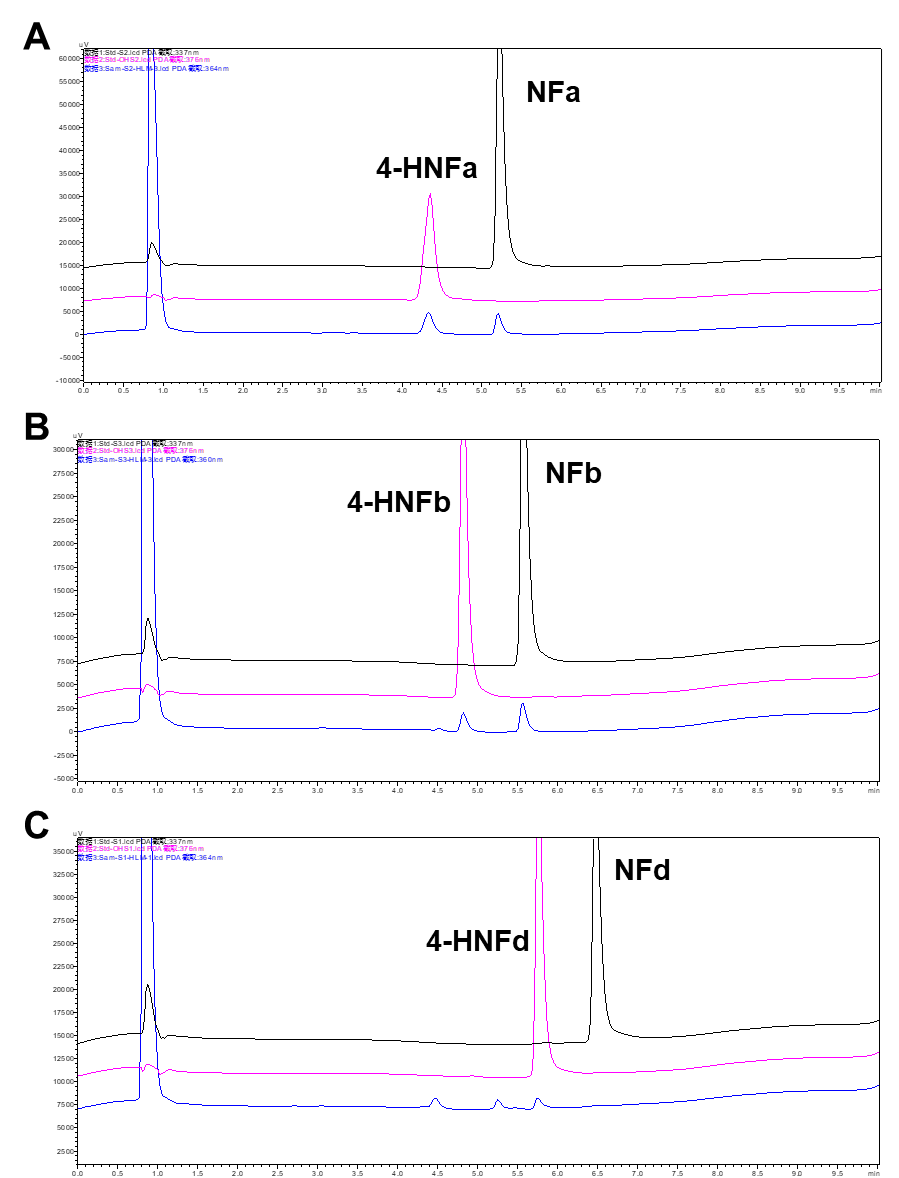


**Figure S4.** The LC-UV spectra of **NFs,** **4-HNFs** and their oxidative metabolites.


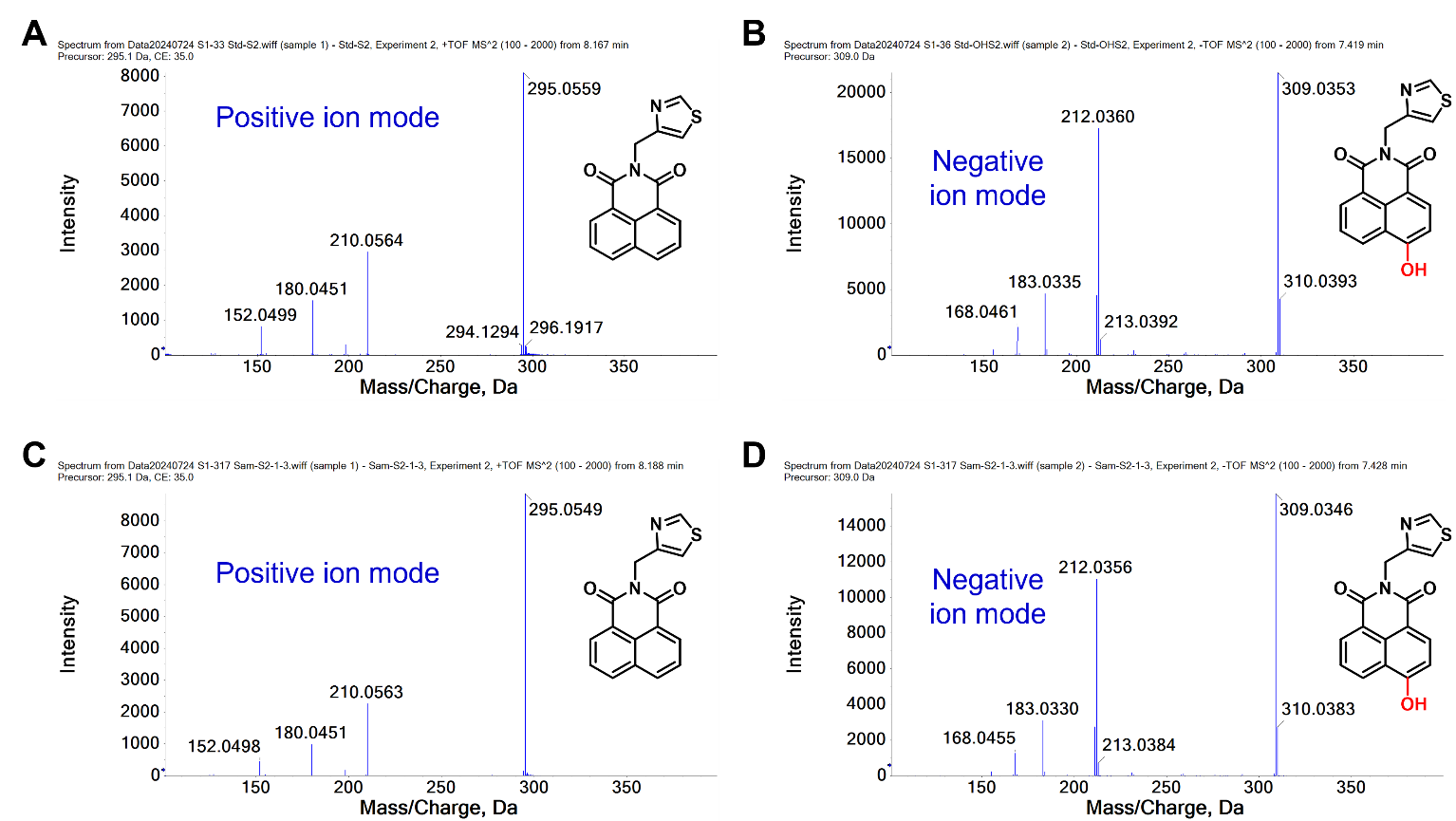


**Figure S5.** The MS/MS spectrum of **NFa** (A, positive), **4-HNFa** (B, negative) standards. The MS/MS spectrum of **NFa** (C, positive; D, negative) was incubated in HLMs for 30 min.

**
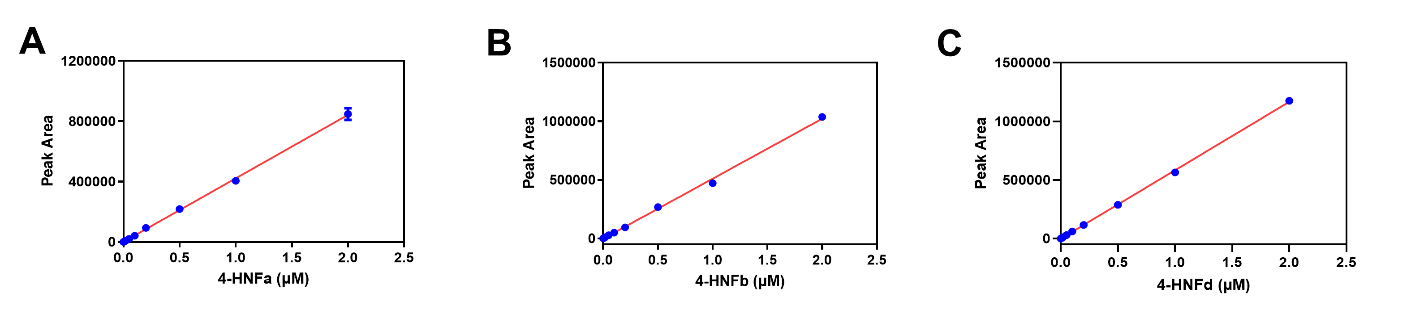
**

**Figure S6.** The concentration-dependent curves of **4-HNFa**, **4-HNFb**, **4-HNFd** in PBS: acetonitrile (1:1) using liquid chromatography with fluorescence detection (LC-FD). Data are expressed as mean ± SD (n = 3).


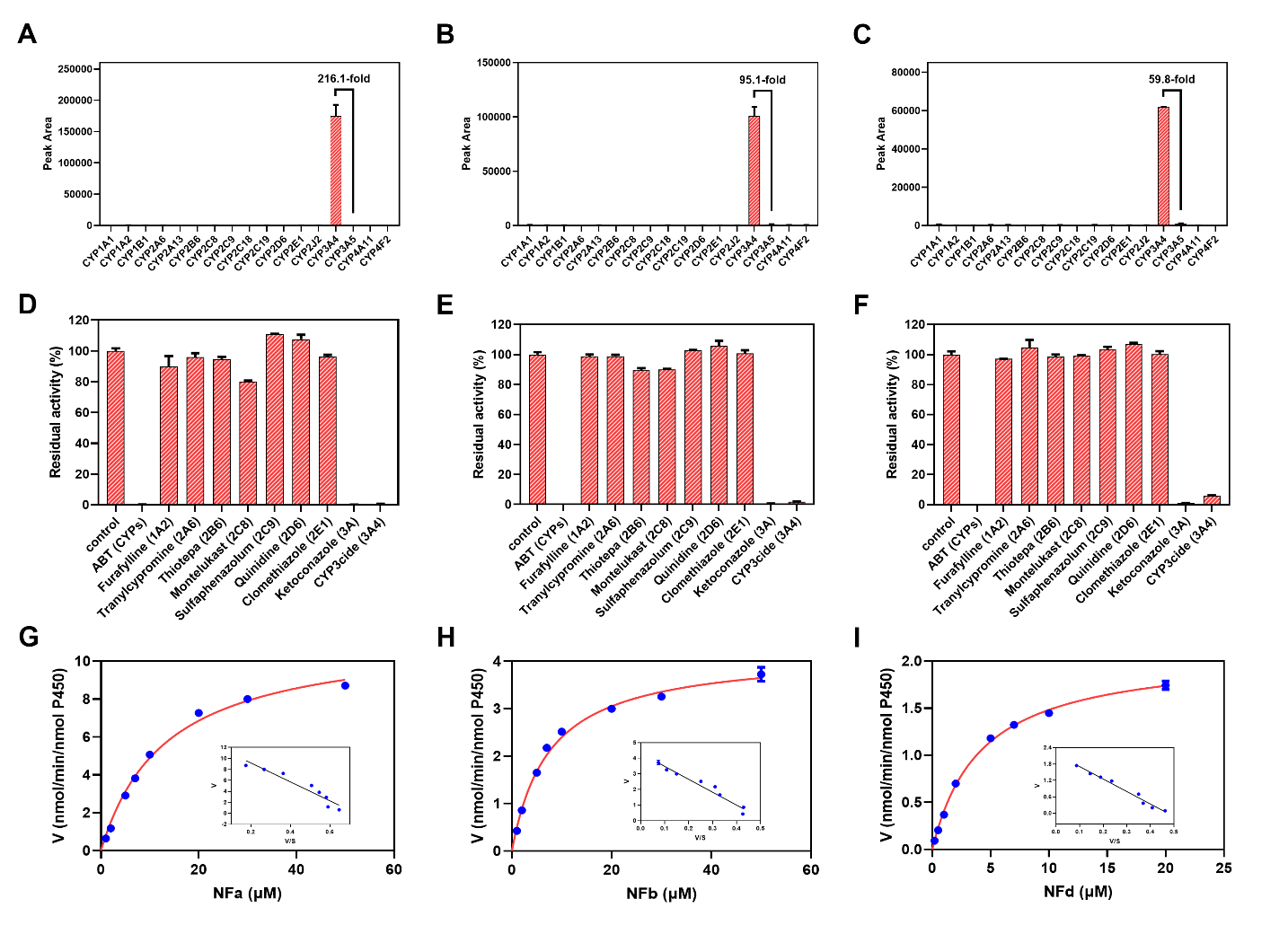


**Figure S7. NFa** (A)/**NFb** (B)/**NFd** (C) was concomitantly incubated with each tested human CYP enzyme for 30 min under physiological conditions (37 °C, pH 7.4). The inhibitory effects of CYP inhibitors on **NFa** (D)/**NFb** (E)/**NFd** (F) 4-hydroxylation in HLMs. Michaelis–Menten plots of **NFa** (G)/**NFb** (H)/**NFd** (I) 4-hydroxylation in hCYP3A4. The corresponding Eadie–Hofstee plot is shown as an inset. Data are expressed as mean ± SD (n = 3).


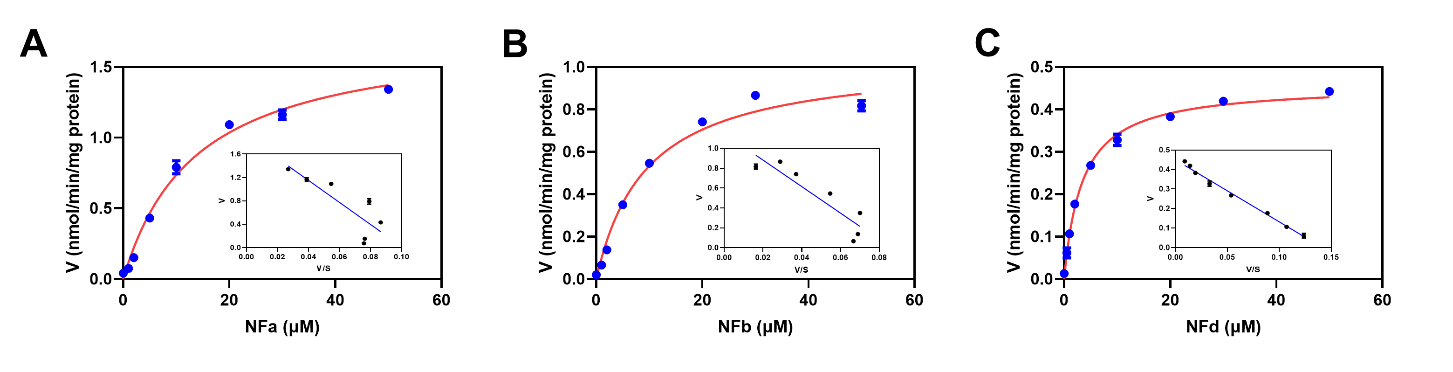


**Figure S8.** Michaelis–Menten plot of **NFa** 4-hydroxylation in HLMs. The corresponding Eadie–Hofstee plot is shown as an inset. Data are expressed as mean ± SD (n = 3).

**Chemical of standard**

^1^H NMR (600 MHz, DMSO-*d*_6_) *δ* 11.91 (s, 1H), 8.99 (d, *J* = 2.0 Hz, 1H), 8.57 (dd, *J* = 8.3, 1.3 Hz, 1H), 8.49 (dd, *J* = 7.3, 1.2 Hz, 1H), 8.38 (d, *J* = 8.2 Hz, 1H), 7.79 (dd, *J* = 8.3, 7.2 Hz, 1H), 7.48 (dd, *J* = 2.1, 1.1 Hz, 1H), 7.18 (d, *J* = 8.2 Hz, 1H), 5.36 (s, 2H). ^13^C NMR (151 MHz, DMSO-*d*_6_) *δ* 164.04, 163.31, 160.92, 154.35, 153.34, 134.26, 131.82, 129.89, 129.59, 126.16, 122.96, 122.30, 115.34, 113.06, 110.51.

**Chemical preparation in liver microsomes:**

^1^H NMR (600 MHz, DMSO-*d*_6_) *δ* 11.92 (s, 1H), 8.99 (d, *J* = 2.0 Hz, 1H), 8.58 (dd, *J* = 8.3, 1.2 Hz, 1H), 8.50 (dd, *J* = 7.3, 1.2 Hz, 1H), 8.38 (d, *J* = 8.2 Hz, 1H), 7.79 (dd, *J* = 8.3, 7.3 Hz, 1H), 7.48 (dd, *J* = 2.1, 1.1 Hz, 1H), 7.19 (d, *J* = 8.2 Hz, 1H), 5.37 (s, 2H). ^13^C NMR (151 MHz, DMSO-*d*_6_) *δ* 164.06, 163.33, 160.93, 154.36, 153.34, 134.28, 131.84, 129.90, 129.60, 126.18, 122.97, 122.32, 115.34, 113.07, 110.52.


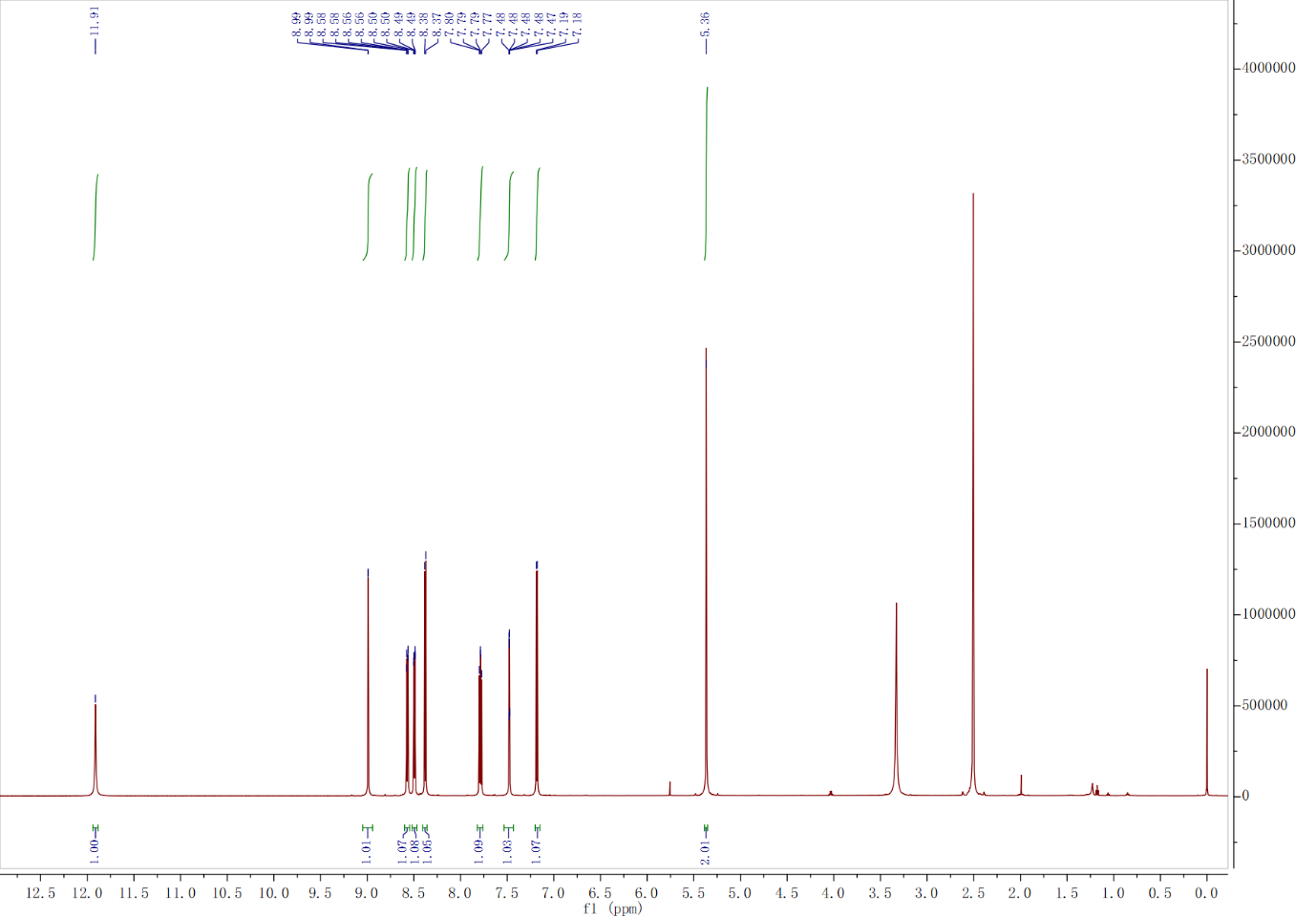


**Figure S9.** ^1^H NMR spectra of **4-HNFa** standard in DMSO-d6.


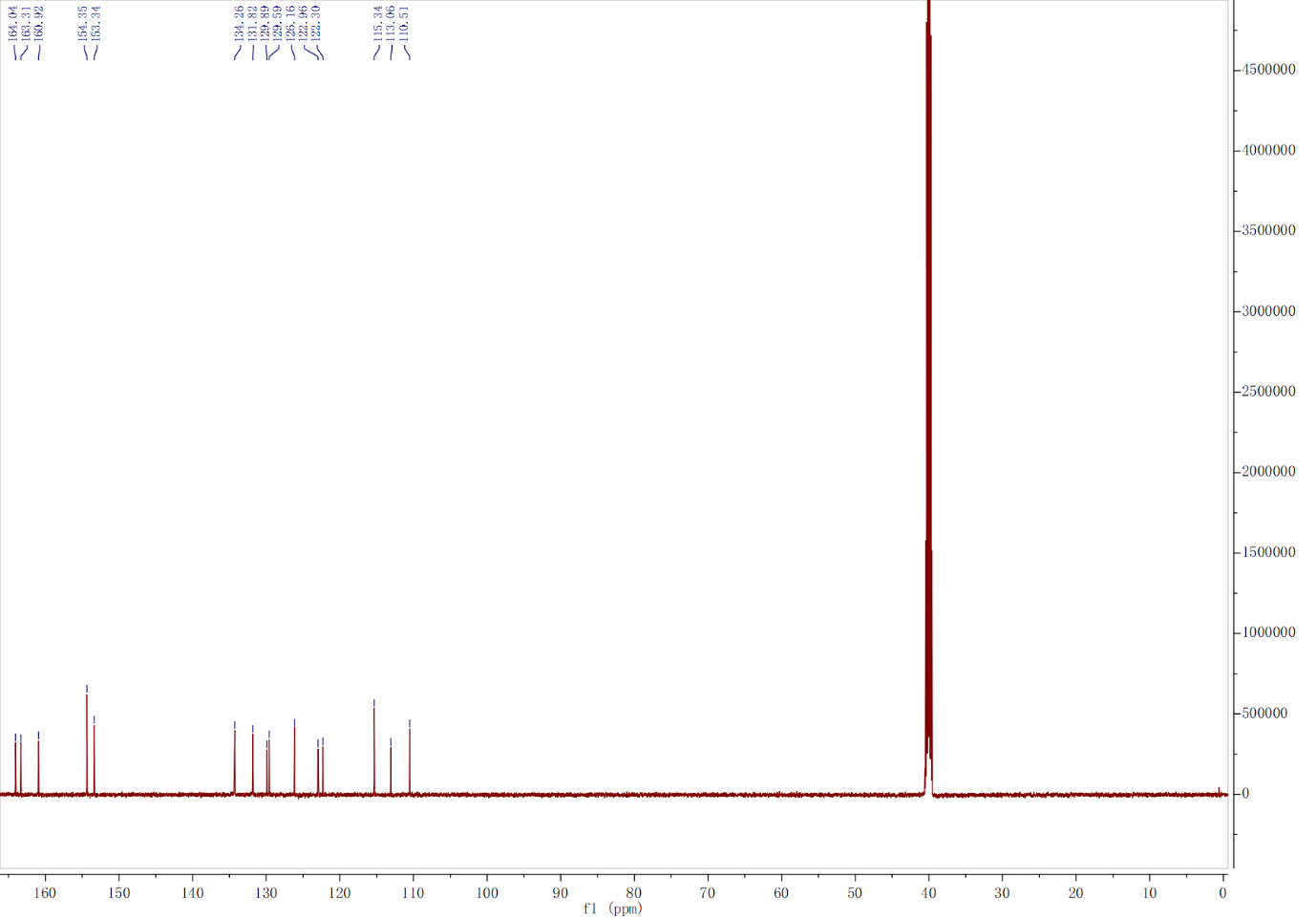


**Figure S10.** ^13^C NMR spectra of **4-HNFa** standard in DMSO-d6.


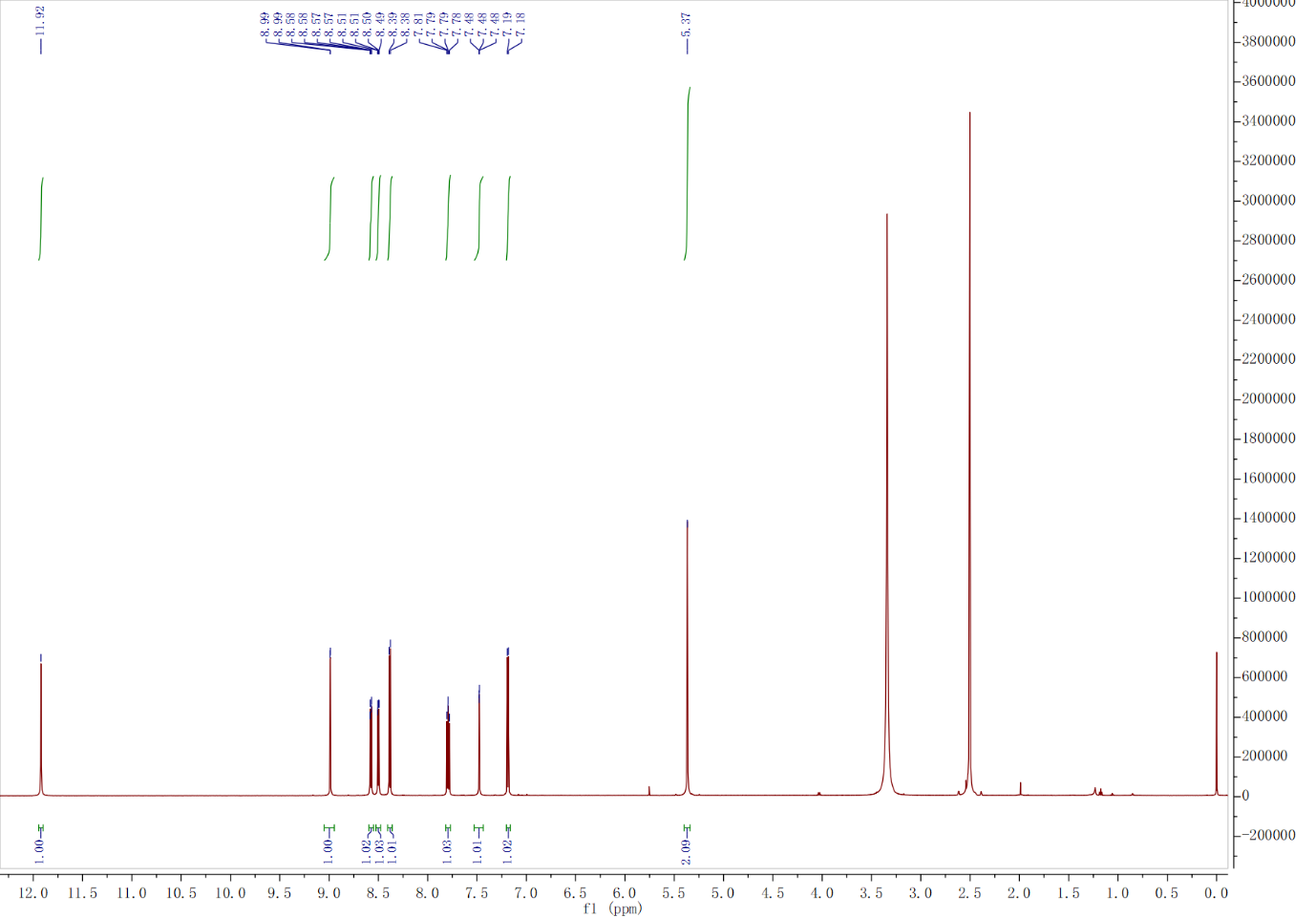


**Figure S11.** ^1^H NMR spectra of **4-HNFa** sample preparation in DMSO-d6.


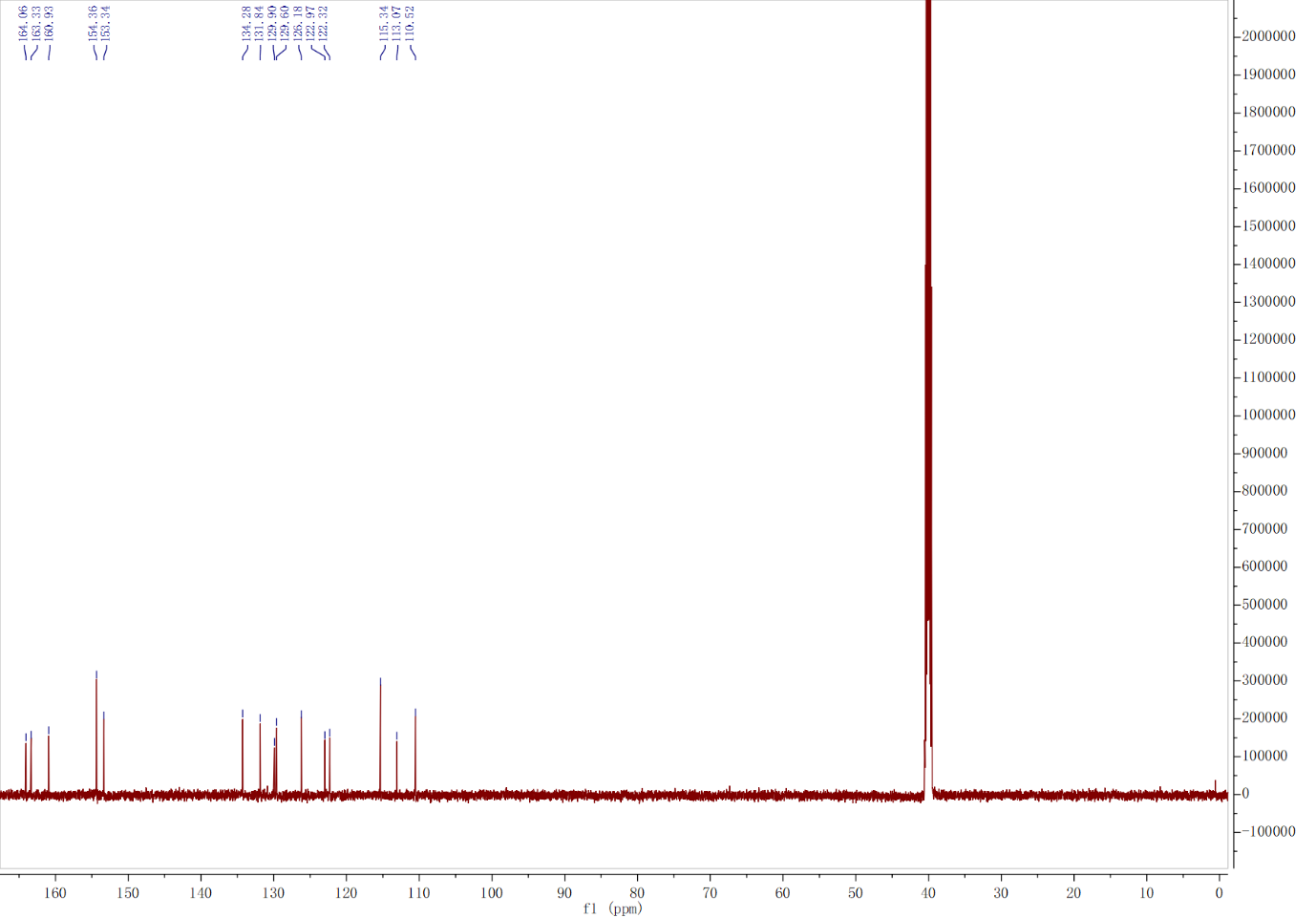


**Figure S12.** ^13^C NMR spectra of **4-HNFa** sample preparation in DMSO-d6.


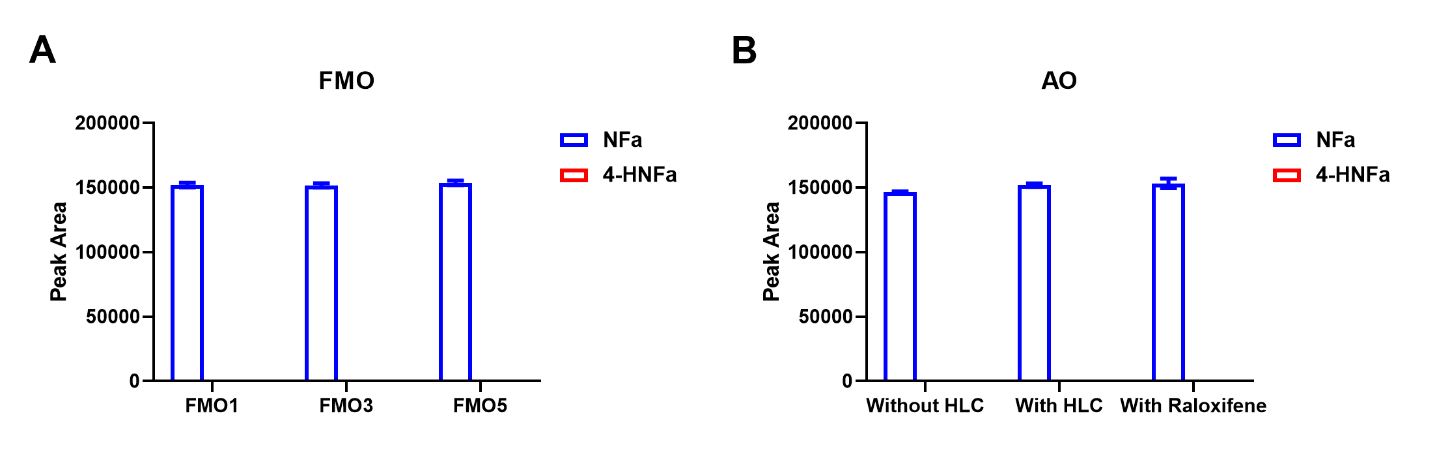


**Figure S13.** (A) The oxidative metabolism of **NFa** in three FMO enzymes (FMO1, FMO3, FMO5) in the presence of NADPH. (B) The oxidative metabolism of **NFa** in human liver cytosol (HLC) without NADPH. Data are expressed as mean ± SD (n = 3).


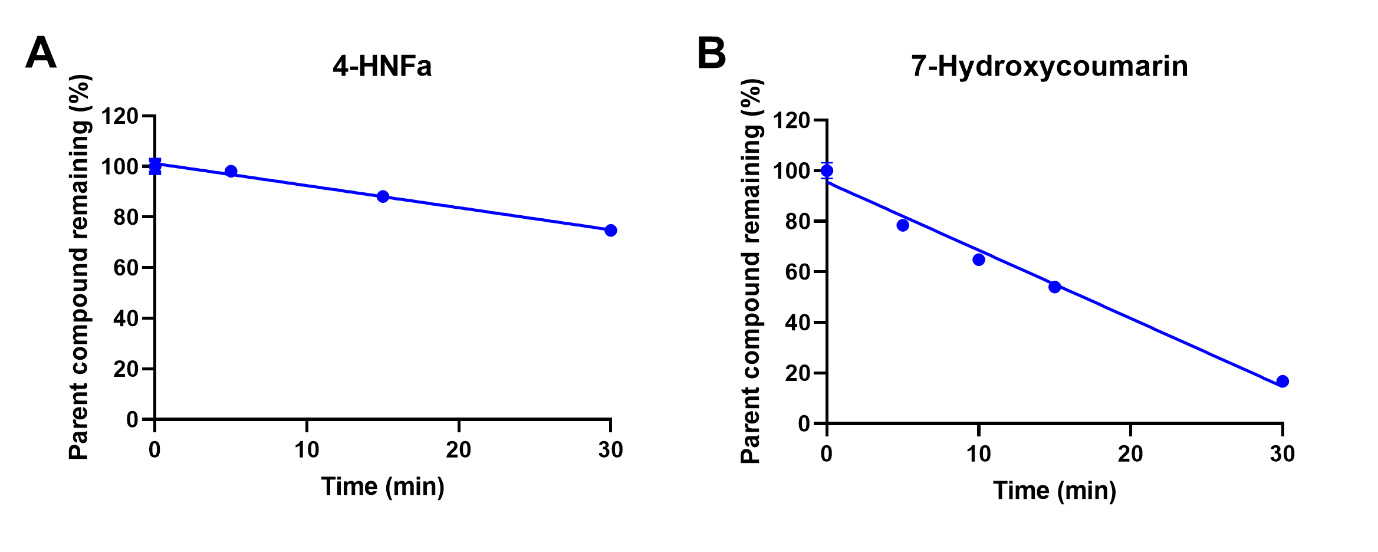


**Figure S14.** The metabolic half-life of **4-HNFa** and 7-hydroxycoumarin in HLMs in the presence of UDPGA. Data are expressed as mean ± SD (n = 3).


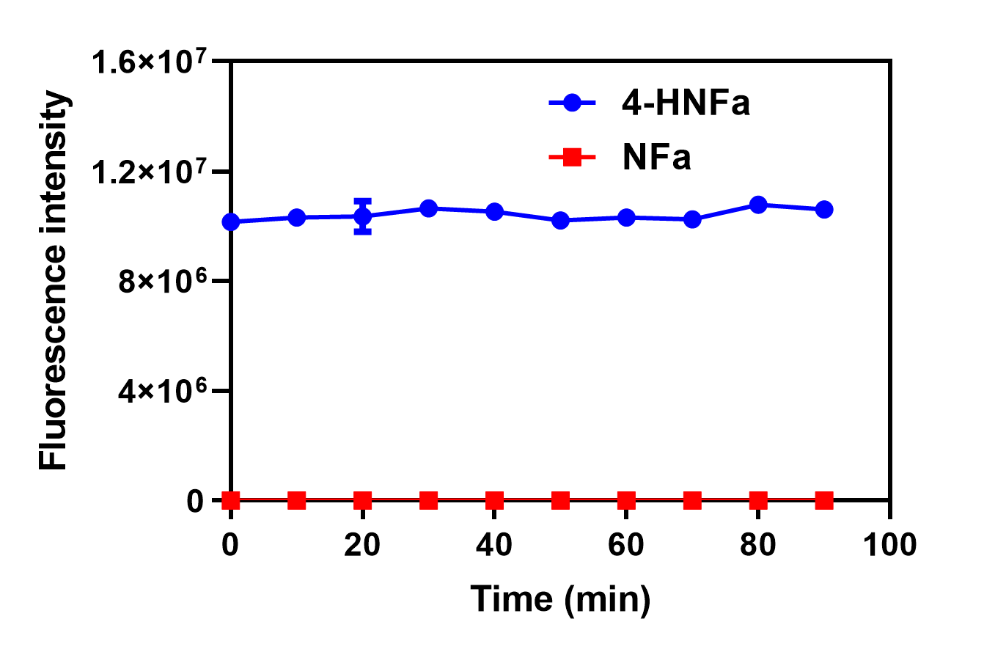


**Figure S15.** The change of fluorescence intensity of **NFa** and **4-HNFa** after continuous illumination at 450 nm (excitation wavelength) using a fluorescence microplate reader at different times. Data are expressed as mean ± SD (n = 3). *λ*_ex_ = 450 nm.


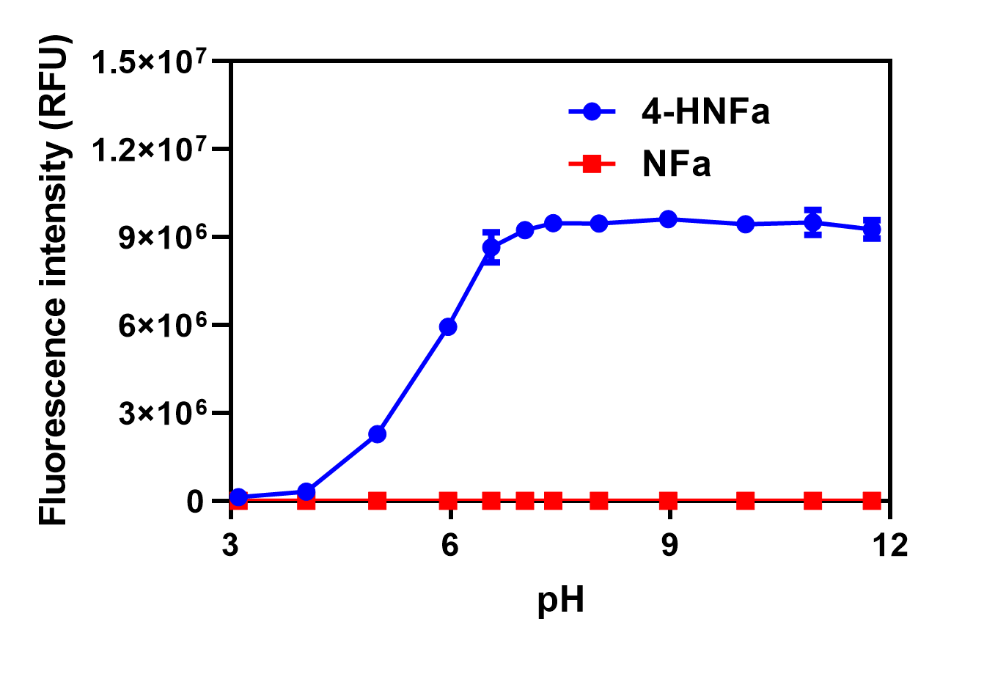


**Figure S16.** Effects of pH on the fluorescence intensities of **NFa** and its hydroxylated metabolite **4-HNFa** (2 μM). The different pH values are adjusted by HCl and NaOH. Data are expressed as mean ± SD (n = 3). *λ*_ex_ = 450 nm.


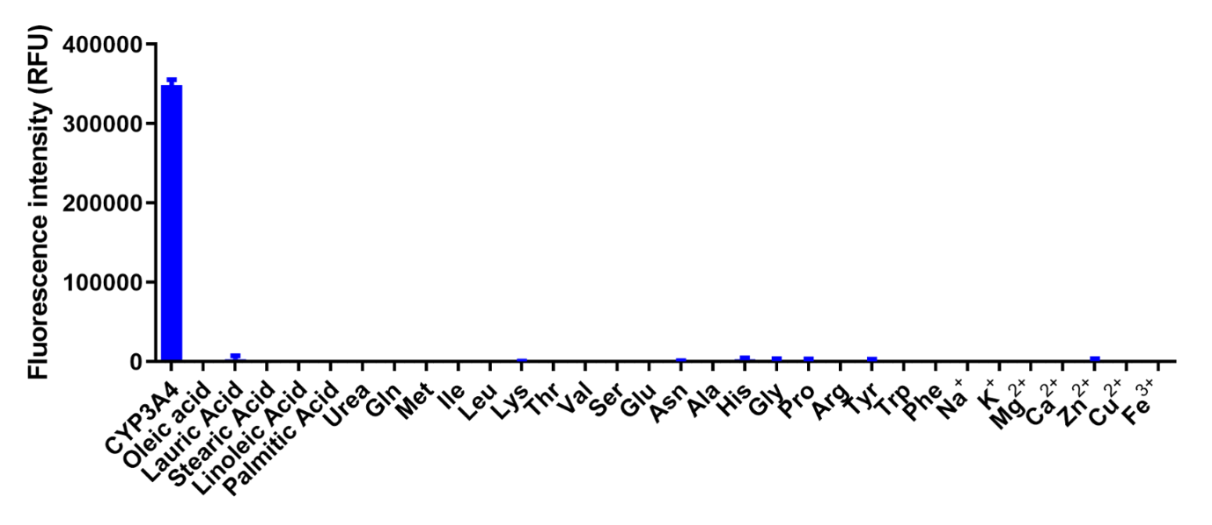


**Figure S17.** Fluorescence intensities of **NFa** (10 μM) upon addition of various analytes at 37 ℃ for 30 min. Data are expressed as mean ± SD (n = 3). λ_ex_ = 450 nm.


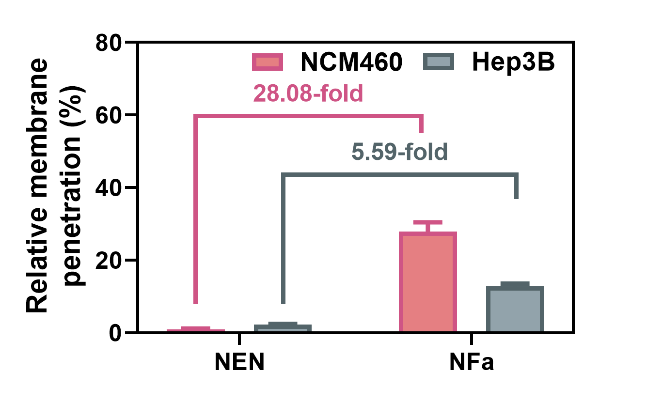


**Figure S18.** The relative cell-membrane permeabilities of **NEN** and **NFa** in NCM460 and Hep3B cell lines, respectively. Data are expressed as mean ± SD (n = 3).


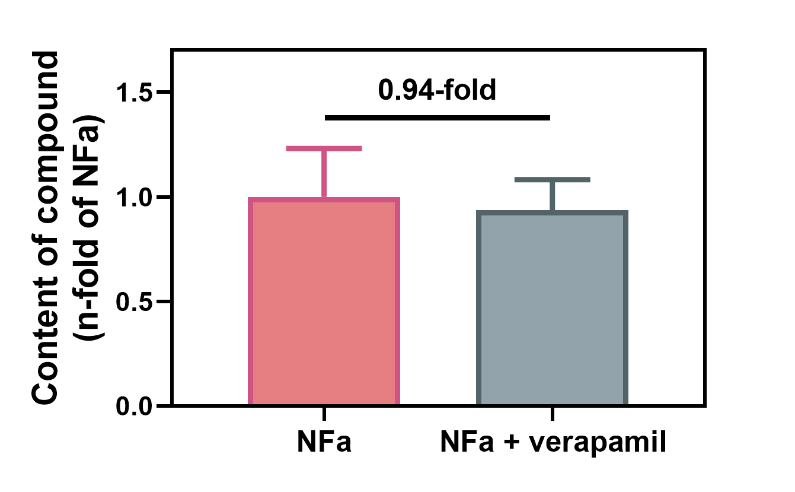


**Figure S19. NFa** is a non-substrate of P-gp. Data are expressed as mean ± SD (n = 3).


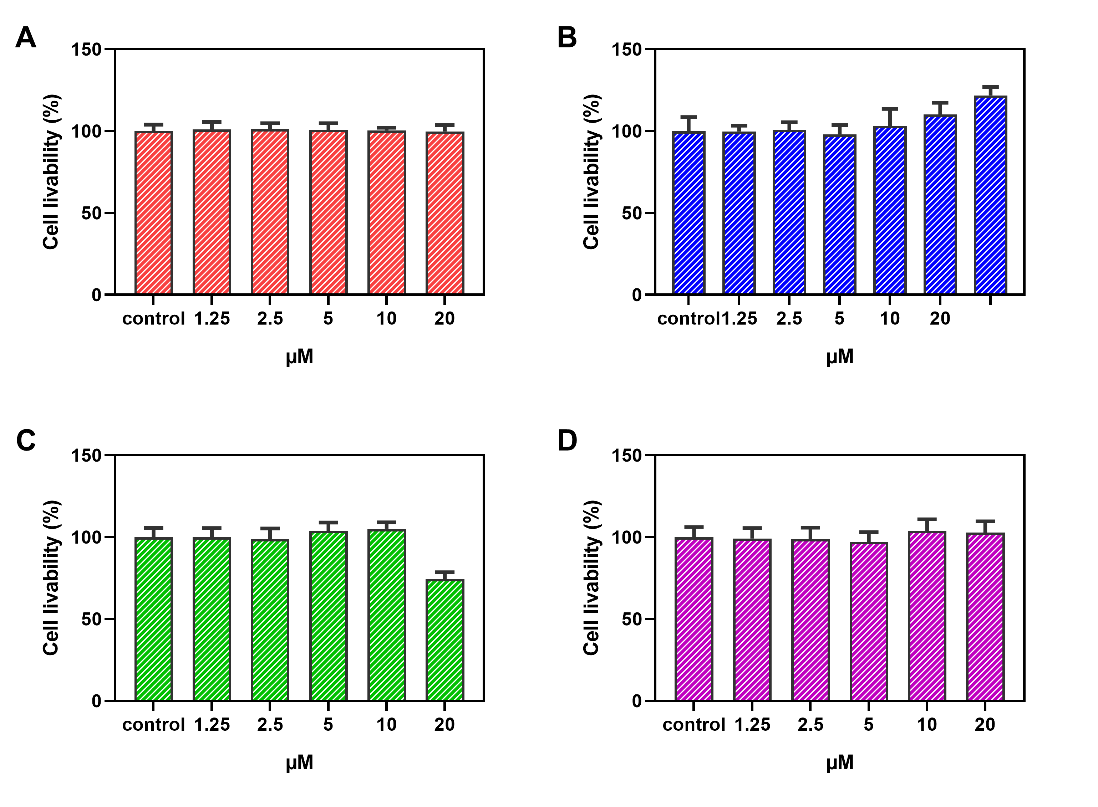


**Figure S20.** Cytotoxicity assays of **NFa** (A), **4-HNFa** (B), ketoconazole (C), and ritonavir (D) in Hep3B cell line. Data are expressed as mean ± SD (n = 3).

**
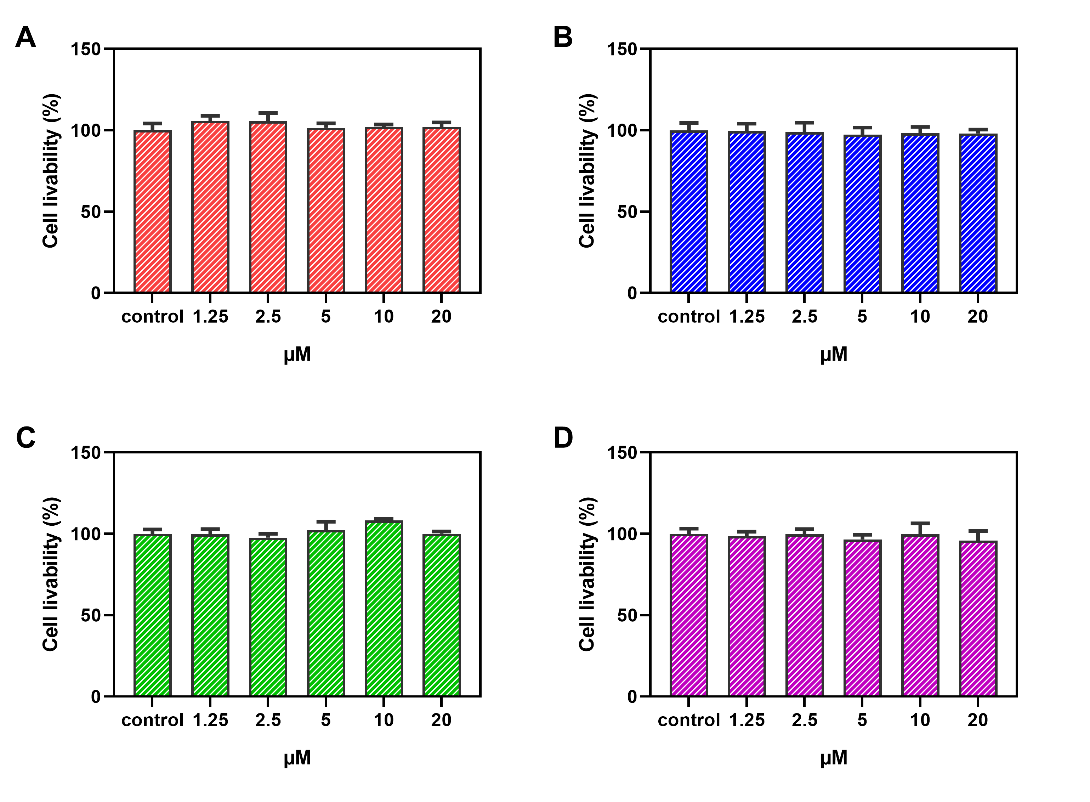
**

**Figure S21.** Cytotoxicity assays of **NFa** (A), **4-HNFa** (B), ketoconazole (C), and ritonavir (D) in MCF-7 cell line. Data are expressed as mean ± SD (n = 3).

**
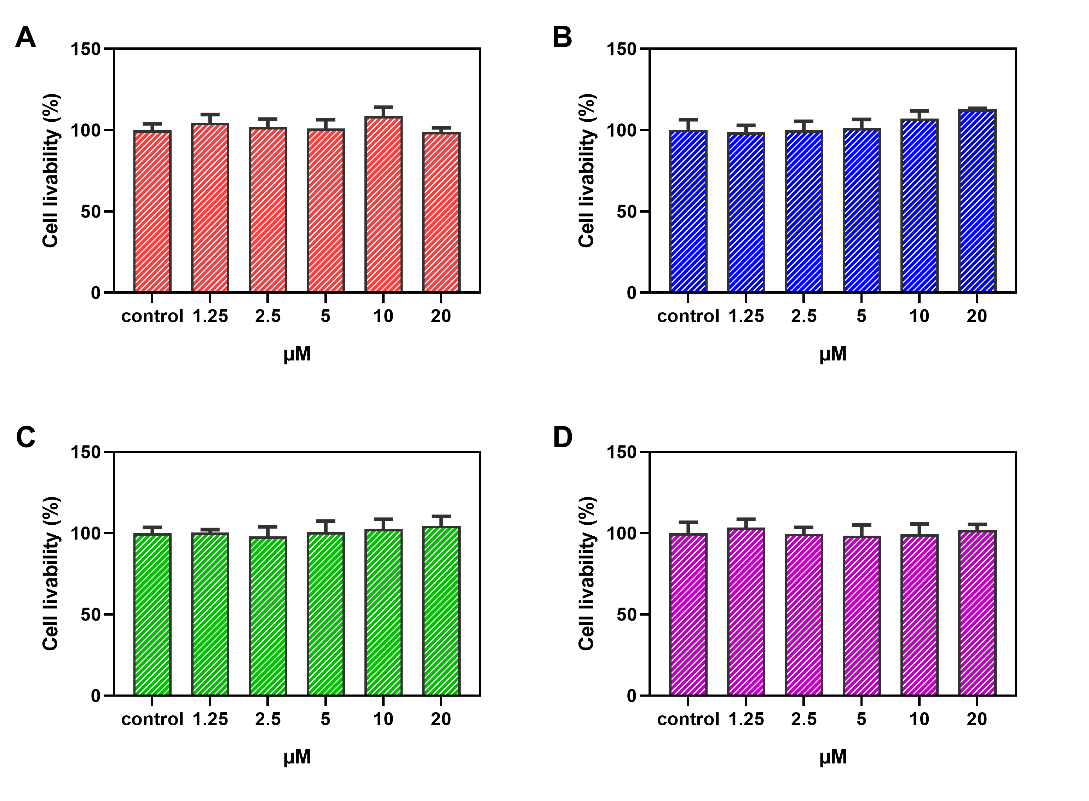
**

**Figure S22.** Cytotoxicity assays of **NFa** (A), **4-HNFa** (B), ketoconazole (C), and ritonavir (D) in U87 cell line. Data are expressed as mean ± SD (n = 3).


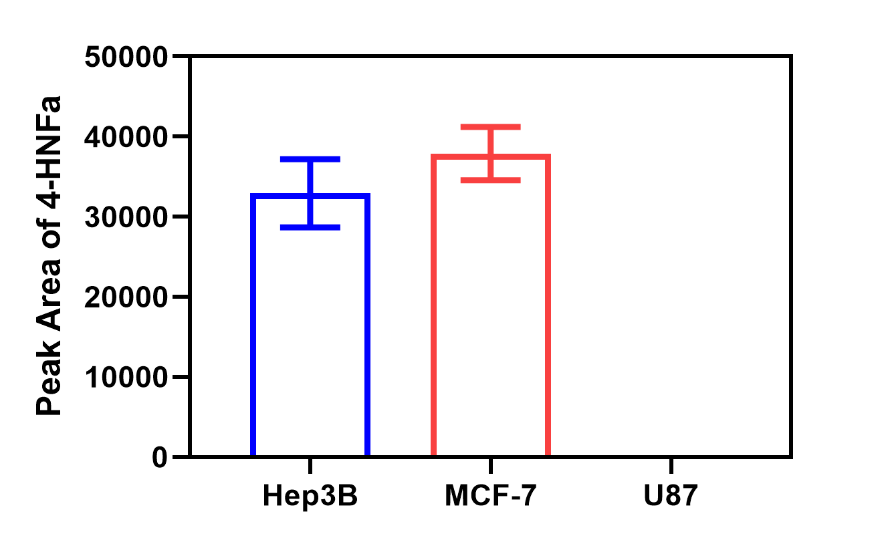


**Figure S23.** The catalytic activity of CYP3A4 in Hep3B, MCF-7, and U87 cells. Data are expressed as mean ± SD (n = 3).


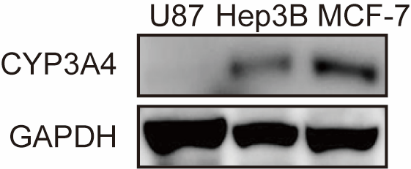


**Figure S24.** The protein expression of CYP3A4 in Hep3B, MCF-7, and U87 cells.

**
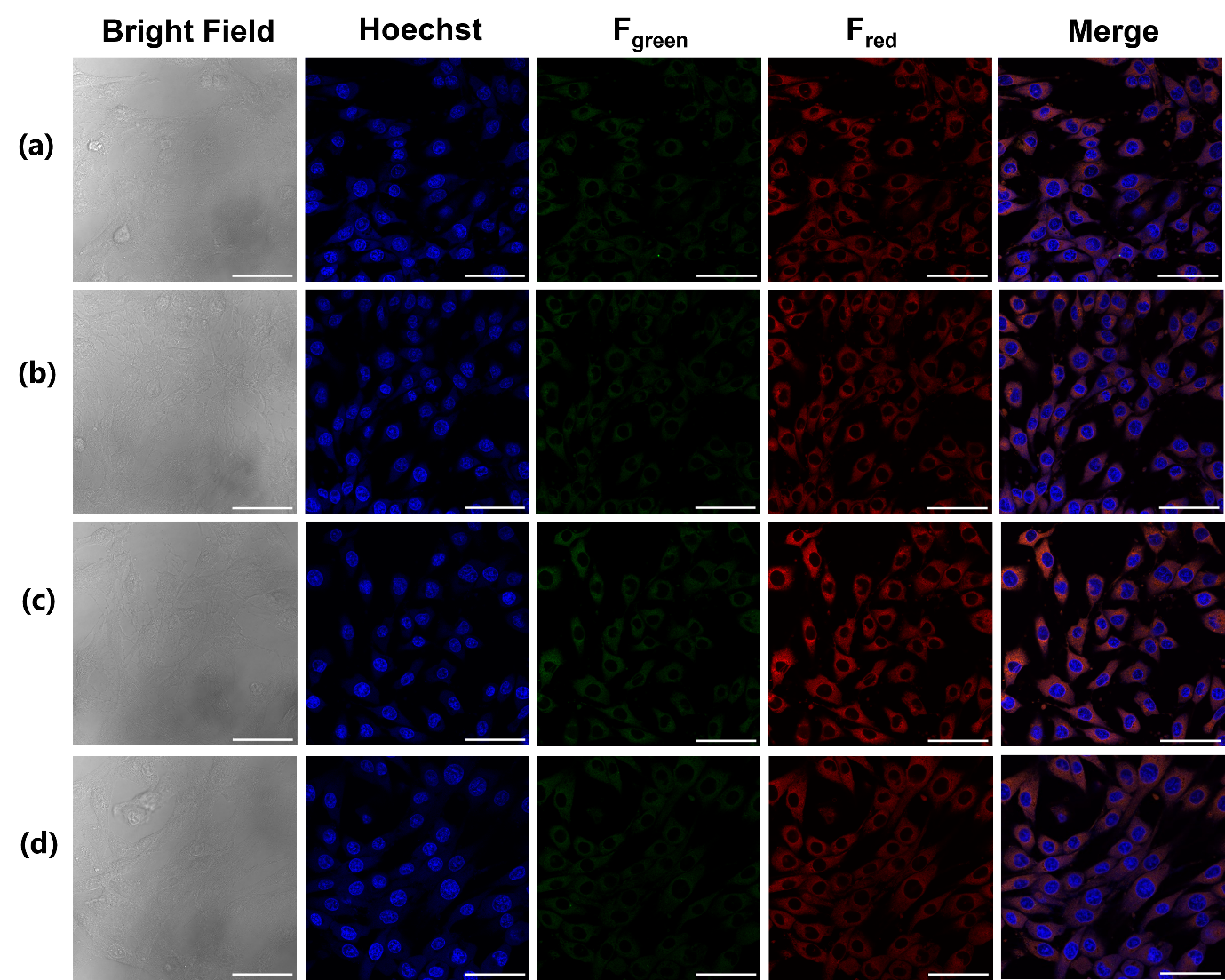
**

**Figure S25.** Functional imaging of hCYP3A4 in living U87 cells by a confocal laser scanning microscopy. The U87 (a) cells were incubated with Hoechst33342 (blue channel) and ER tracker (red channel) for 15 min. The U87 (b) cells were incubated with **NFa** (20 µM, green channel) for 1 h, Hoechst33342 (blue channel), and ER tracker (red channel) for 15 min. The U87 (c) cells were treated with RTV (20 µM) for 1 h, following staining by **NFa** for 1 h and staining by Hoechst33342 and ER tracker for 15 min. The U87 (d) cells were treated with KET (20 µM) for 1 h, following staining by **NFa** for 1 h and staining by Hoechst33342 or ER tracker for 15 min. Data are expressed as mean ± SD (n = 3). Scale bar: 50 μm.

**
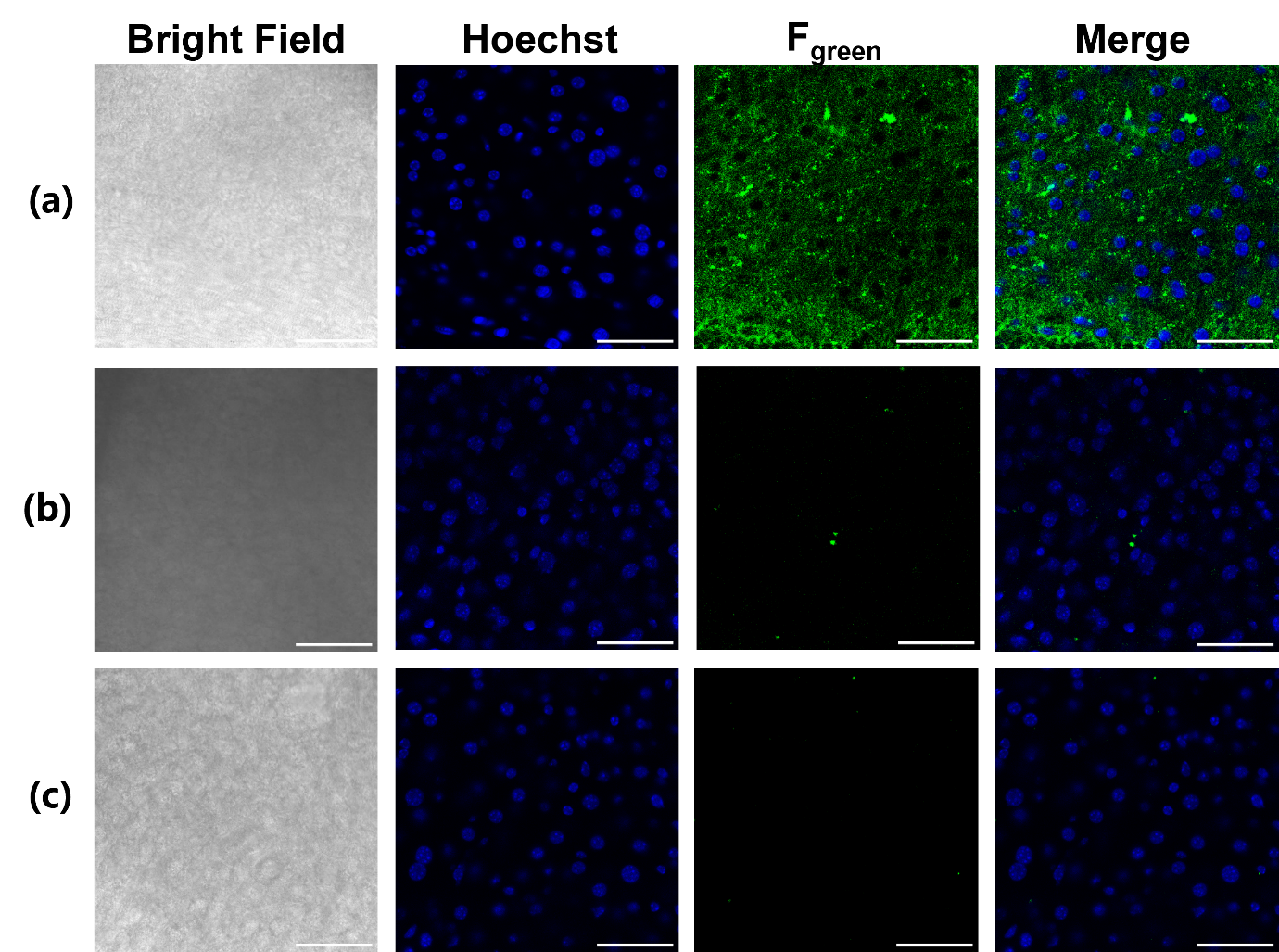
**

**Figure S26.** Functional imaging of CYP3A4 in mouse liver slices by a confocal laser scanning microscopy. The liver slices (a) were incubated with **NFa** (20 µM, green channel) for 1 h and Hoechst33342 (blue channel) for 15 min. The liver slices (b) were treated with RTV (20 µM) for 1 h, following staining by **NFa** for 1 h and staining by Hoechst33342 for 15 min. The liver slices (c) were treated with KET (20 µM) for 1 h, following staining by **NFa** for 1 h and staining by Hoechst33342 for 15 min. Data are expressed as mean ± SD (n = 3). Scale bar: 50 μm.

**
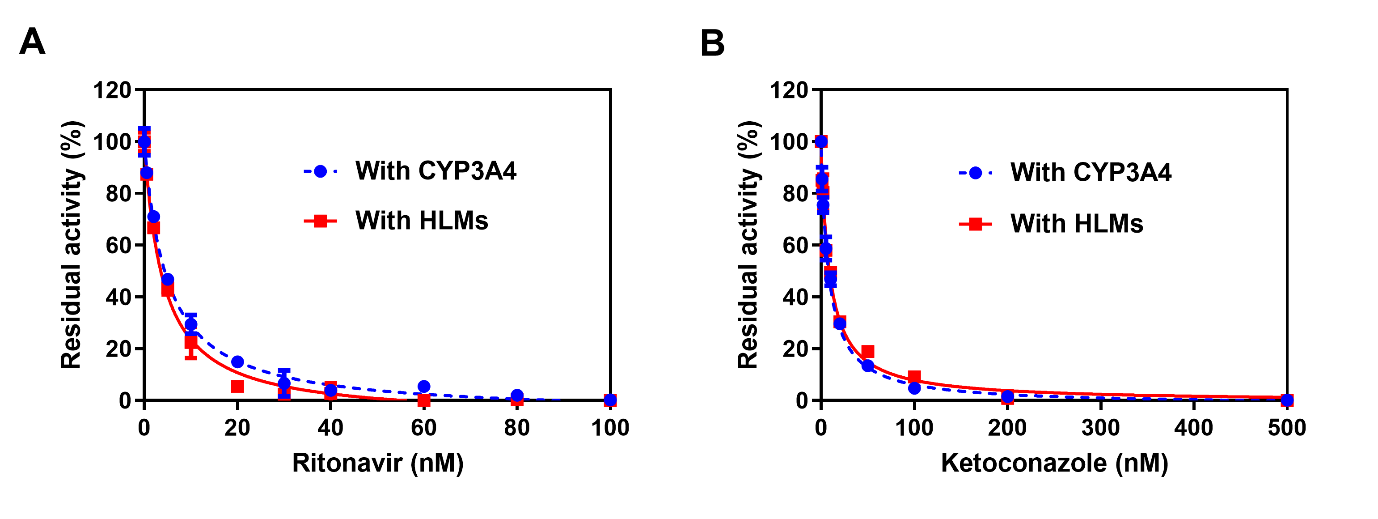
**

**Figure S27.** Dose-inhibition curves of ritonavir (A) and ketoconazole (B) on hCYP3A4 in HLMs (solid red line) and hCYP3A4 (dotted blue line). Data are expressed as mean ± SD (n = 3).

**
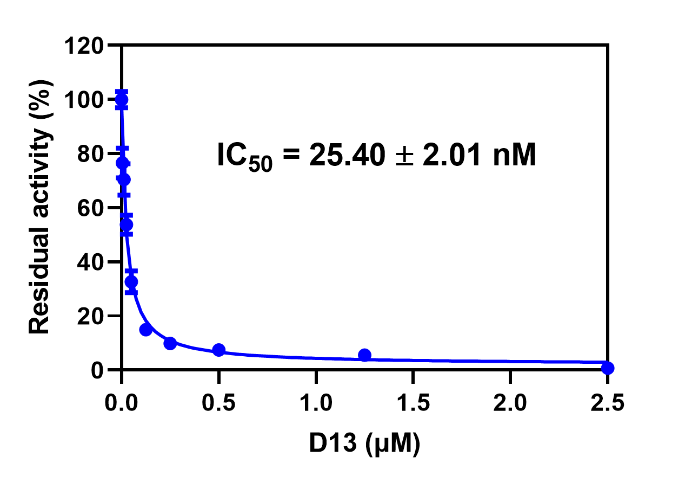
**

**Figure S28.** Dose-inhibition curves of **D13** against CYP3A4 in living CHO-3A4 cells. Data are expressed as mean ± SD (n=3).


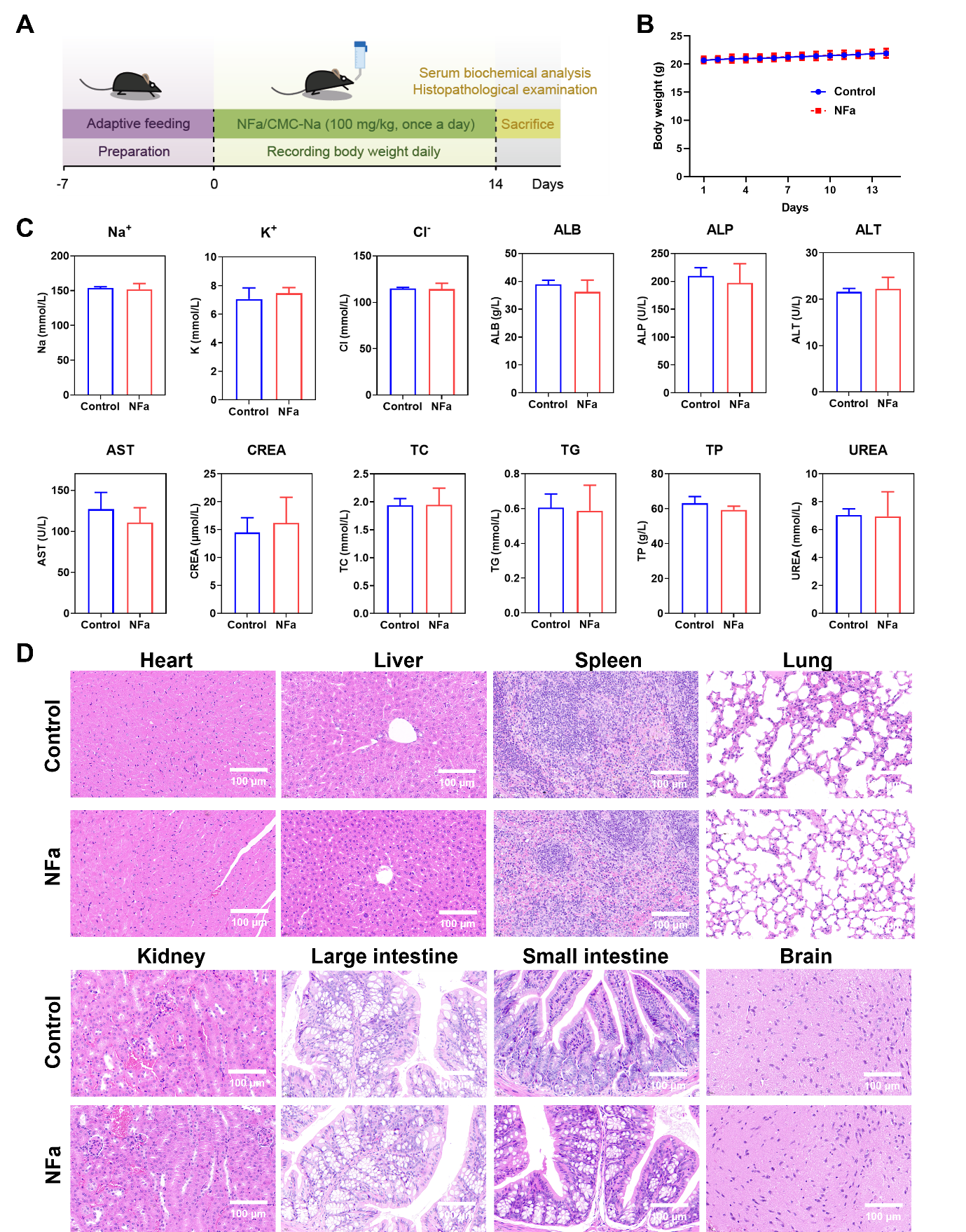


**Figure S29**. **NFa** shows good safety profiles in mice. (A) Animal experimental road map. (B) Body weight changes in mice. (C) The serum biochemical analysis in control and **NFa** groups. (D) Typical hematoxylin and eosin (H&E) staining from control and **NFa** groups. Scale bar: 100 μm. (ALB, Albumin; ALP, Alkaline phosphatase; ALT, Alanine aminotransferase; AST, Aspartate aminotransferase; CREA, Creatinine; TC, Total cholesterol; TG, Triglyceride; TP, Total protein; UREA, Urea). Data are expressed as mean ± SD (n = 5).

**
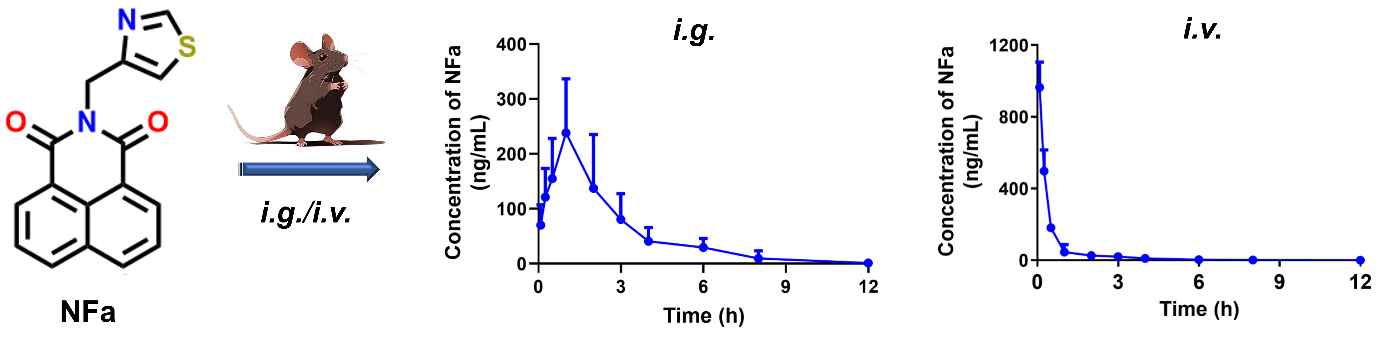
**

**Figure S30**. The mean plasma concentration-time curves of **NFa** when **NFa** was administered orally (20 mg/kg, *i.g.*, n = 6) and intravenously (2 mg/kg, *i.v.*, n = 6).


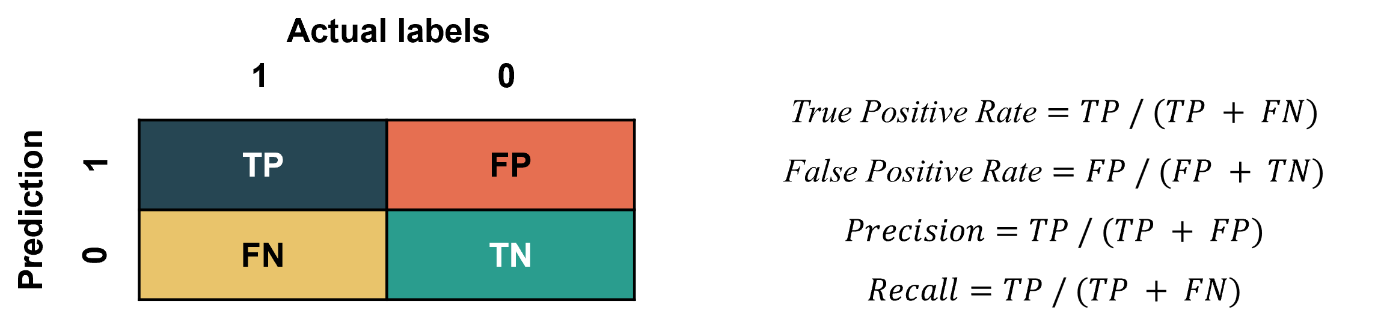


**Figure S31.** Confusion matrix diagram and the formulas of True Positive Rate, False Positive Rate, Precision, and Recall. In addition, AUC-ROC represents the area under the receiver operating characteristic (ROC) curve, which plots the true positive rate (TPR) against the false positive rate (FPR). AUPR represents the area under the precision-recall curve, which plots precision against recall. These metrics were calculated using the ‘sklearn.metrics’ toolkit.


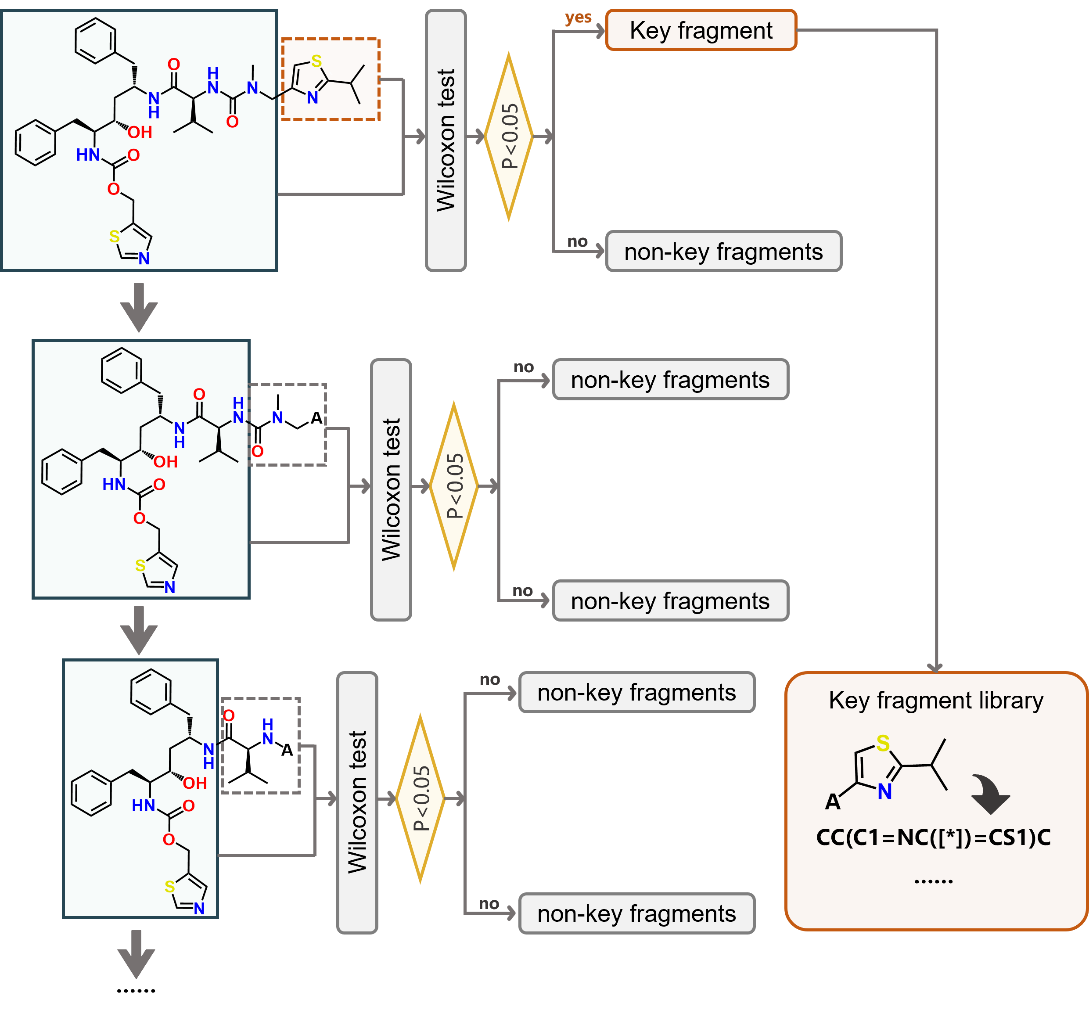


**Figure S32.** Scheme of Wilcoxon test method.

**References**

1. Frisch, M. J.; et al, Gaussian 16 Rev. B.01.Journal, **2016**.
2. T. Lu, F. Chen, *J. Comput. Chem*. **2012**, *33*, 580.
3. W. Humphrey, A. Dalke, K. *J. Mol. Graph* **1996**, *14*, 33.
4. B. C. Jones, A. Srivastava, N. Colclough, J. Wilson, V. P. Reddy, S. Amberntsson, D. Li, *Drug Metab. Dispos*. **2017**, *45*, 1060.
5. R. S. Obach, *Drug Metab. Dispos*. **2004**, *32*, 89.
6. R. T. Mayer, K. J. Netter, F. Heubel, A. Buchheister, M. D. Burke, *Biochem. Pharmacol*. **1989**, *38*, 1364.
7. V. P. Miller, D. M. Stresser, A. P. Blanchard, S. Turner, C. L. Crespi, *Ann. N. Y. Acad. Sci*. **2000**, *919*, 26.
8. D. M. Stresser, A. P. Blanchard, S. D. Turner, J. C. Erve, A. A. Dandeneau, V. P. Miller, C. L. Crespi, *Drug Metab. Dispos*. **2000**, *28*, 1440.
9. J. Ning, W. Wang, G. Ge, P. Chu, F. Long, Y. Yang, Y. Peng, L. Feng, X. Ma, T. D. James, *Angew. Chem., Int. Ed.* **2019**, *58*, 9959.
10. R. J. He, Z. H. Tian, J. Huang, M. R. Sun, F. Wei, C. Y. Li, H. R. Zeng, F. Zhang, X. Q. Guan, Y. Feng, X. M. Meng, H. Yang, G. B. Ge, *J. Med. Chem*. **2023**, *66*, 6743.

**3.** **^1^H and ^13^C NMR Spectra of All New Compounds**


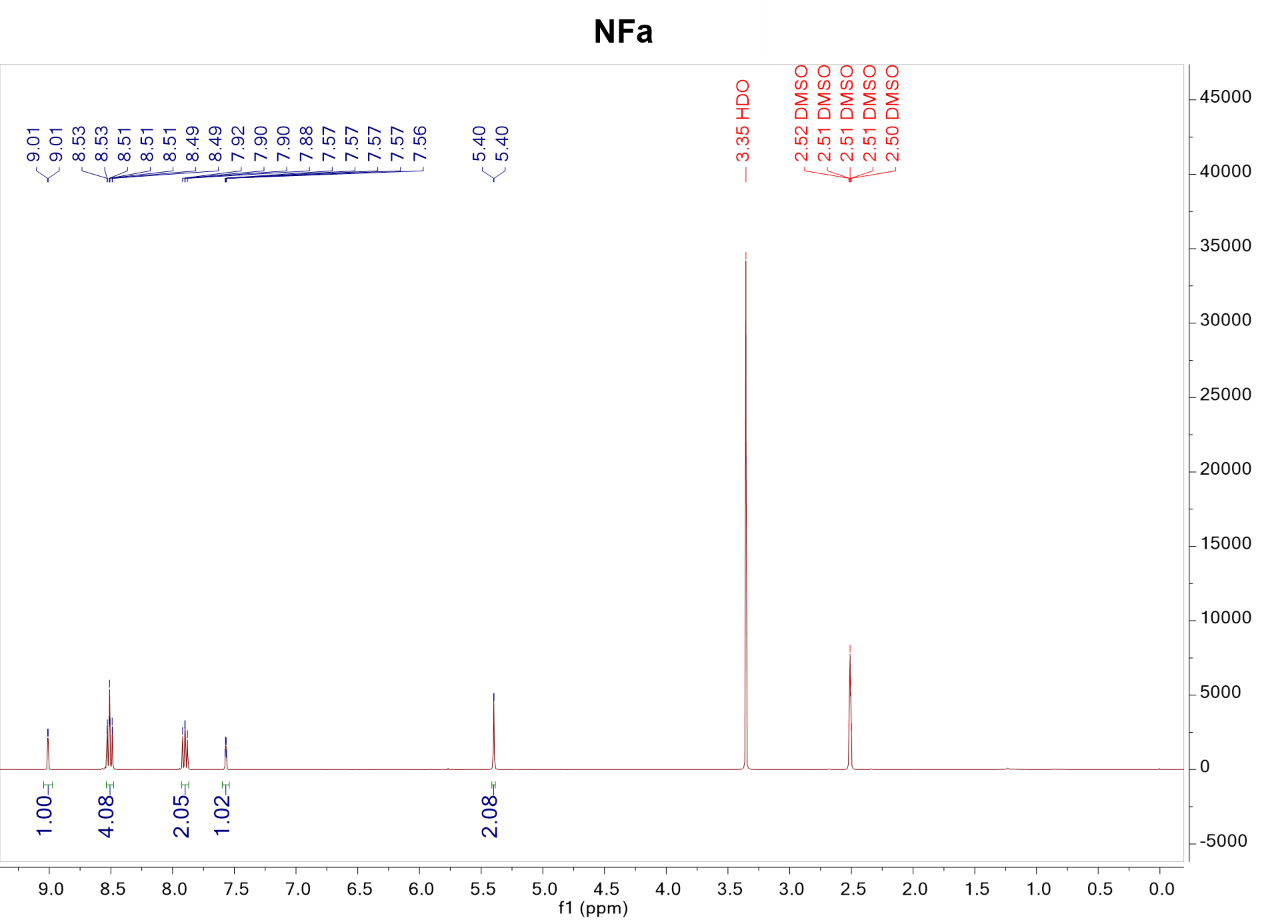


**Figure S33.** ^1^H NMR spectra of compound **NFa** in DMSO-d6.


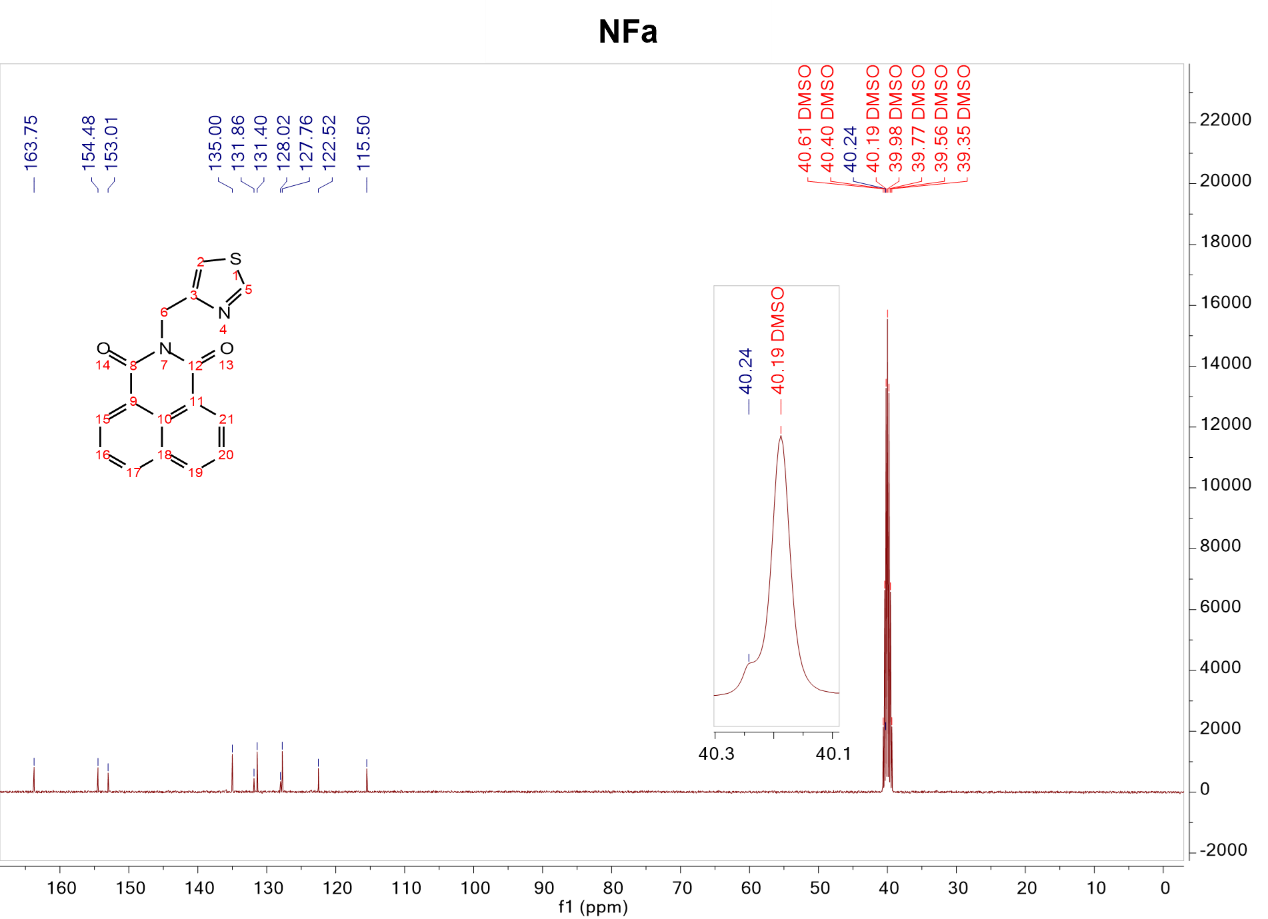


**Figure S34.** ^13^C NMR spectra of compound **NFa** in DMSO-d6.


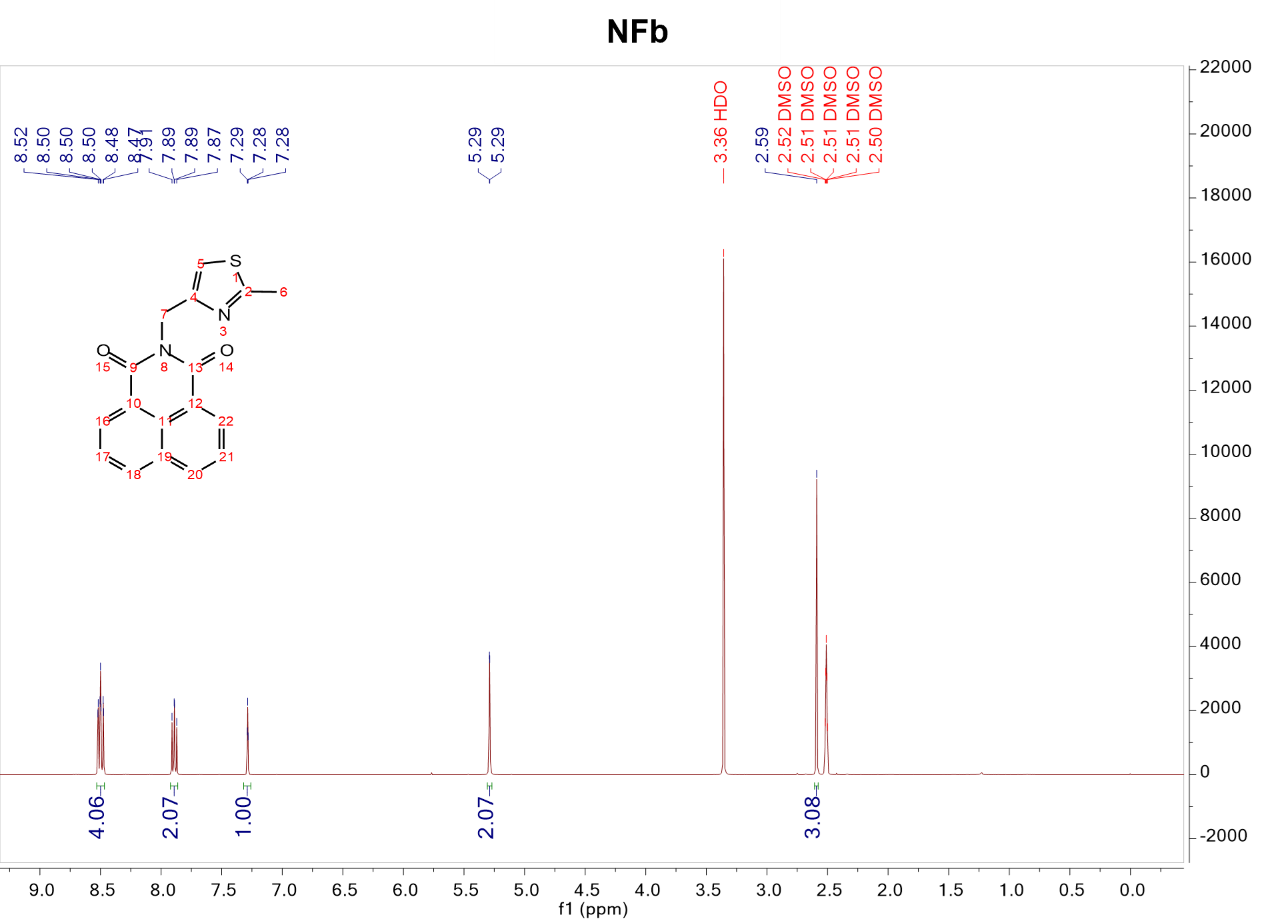


**Figure S35.** ^1^H NMR spectra of compound **NFb** in DMSO-d6.


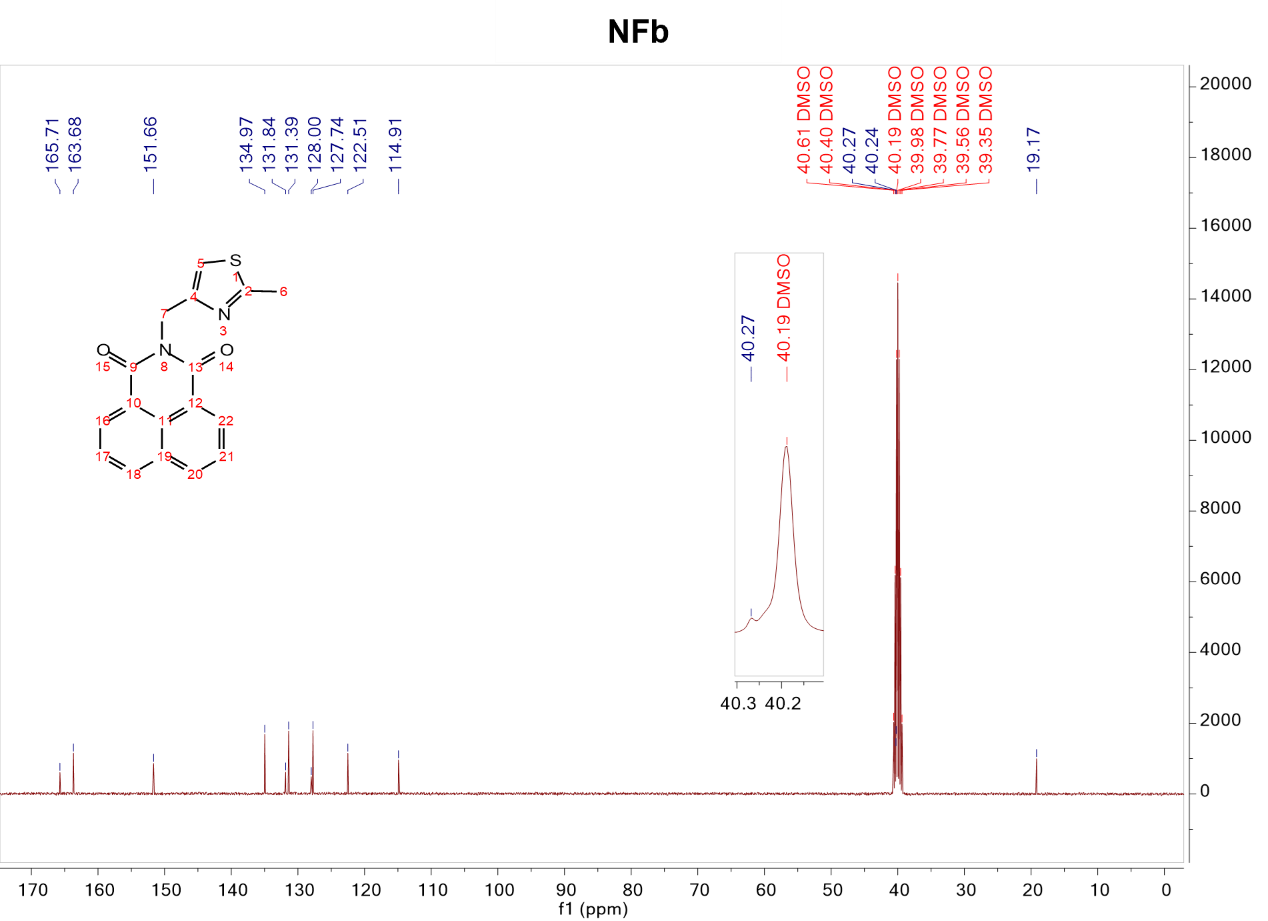


**Figure S36.** ^13^C NMR spectra of compound **NFb** in DMSO-d6.


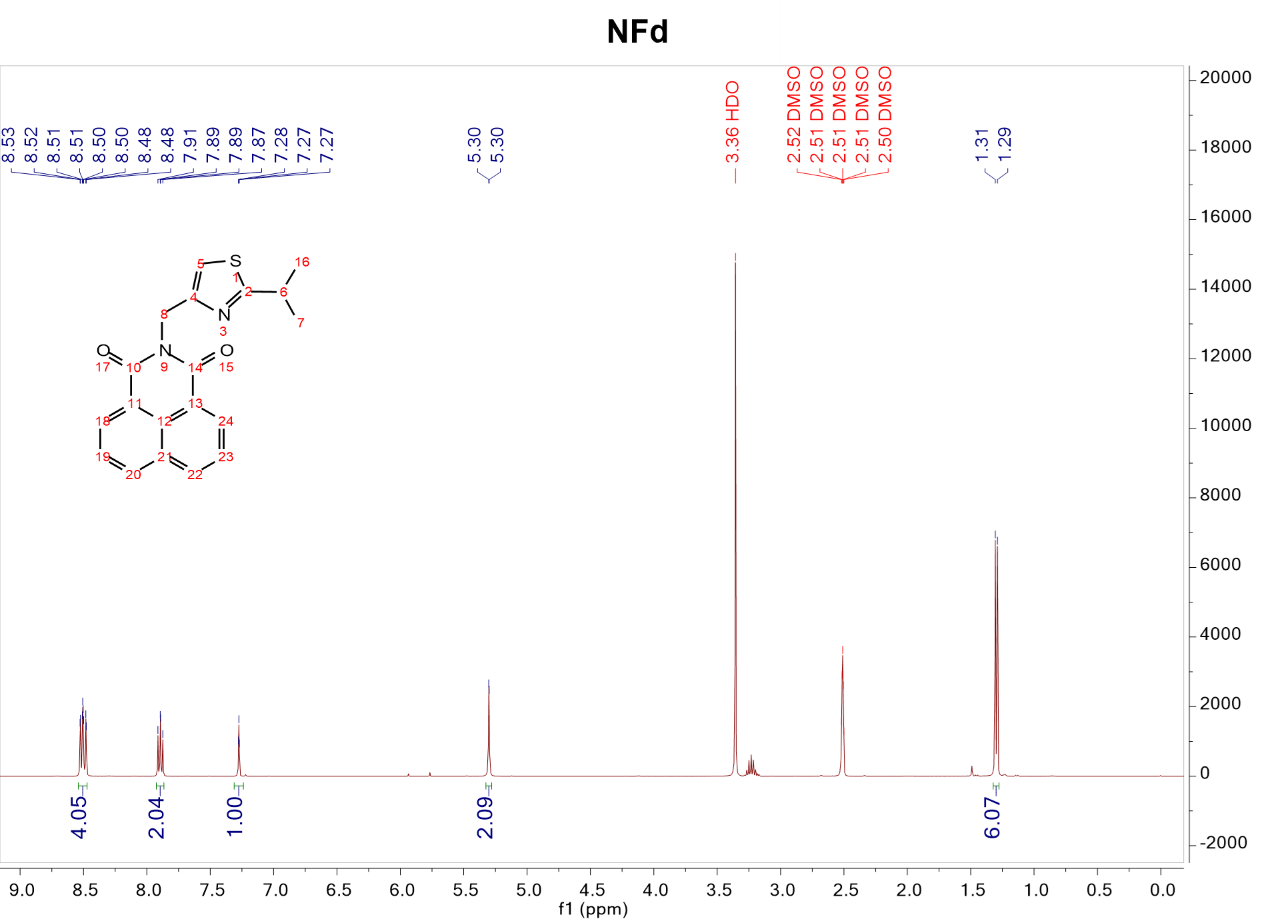


**Figure S37.** ^1^H NMR spectra of compound **NFd** in DMSO-d6.


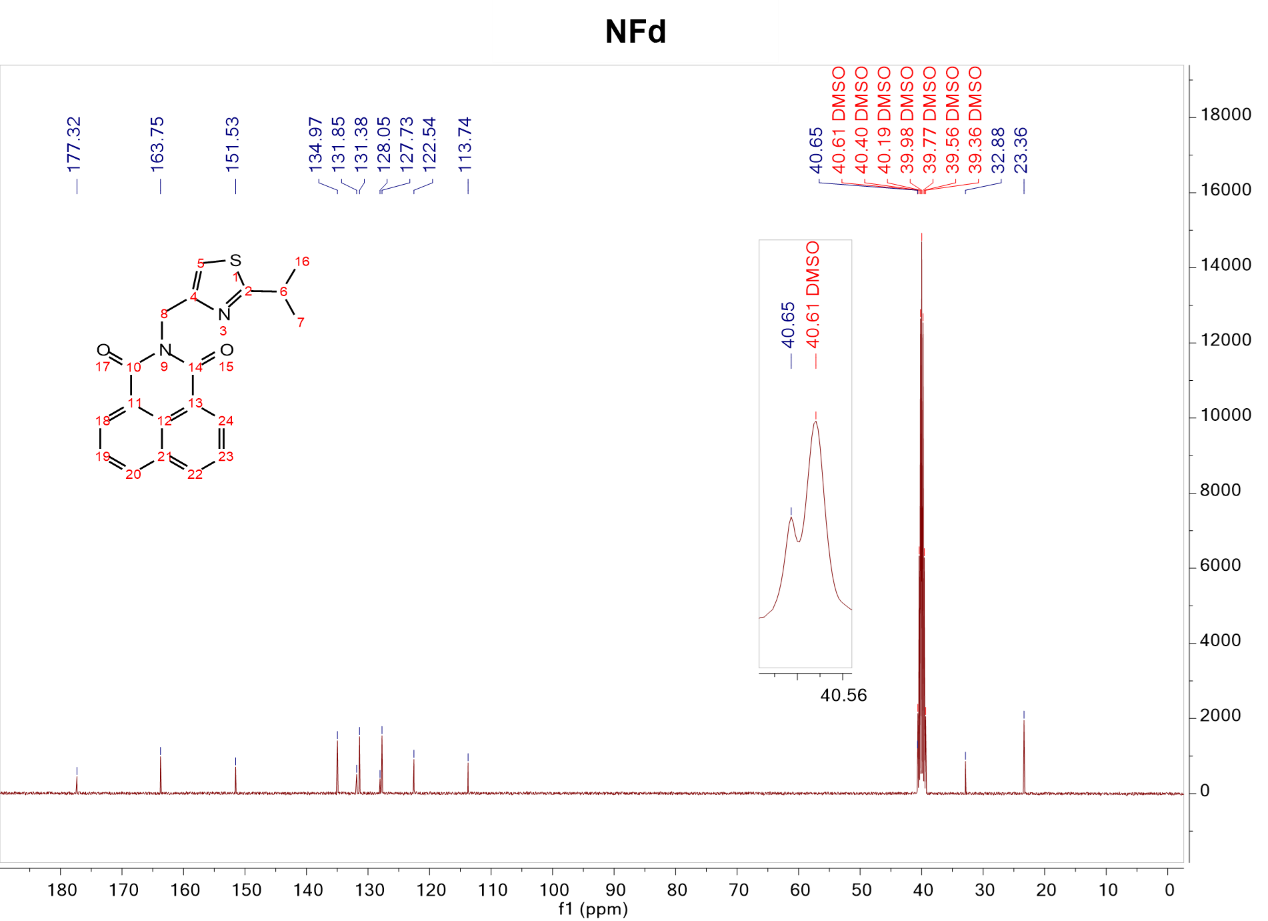


**Figure S38.** ^13^C NMR spectra of compound **NFd** in DMSO-d6.


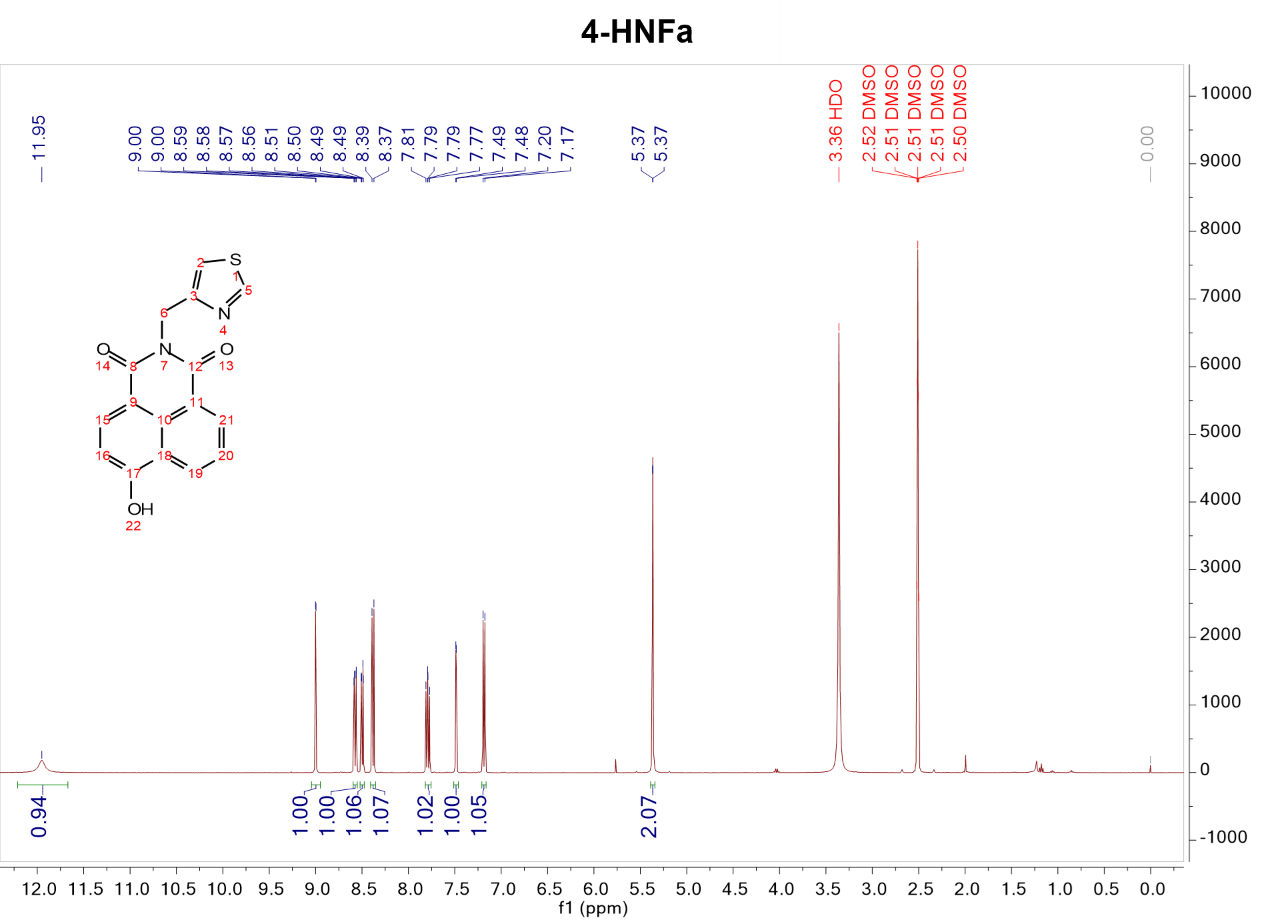


**Figure S39.** ^1^H NMR spectra of compound **4-HNFa** in DMSO-d6.


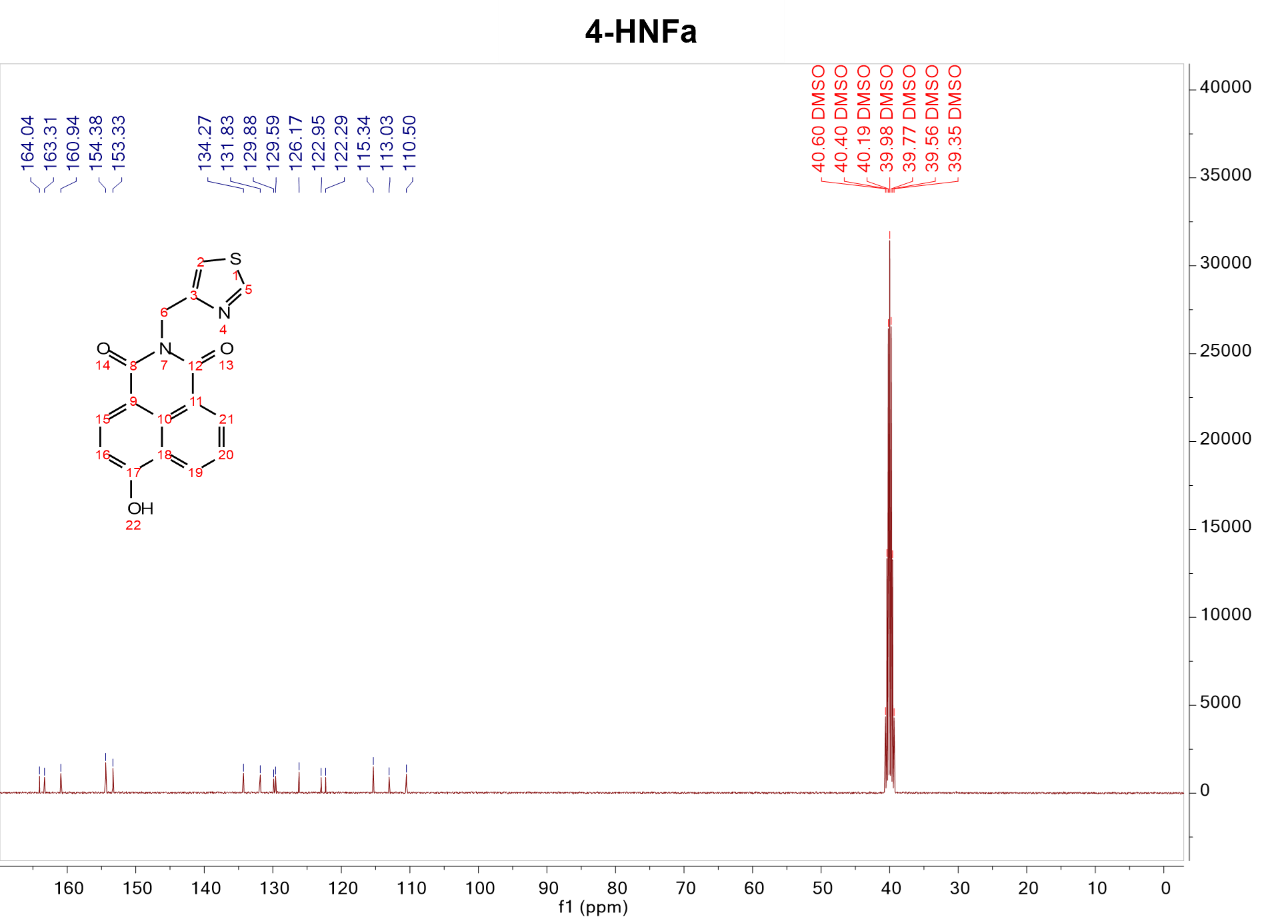


**Figure S40.** ^13^C NMR spectra of compound **4-HNFa** in DMSO-d6.


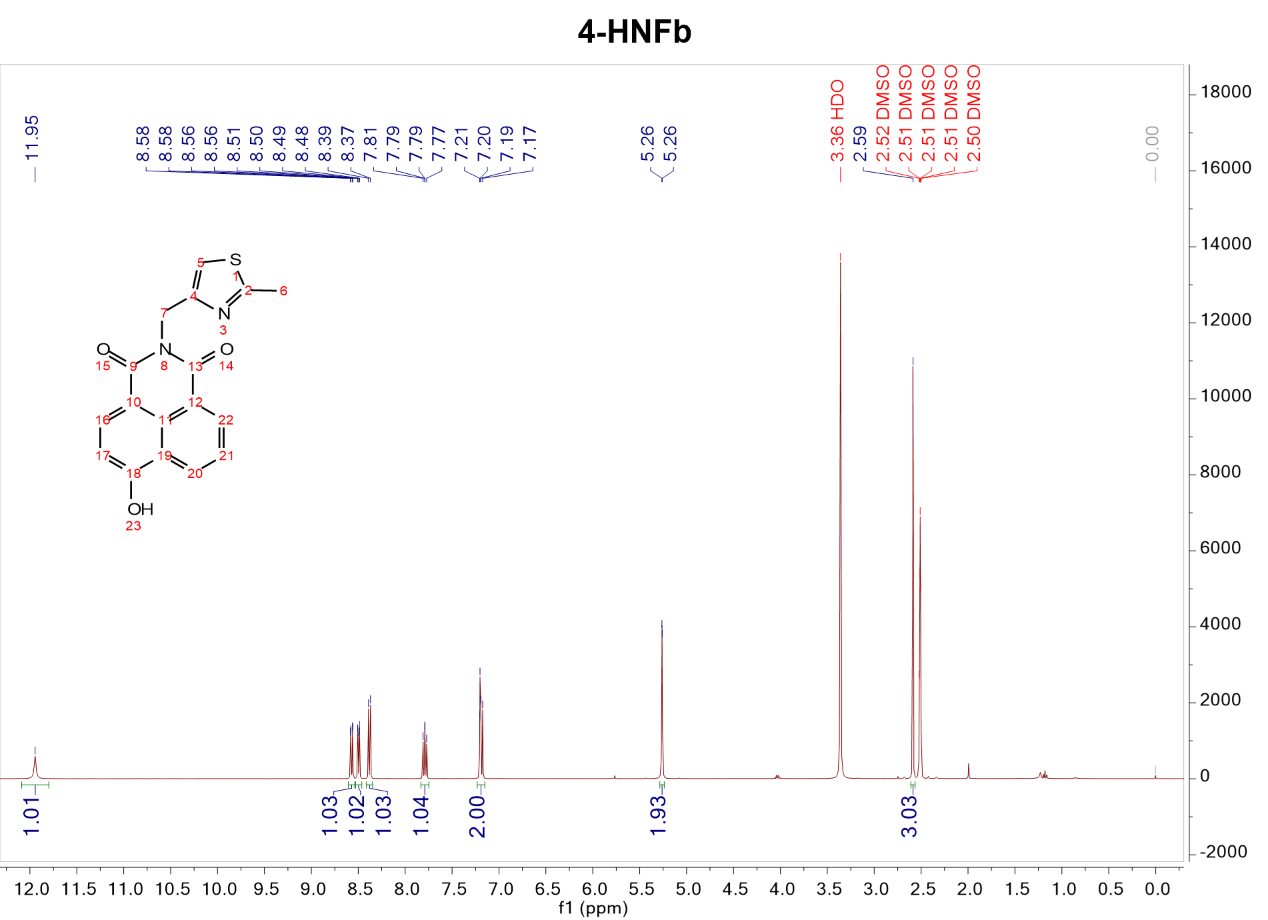


**Figure S41.** ^1^H NMR spectra of compound **4-HNFb** in DMSO-d6.


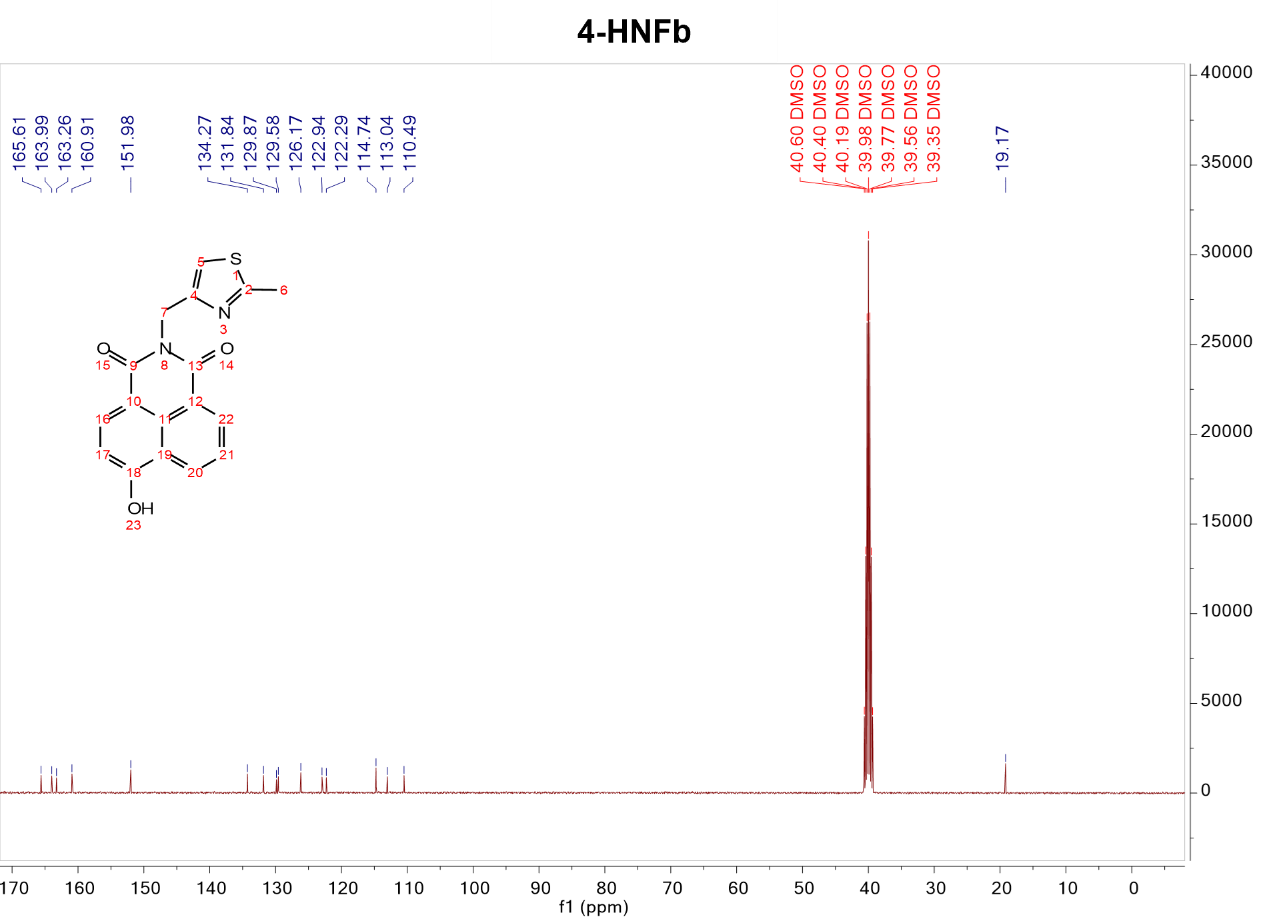


**Figure S42.** ^13^C NMR spectra of compound **4-HNFb** in DMSO-d6.


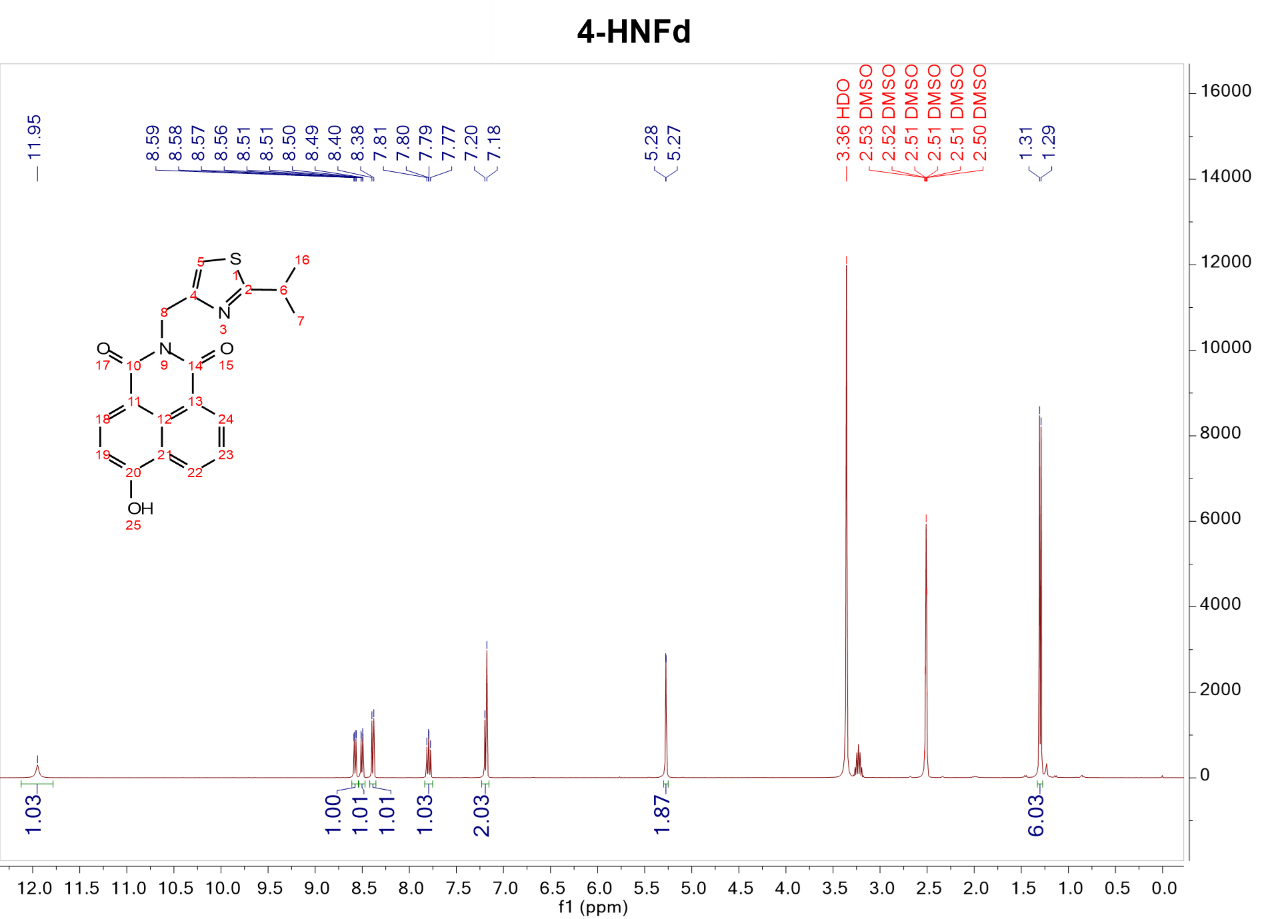


**Figure S43.** ^1^H NMR spectra of compound **4-HNFd** in DMSO-d6.


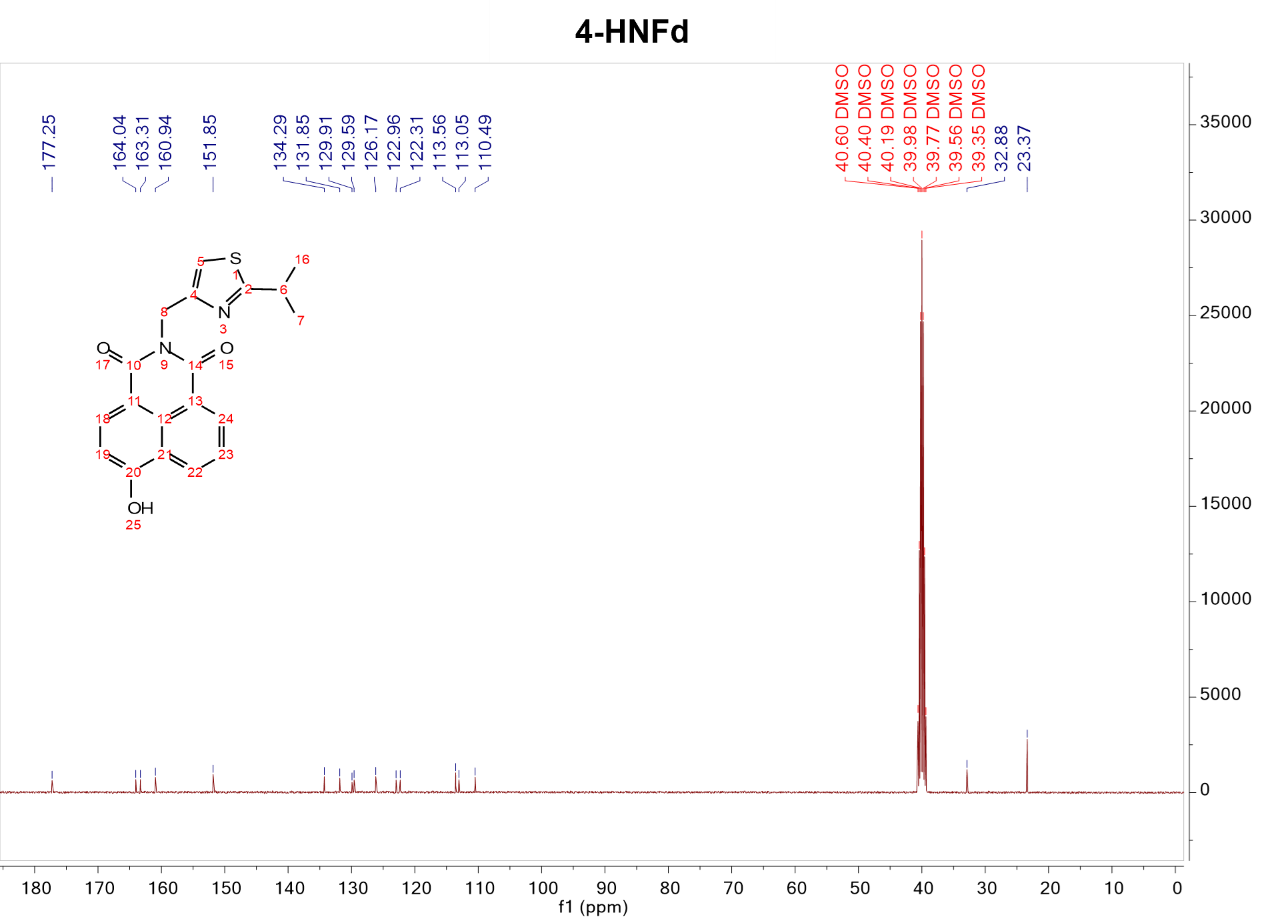


**Figure S44.** ^13^C NMR spectra of compound **4-HNFd** in DMSO-d6.


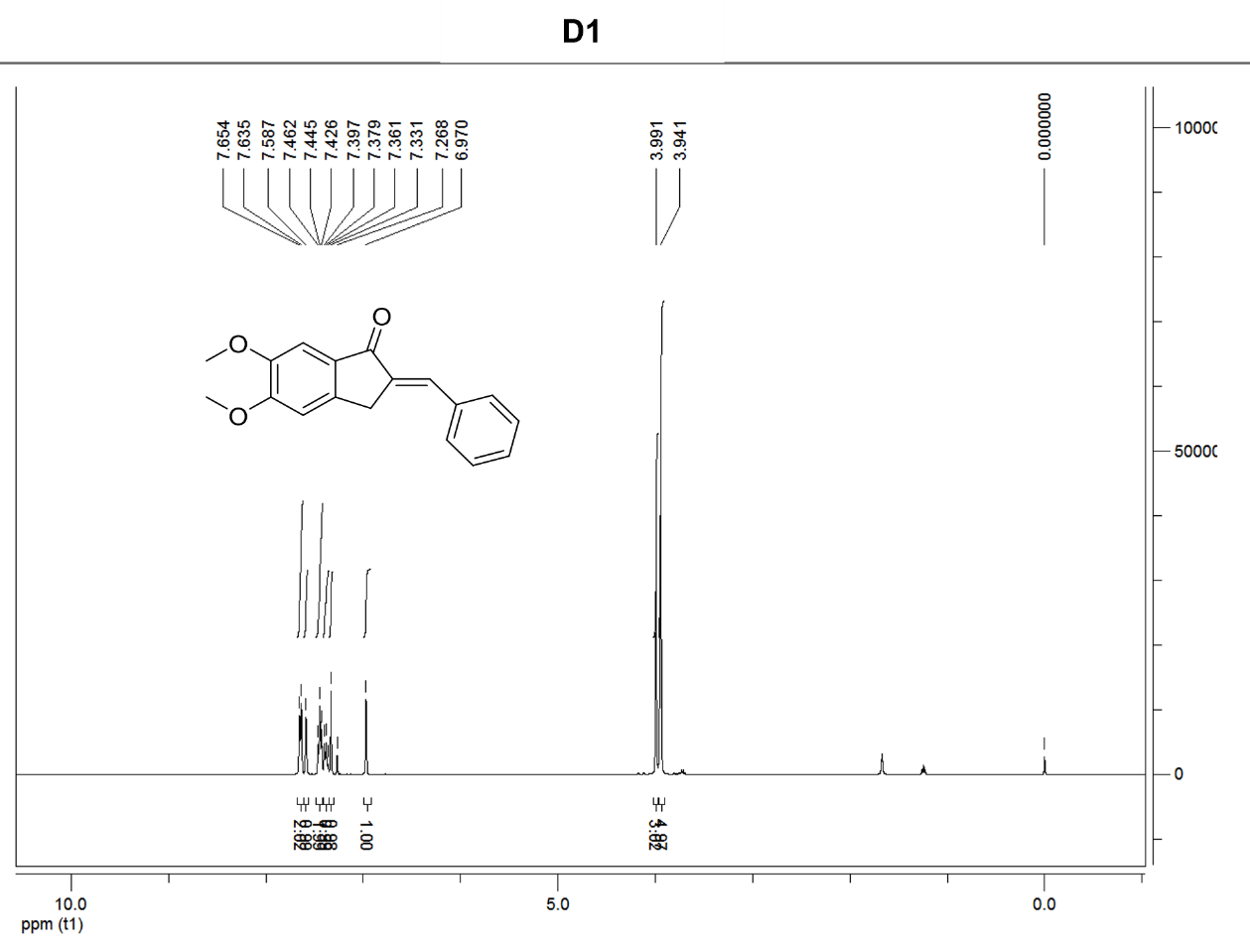


**Figure S45.** ^1^H NMR spectra of compound **D1**.


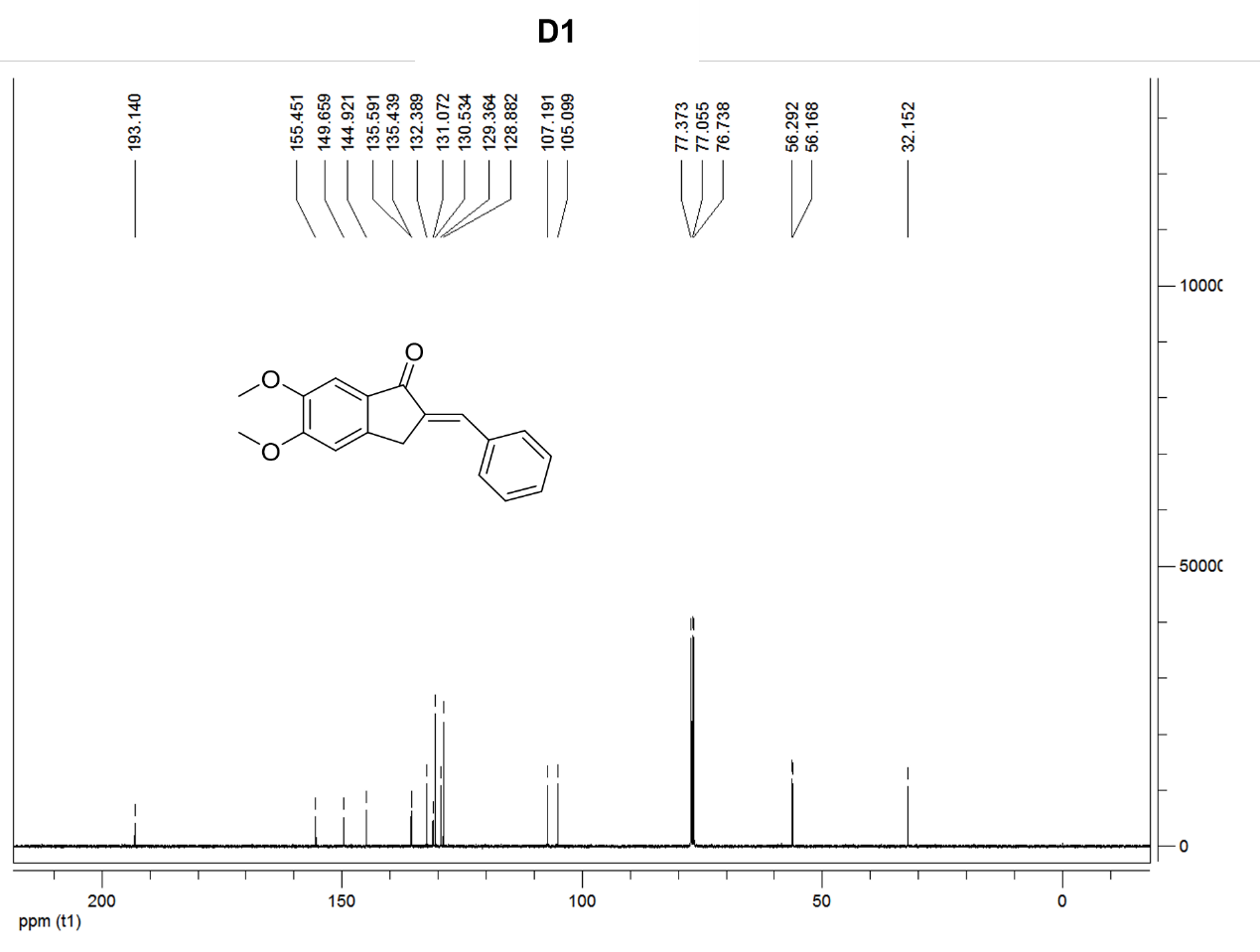


**Figure S46.** ^13^C NMR spectra of compound **D1**.


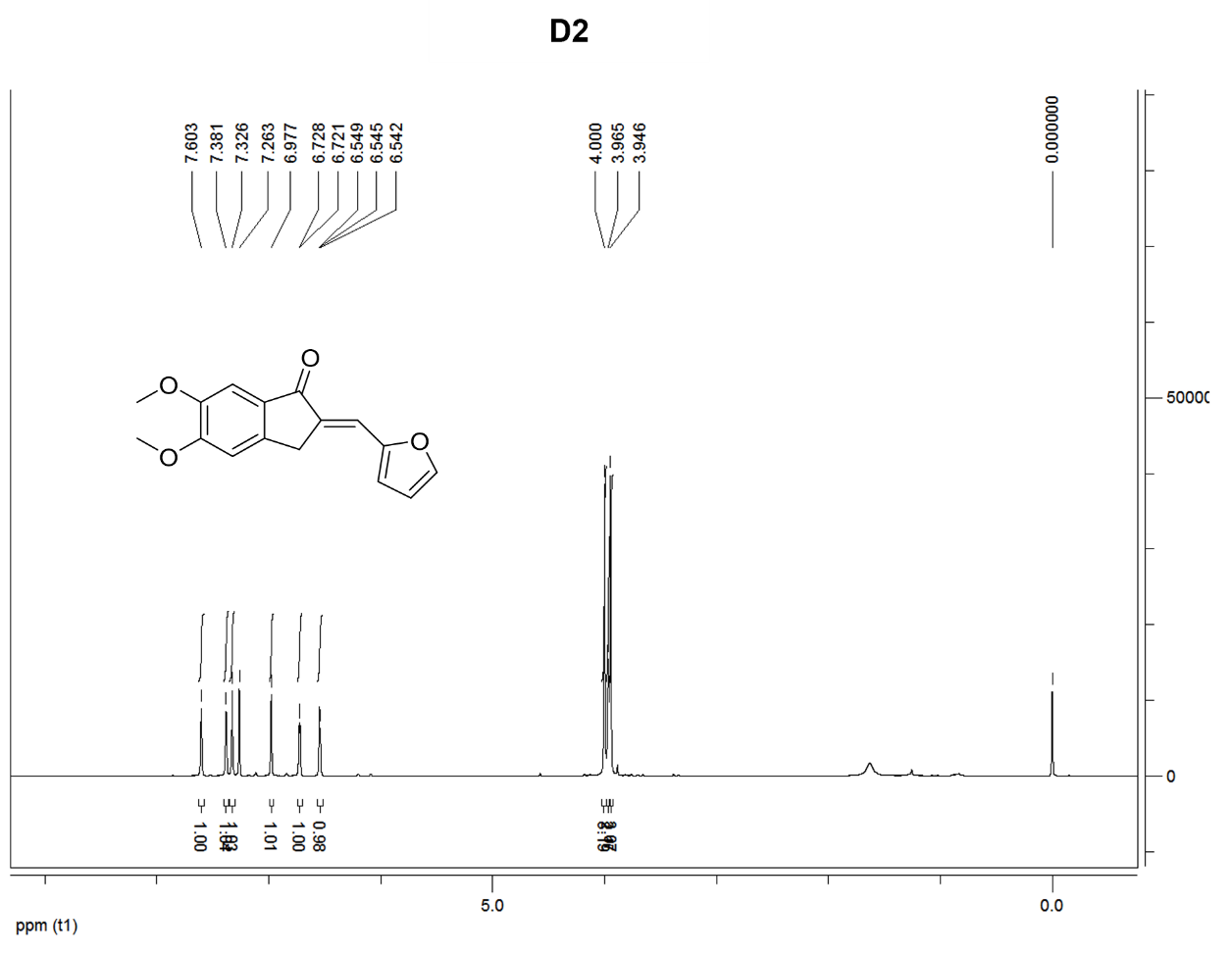


**Figure S47.** ^1^H NMR spectra of compound **D2**.


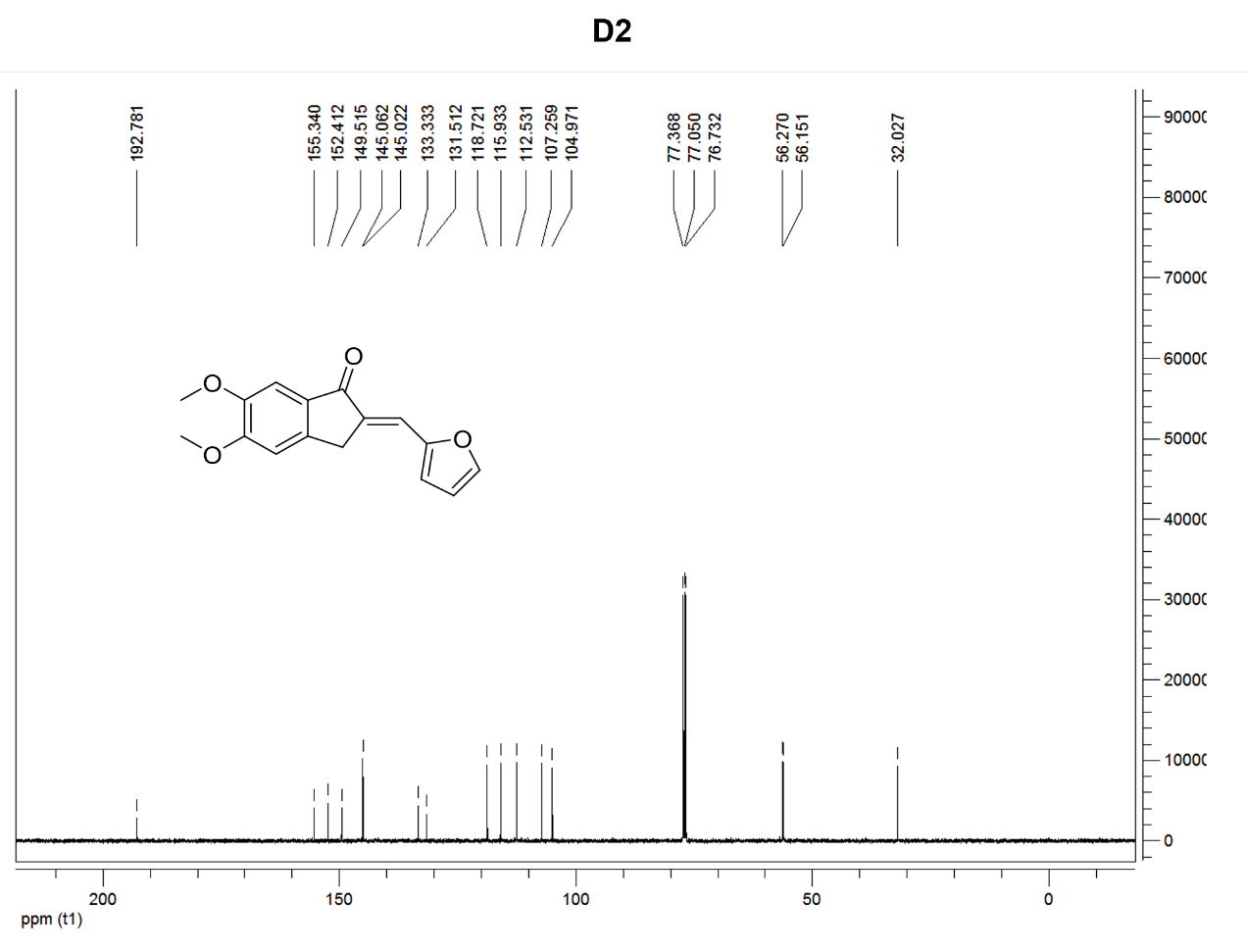


**Figure S48.** ^13^C NMR spectra of compound **D2**.


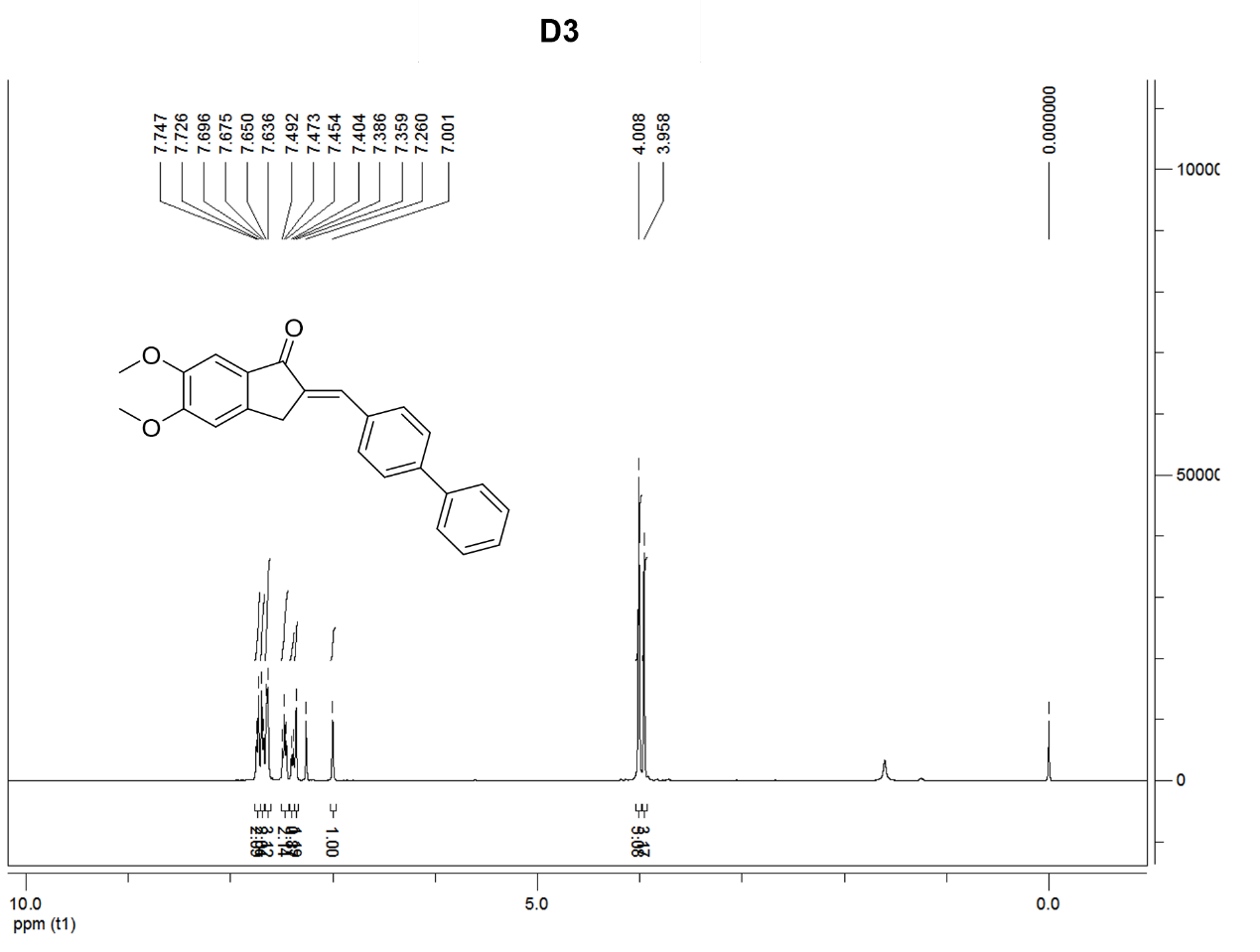


**Figure S49.** ^1^H NMR spectra of compound **D3**.


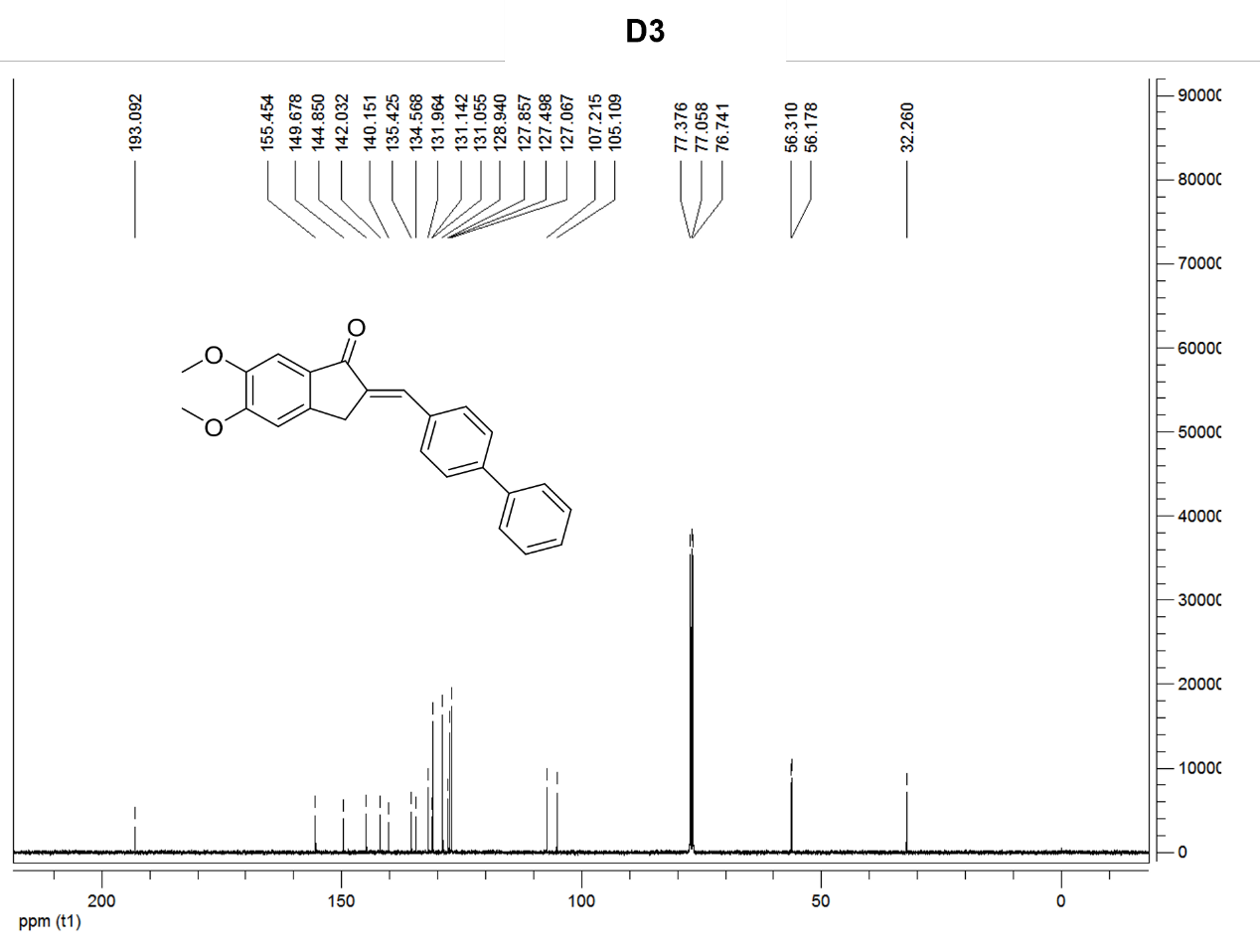


**Figure S50.** ^13^C NMR spectra of compound **D3**.


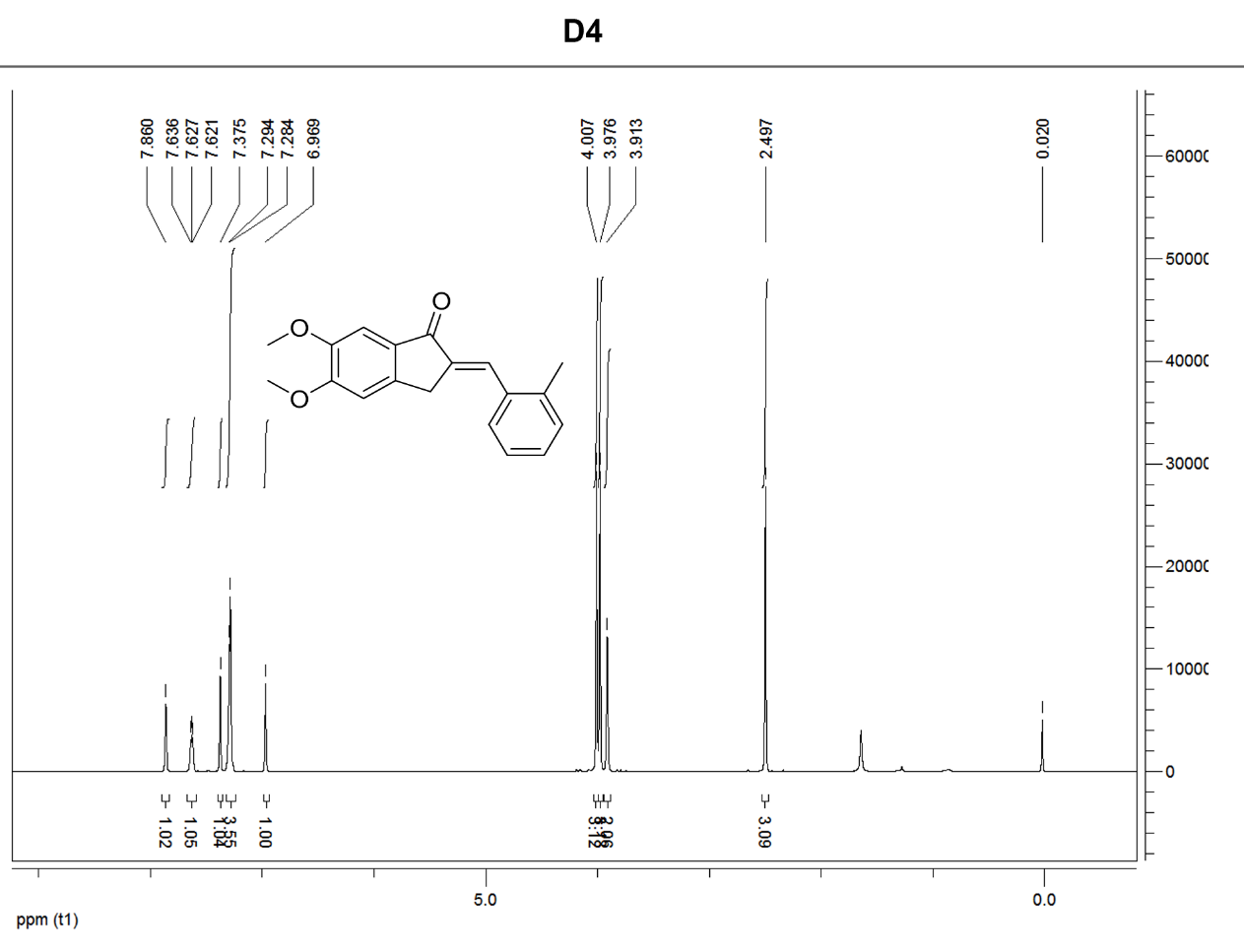


**Figure S51.** ^1^H NMR spectra of compound **D4**.


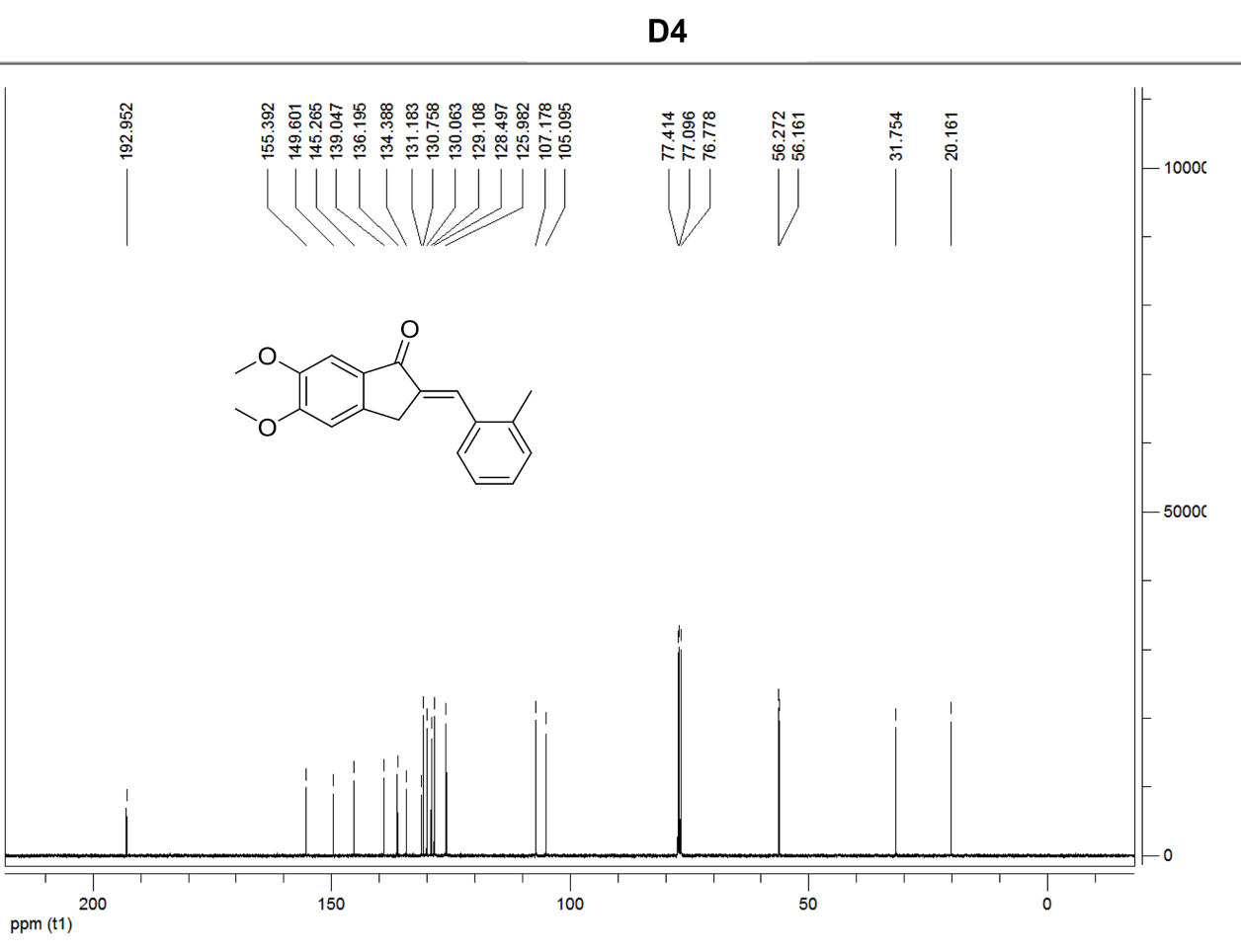


**Figure S52.** ^13^C NMR spectra of compound **D4**.


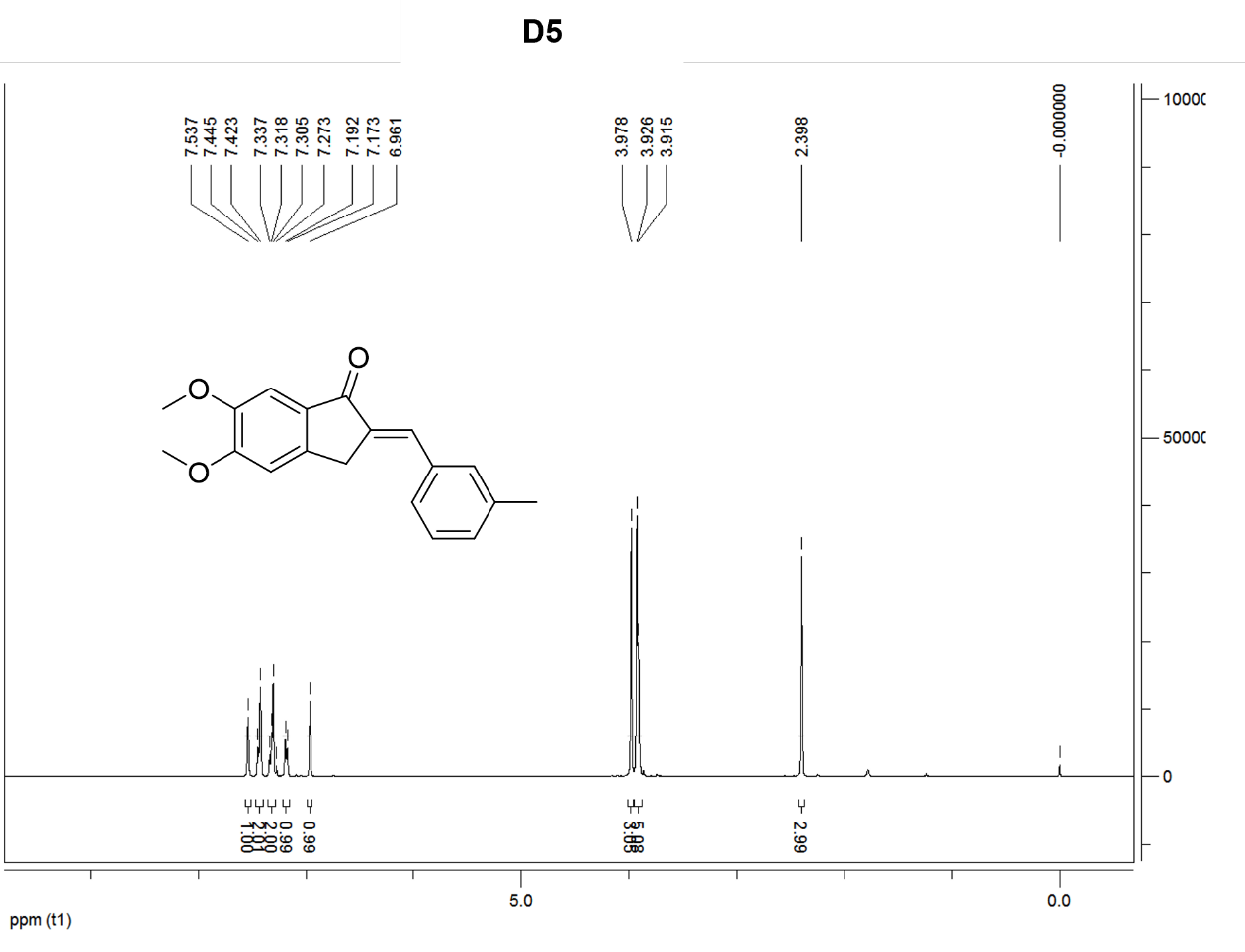


**Figure S53.** ^1^H NMR spectra of compound **D5**.


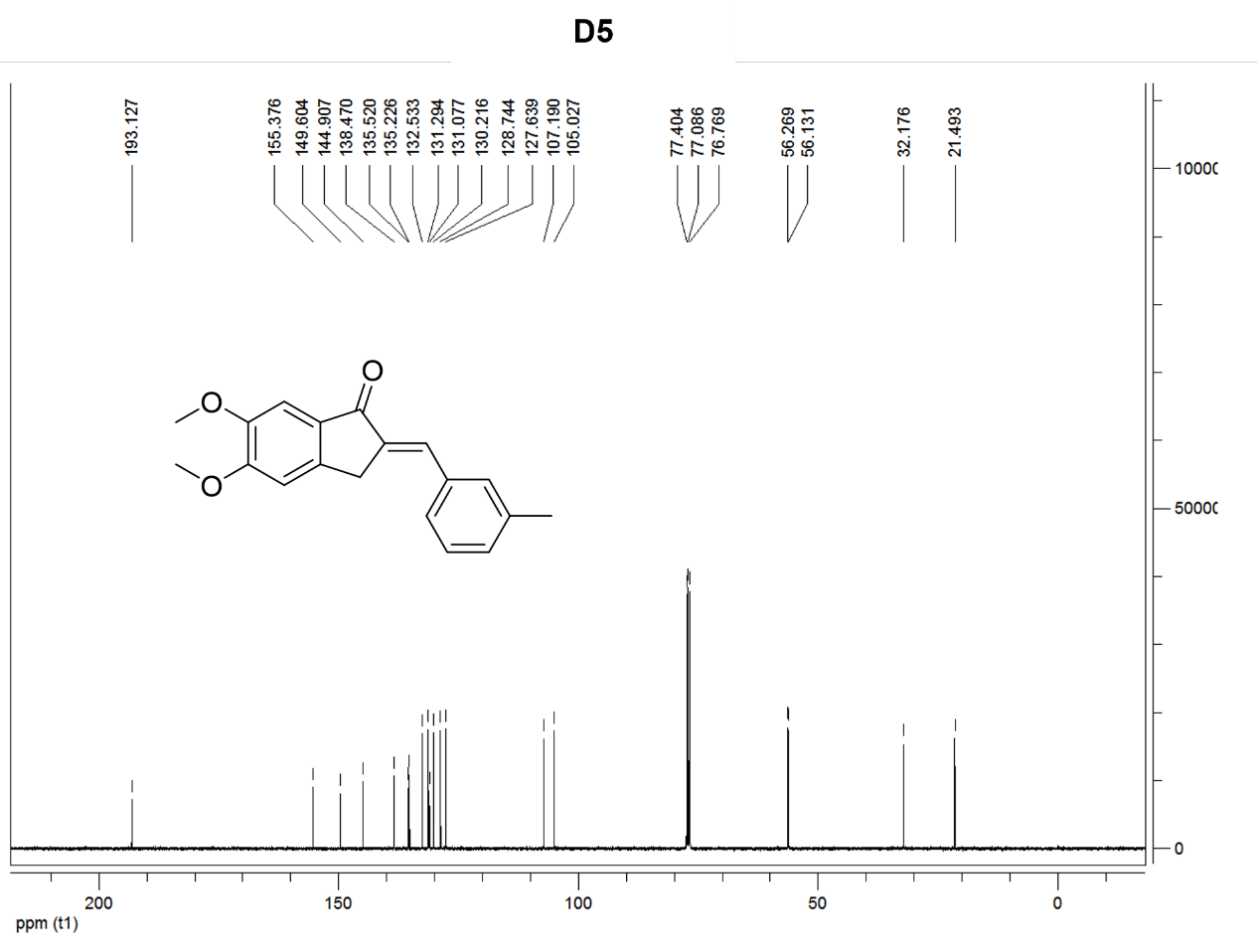


**Figure S54.** ^13^C NMR spectra of compound **D5**.


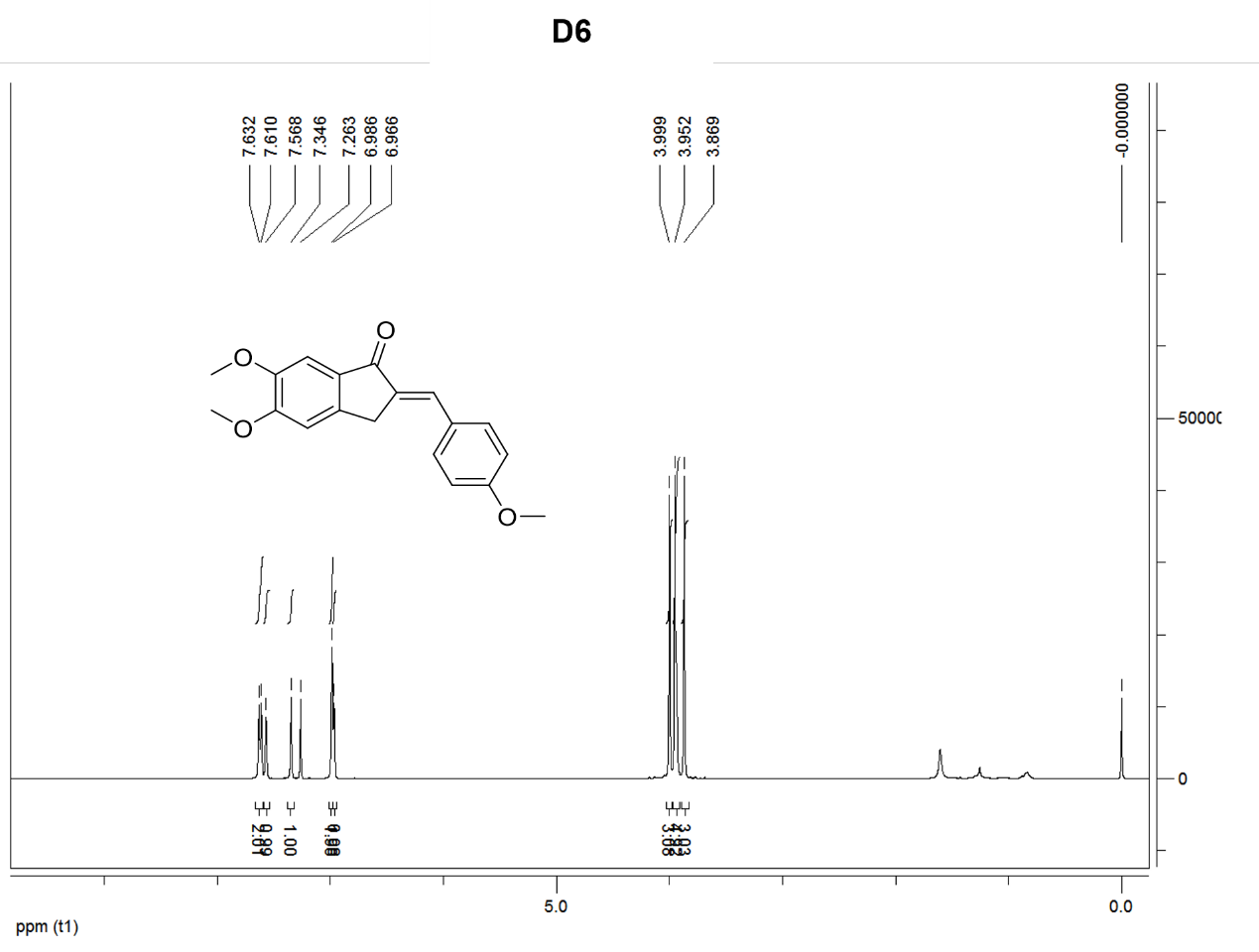


**Figure S55.** ^1^H NMR spectra of compound **D6**.


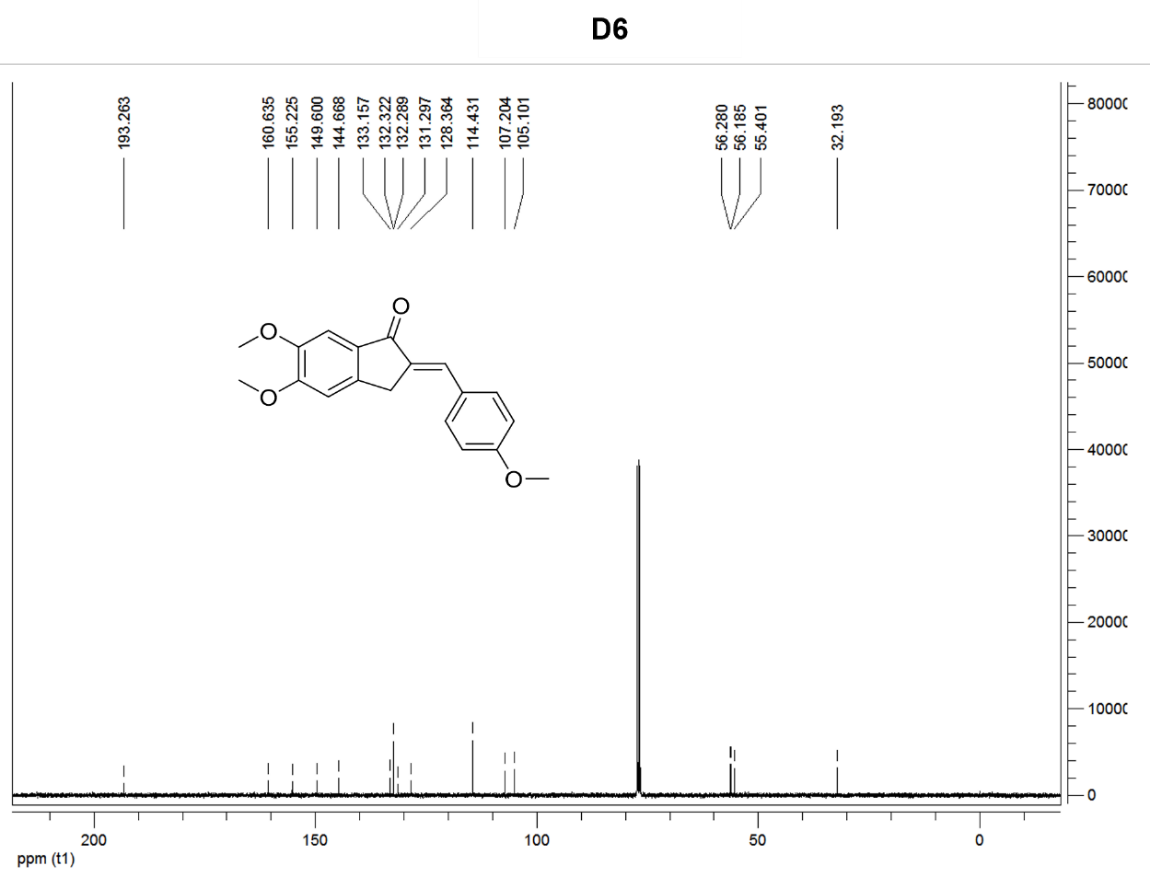


**Figure S56.** ^13^C NMR spectra of compound **D6**.


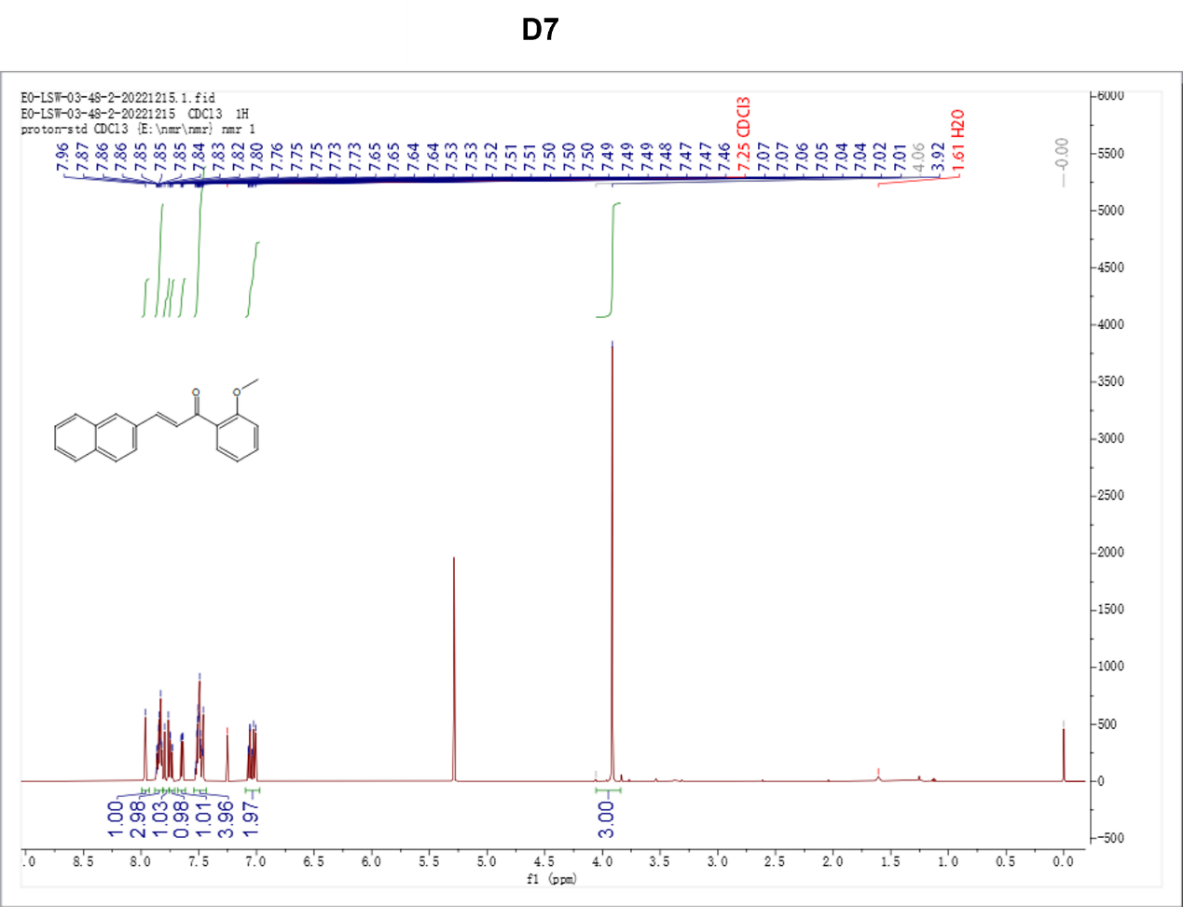


**Figure S57.** ^1^H NMR spectra of compound **D7**.


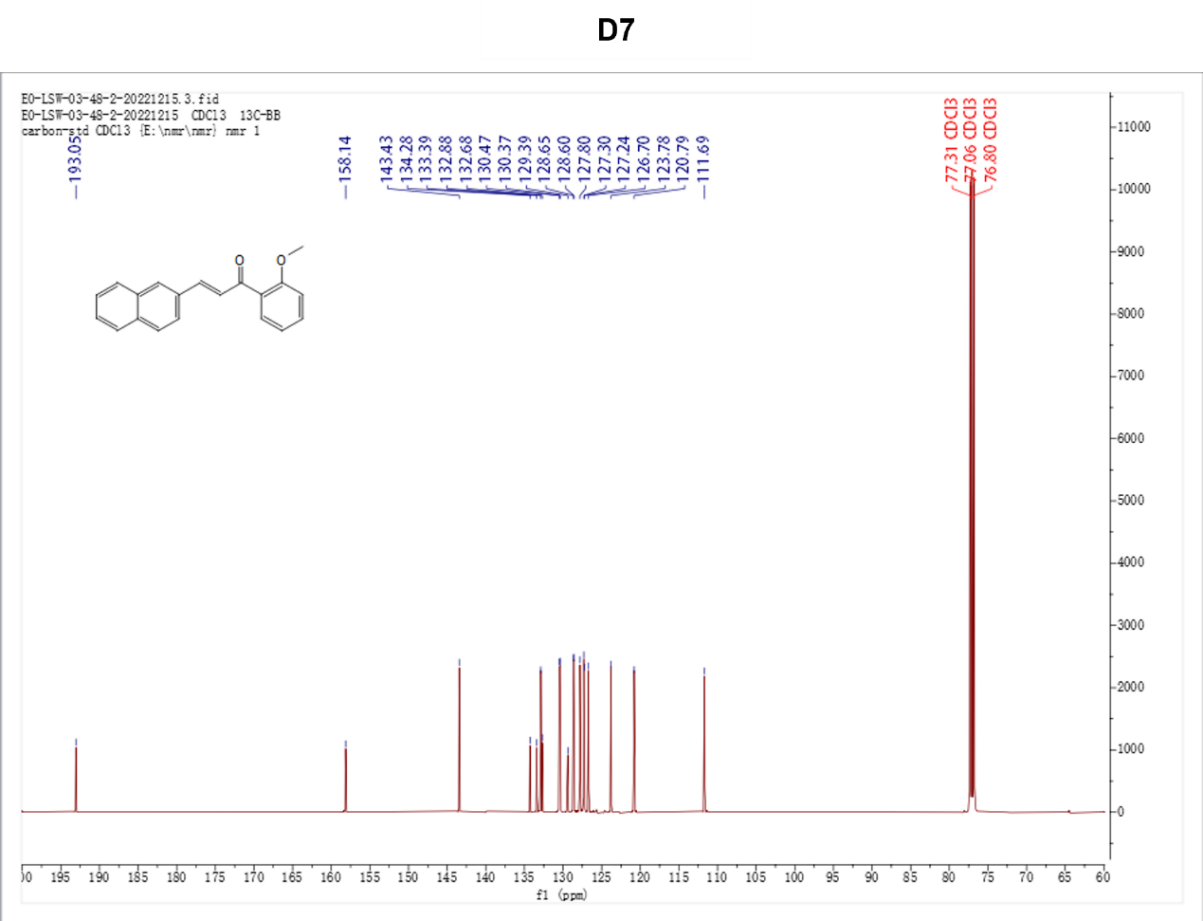


**Figure S58.** ^13^C NMR spectra of compound **D7**.


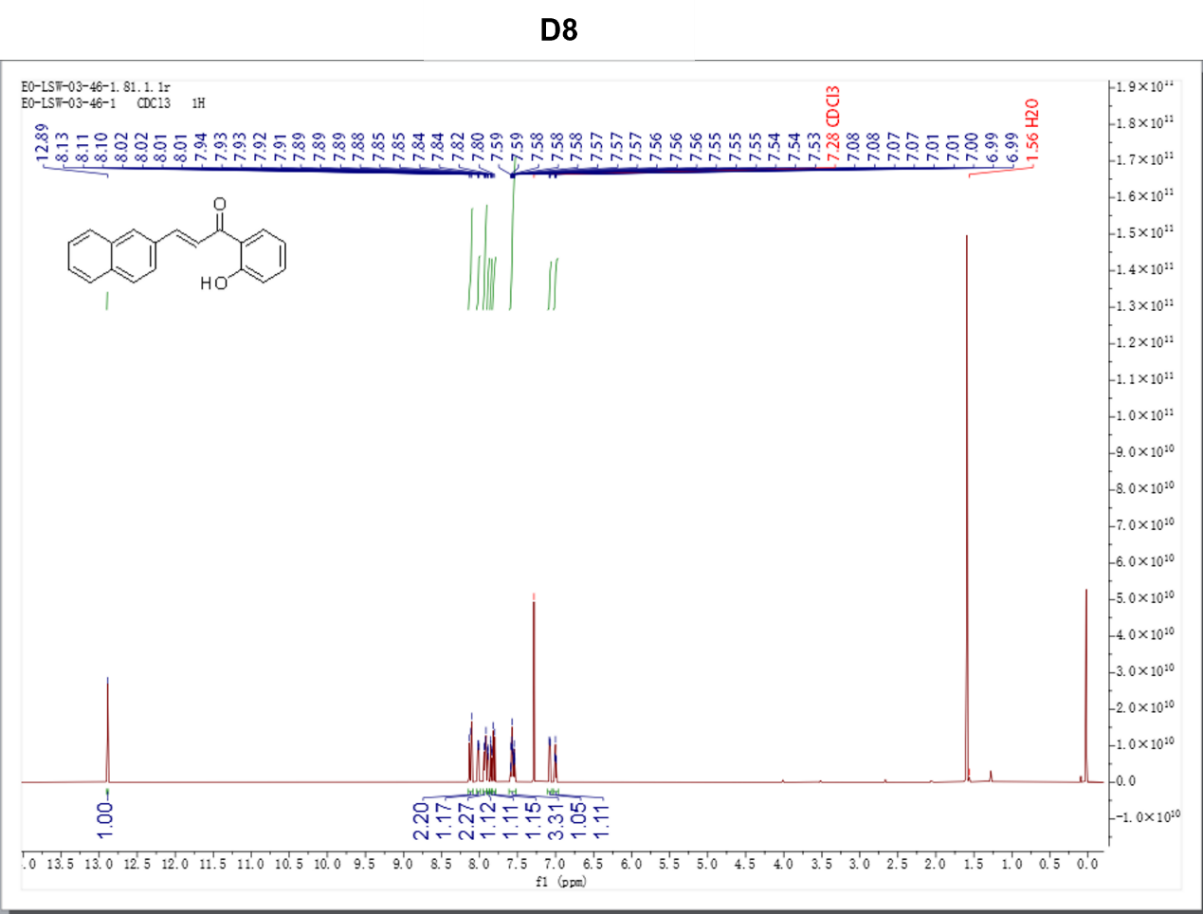


**Figure S59.** ^1^H NMR spectra of compound **D8**.


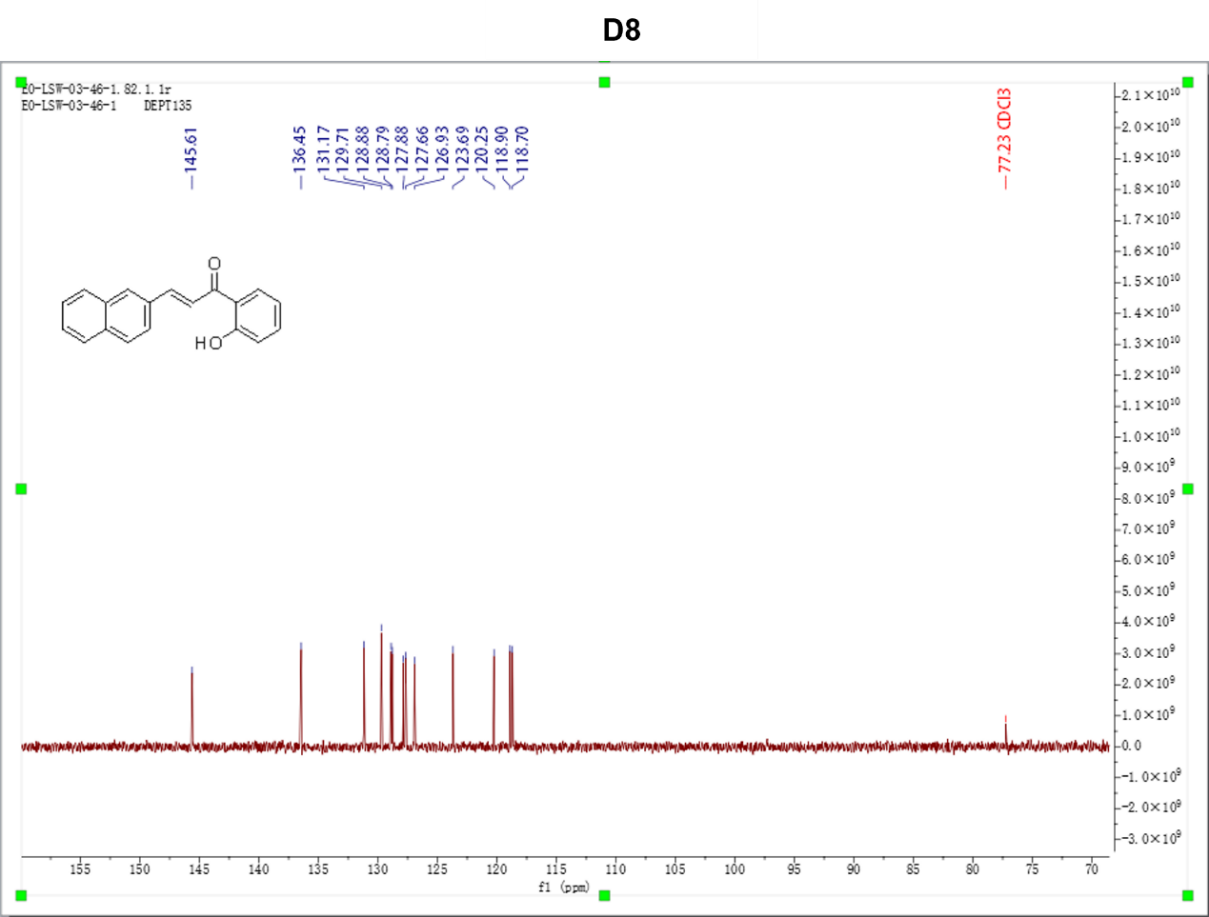


**Figure S60.** ^13^C NMR spectra of compound **D8**.


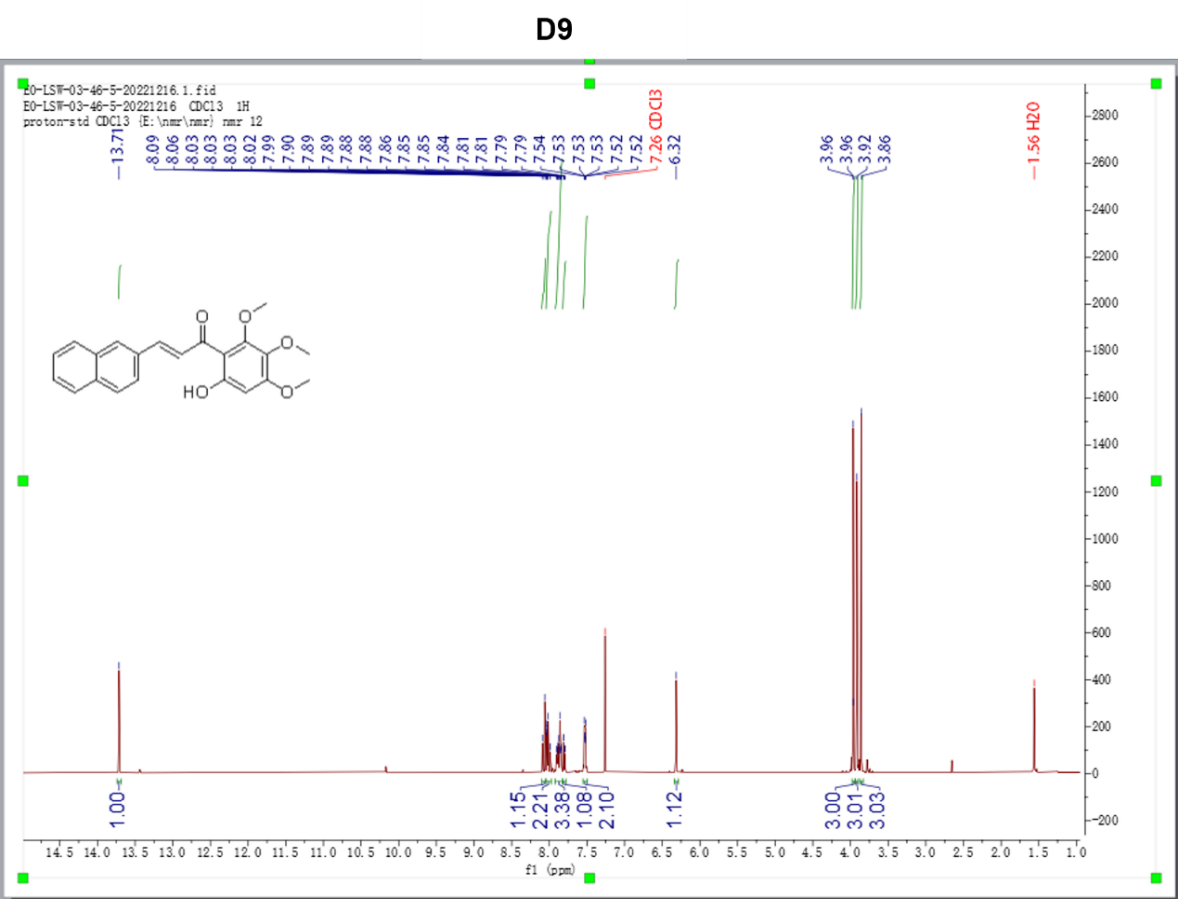


**Figure S61.** ^1^H NMR spectra of compound **D9**.


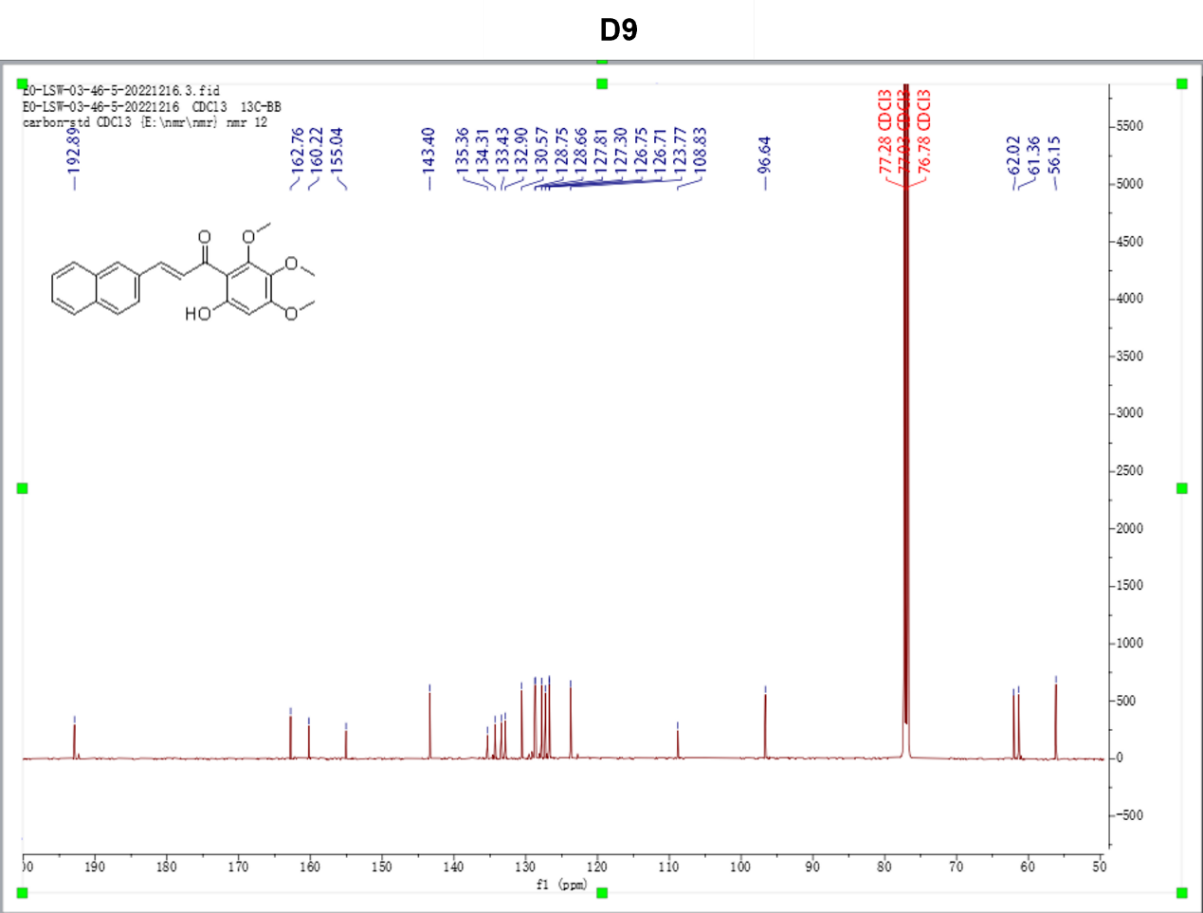


**Figure S62.** ^13^C NMR spectra of compound **D9**.


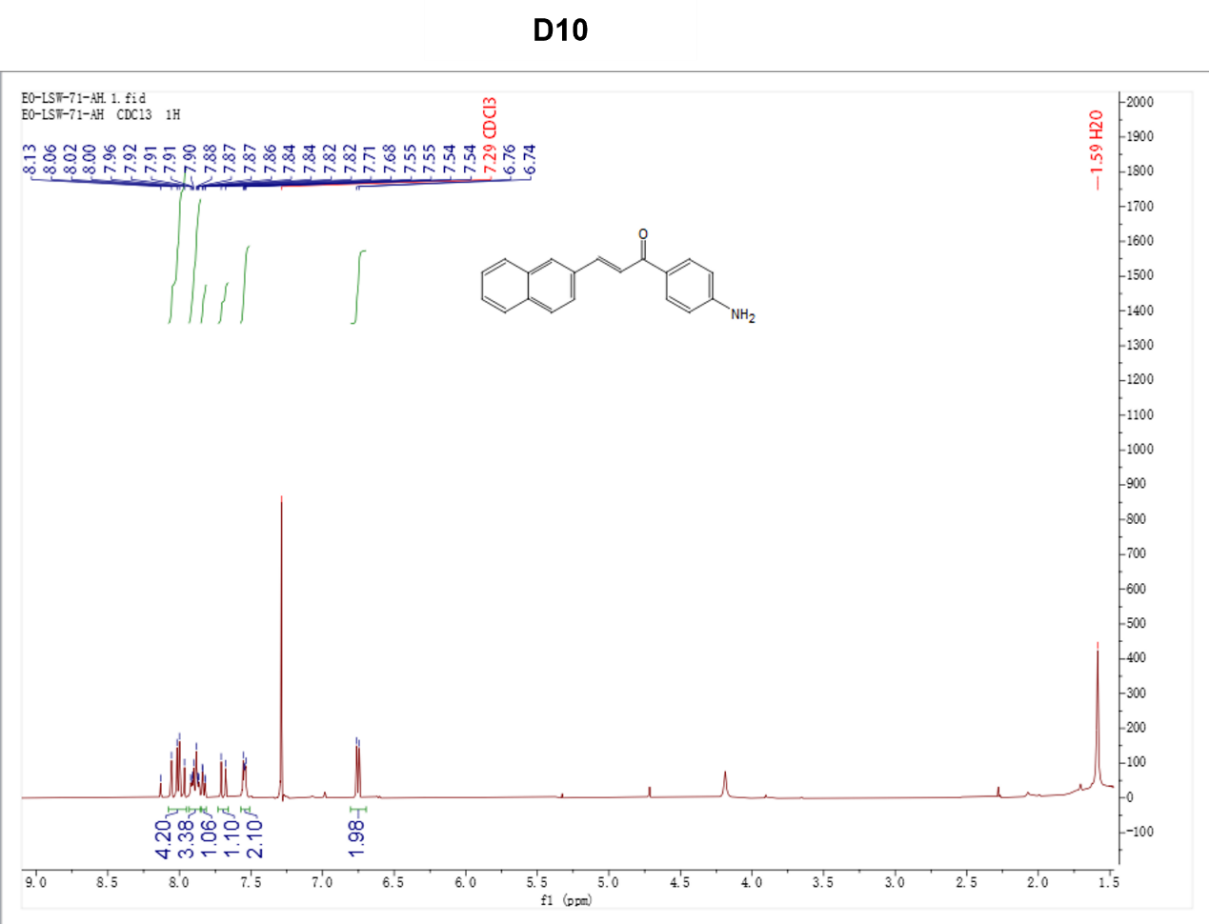


**Figure S63.** ^1^H NMR spectra of compound **D10**.


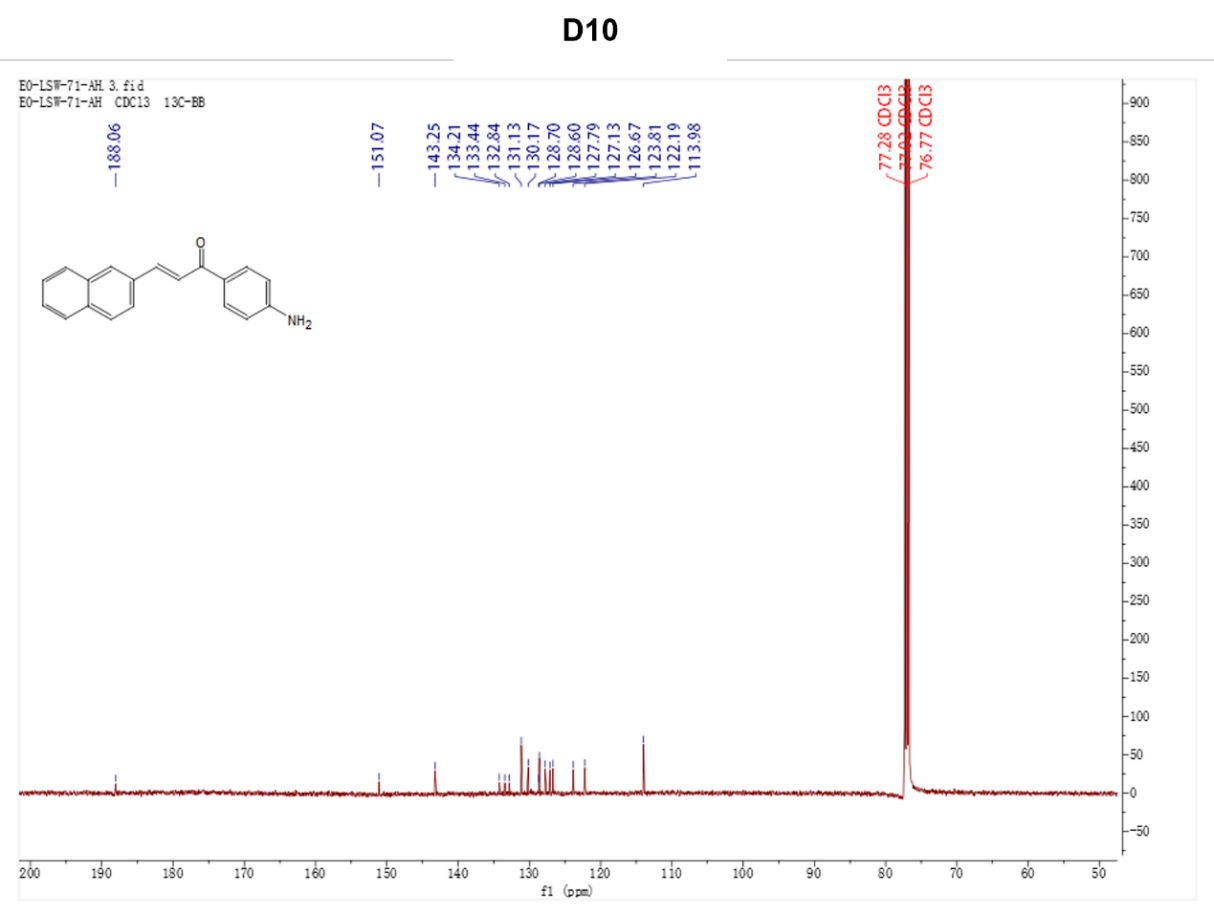


**Figure S64.** ^13^C NMR spectra of compound **D10**.


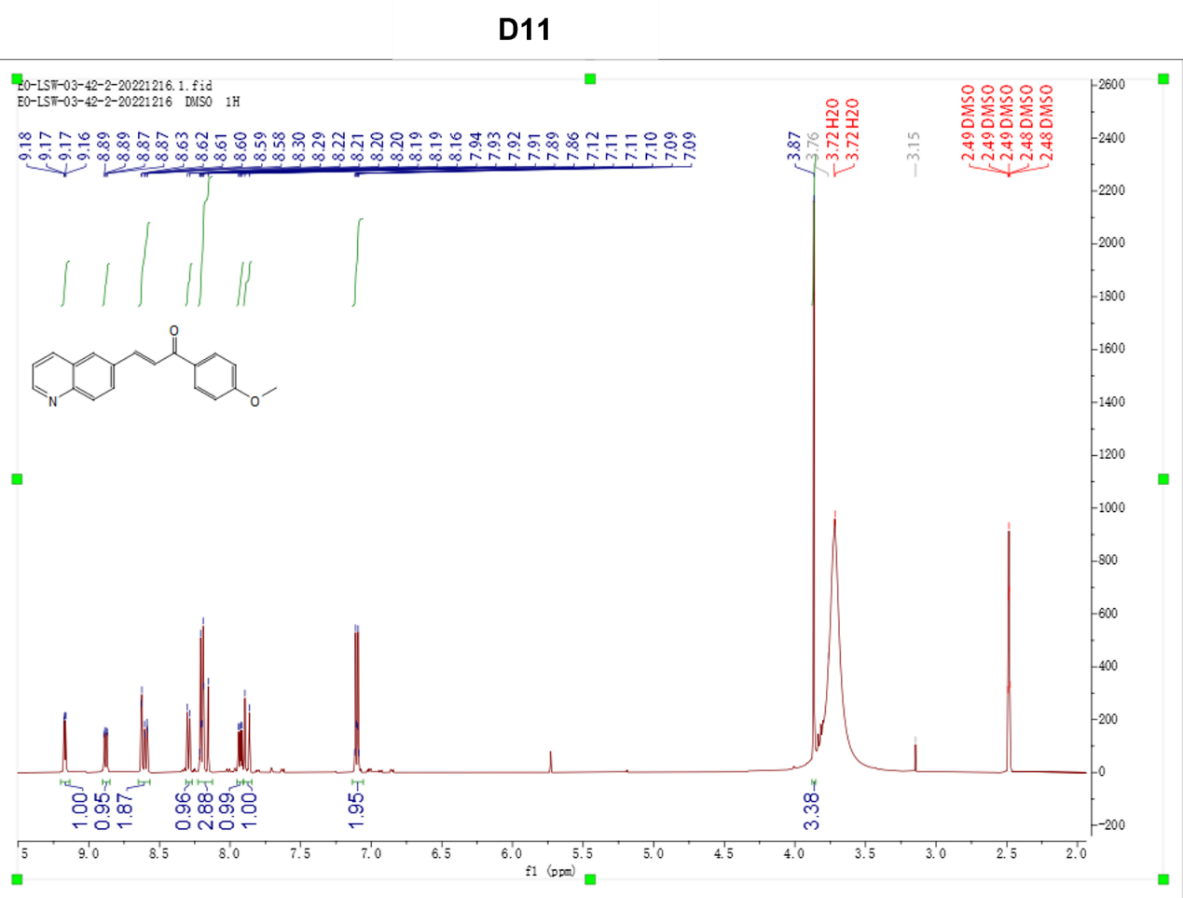


**Figure S65.** ^1^H NMR spectra of compound **D11**.


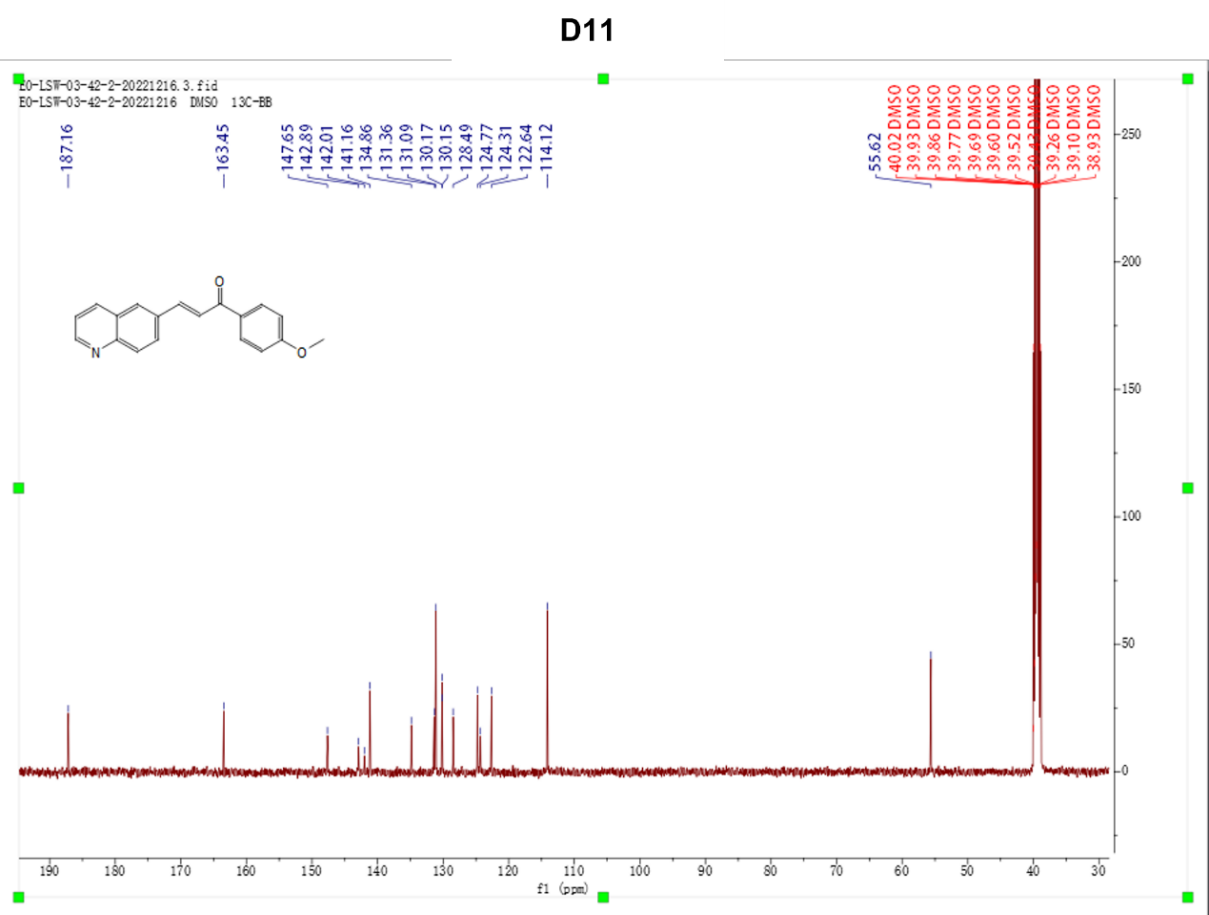


**Figure S66.** ^13^C NMR spectra of compound **D11**.


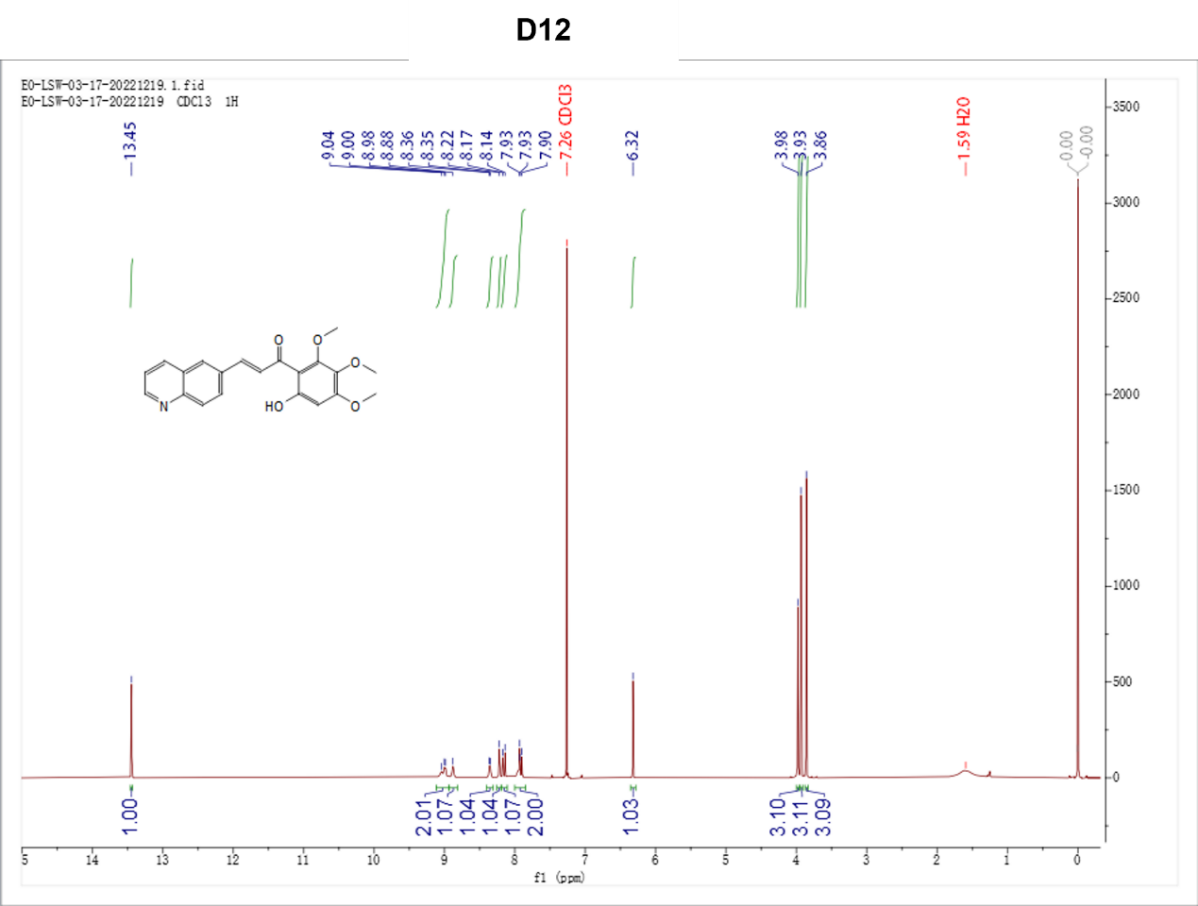


**Figure S67.** ^1^H NMR spectra of compound **D12**.


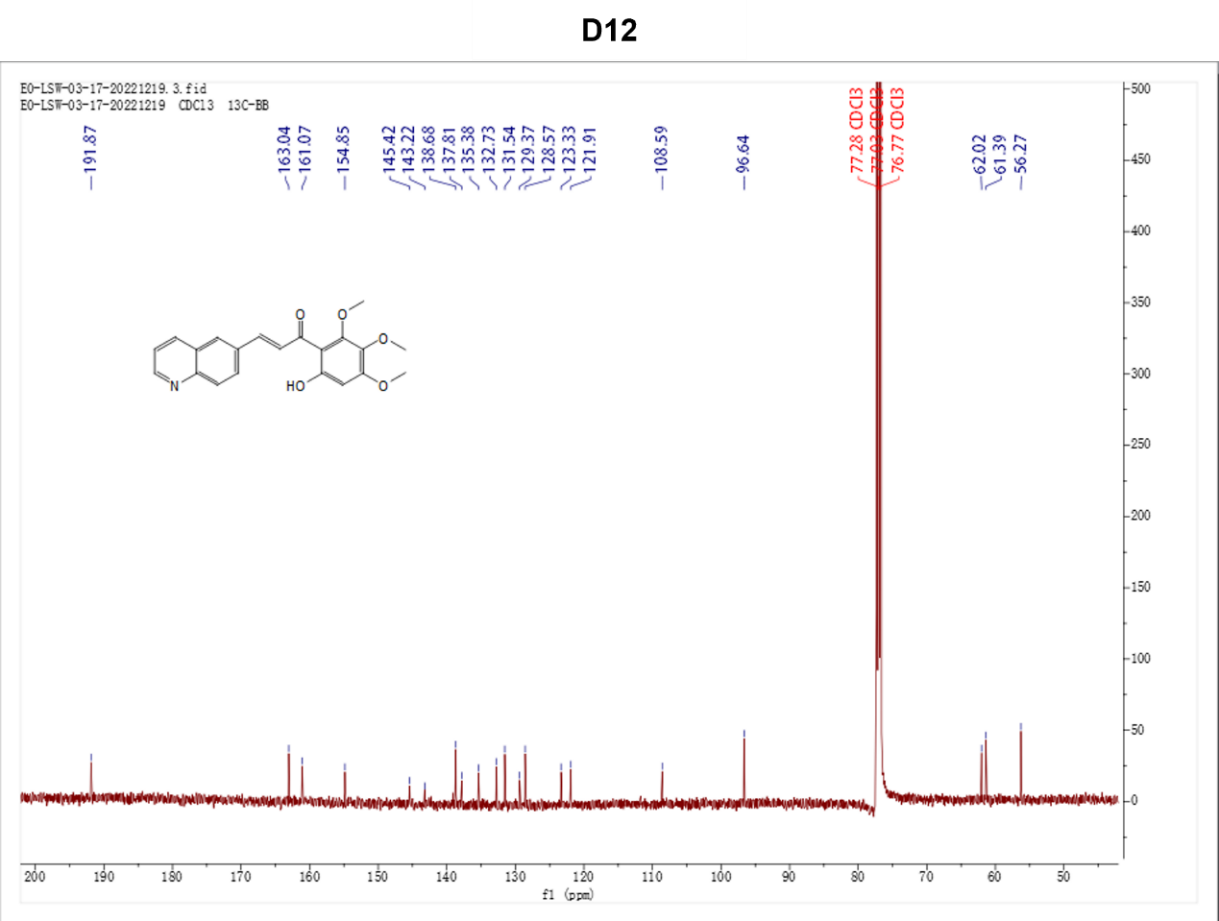


**Figure S68.** ^13^C NMR spectra of compound **D12**.


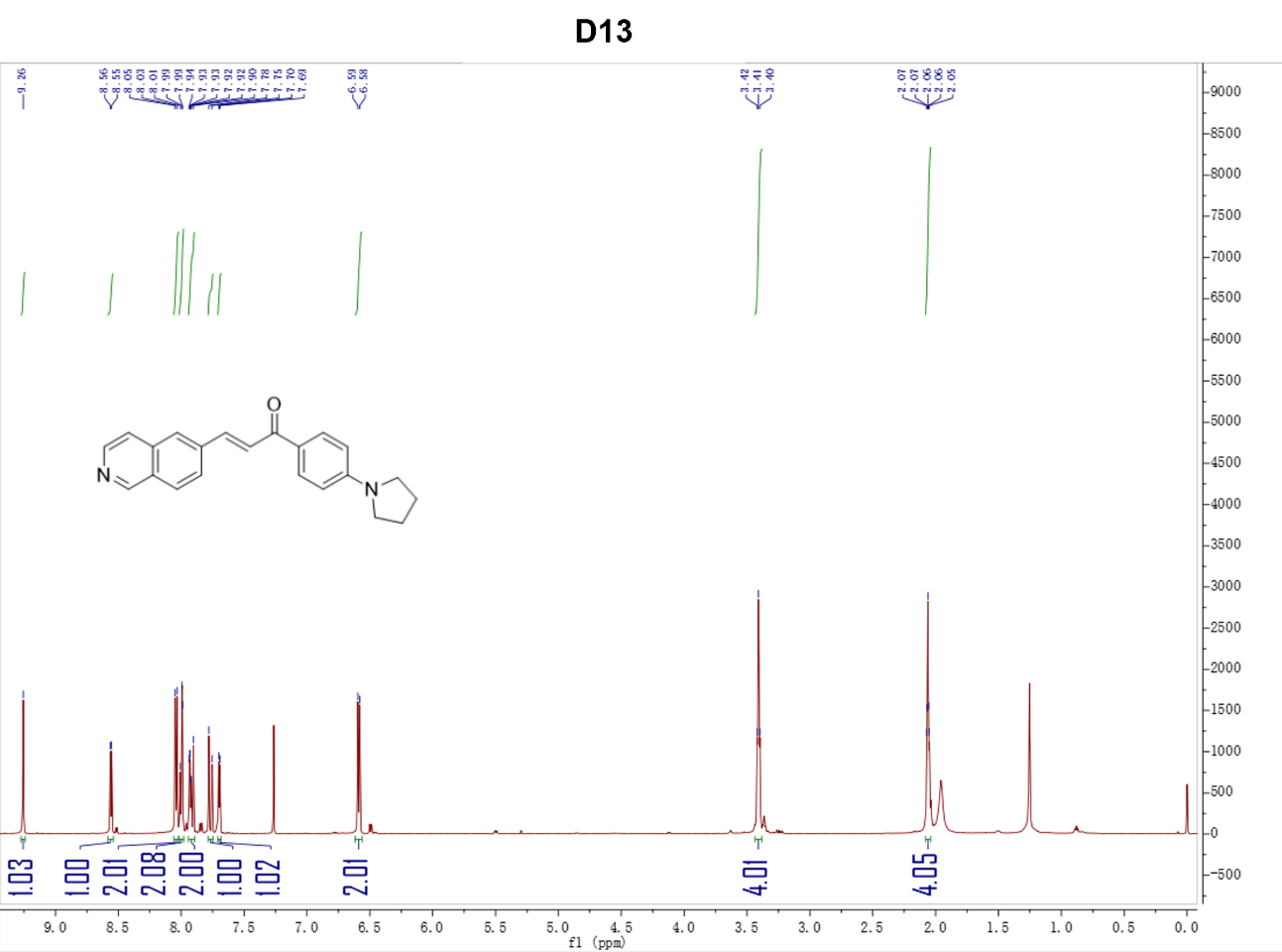


**Figure S69.** ^1^H NMR spectra of compound **D13**.


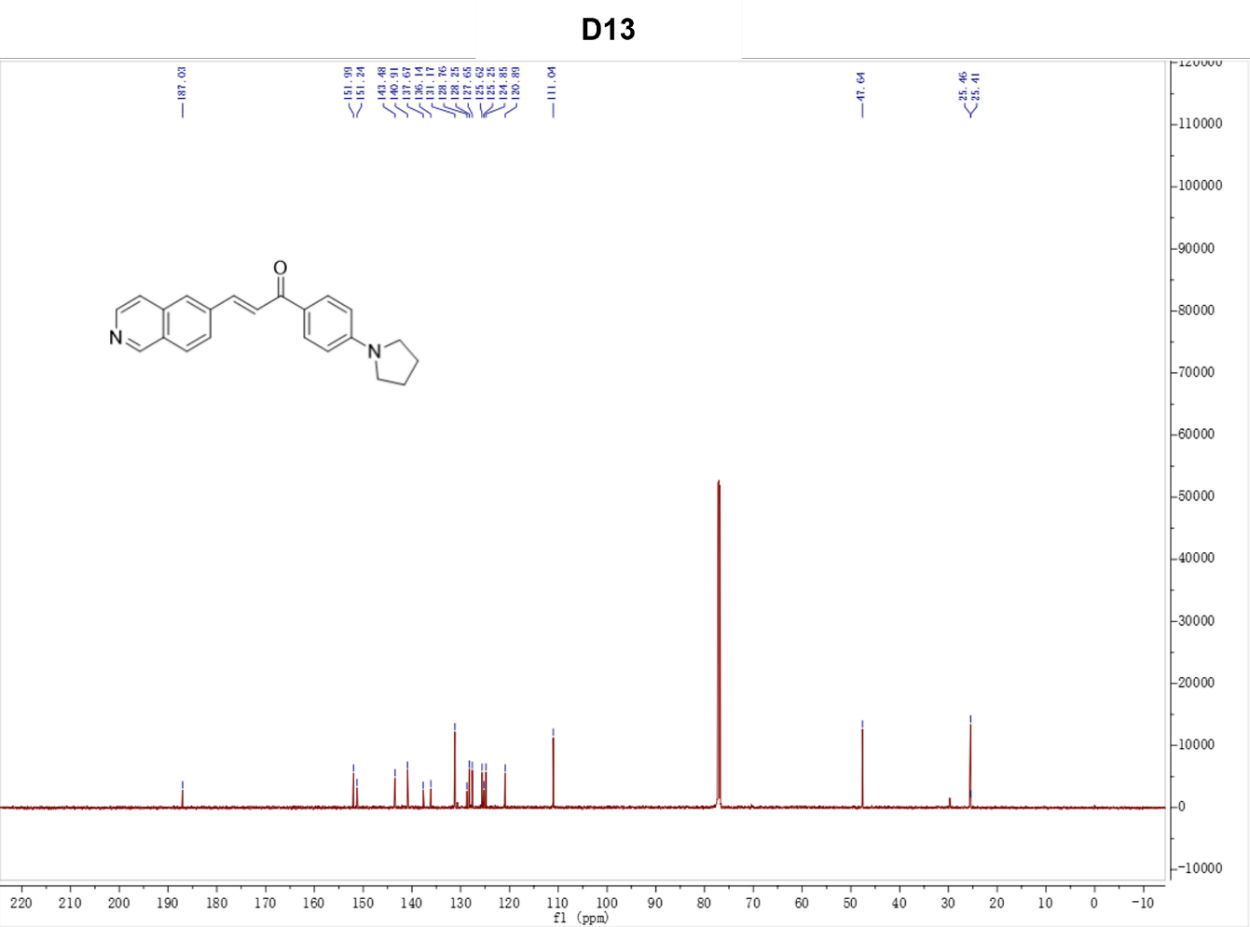


**Figure S70.** ^13^C NMR spectra of compound **D13**.


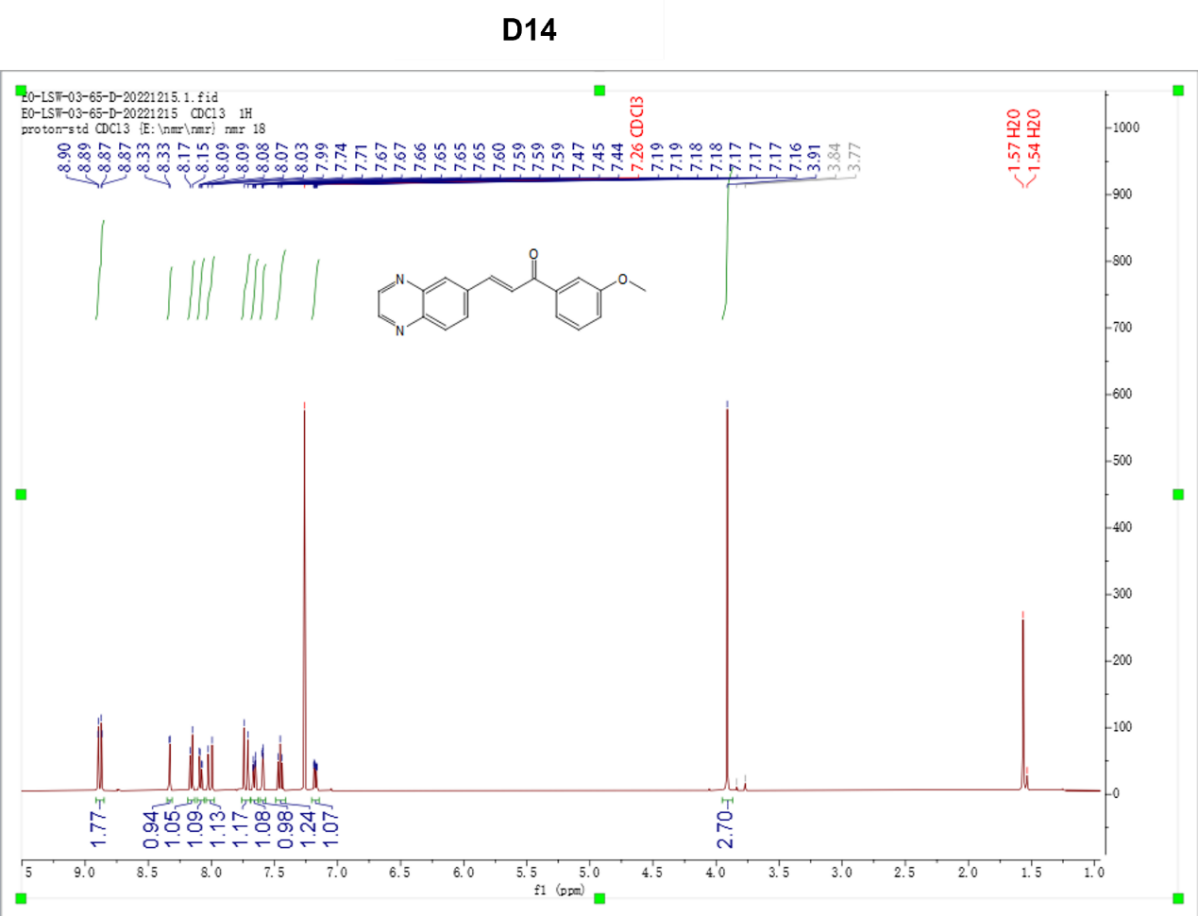


**Figure S71** ^1^H NMR spectra of compound **D14**.


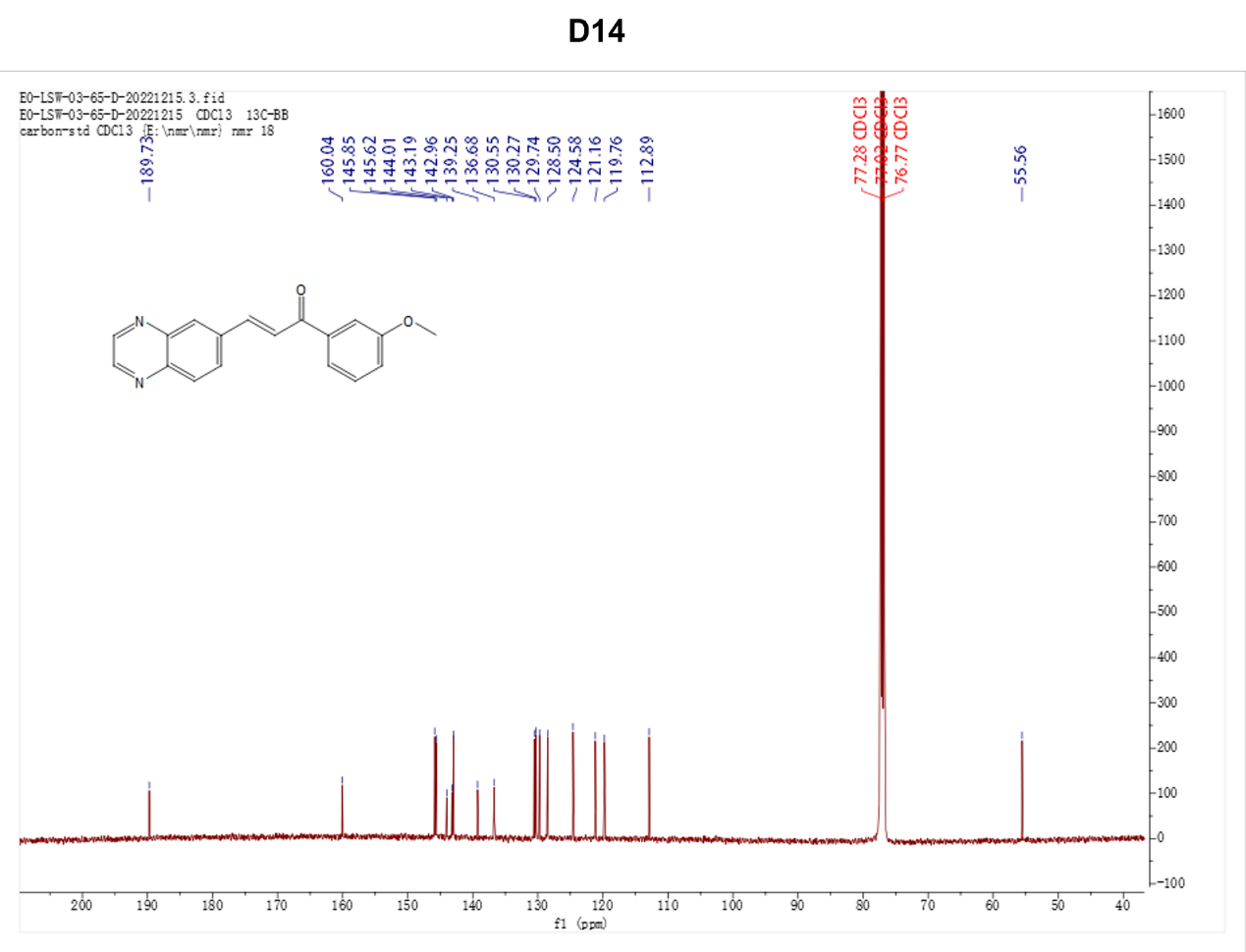


**Figure S72.** ^13^C NMR spectra of compound **D14**.


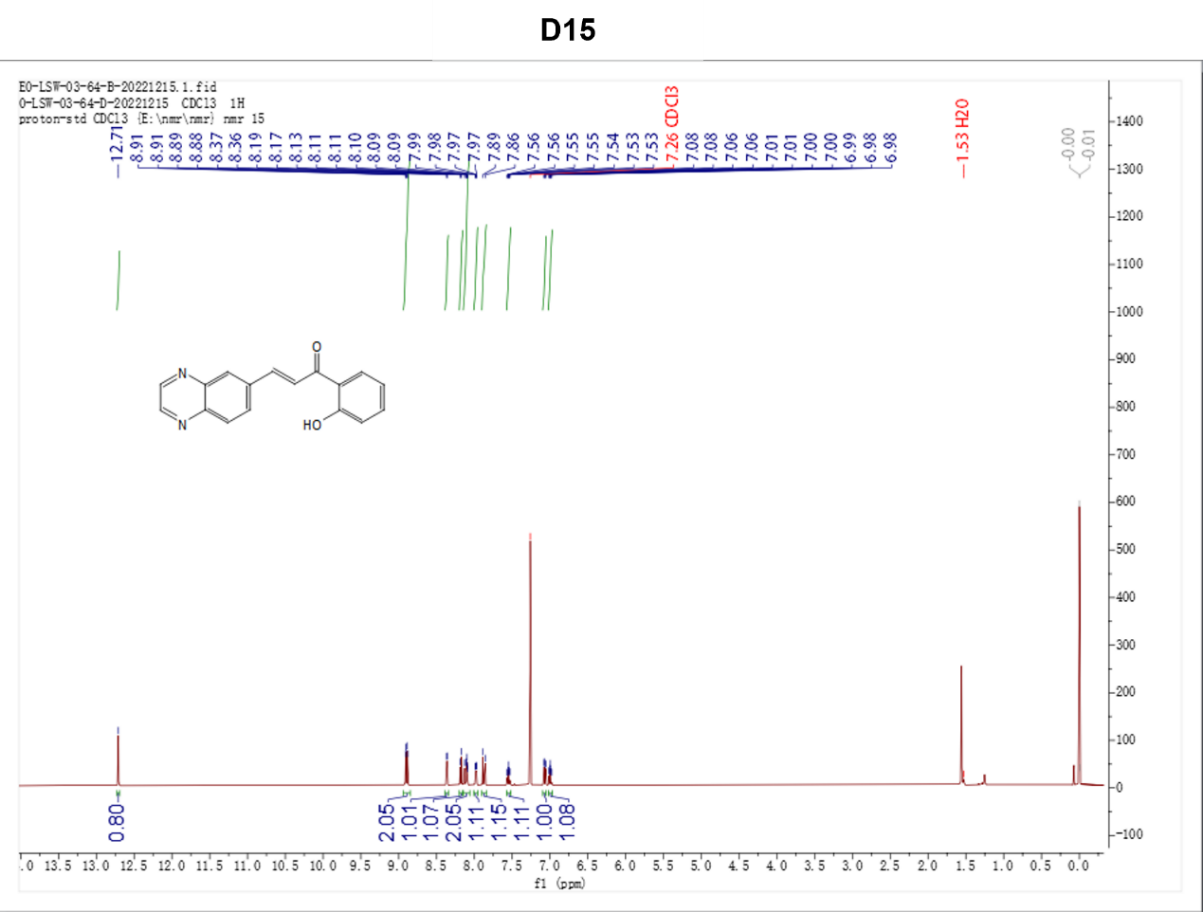


**Figure S73.** ^1^H NMR spectra of compound **D15**.


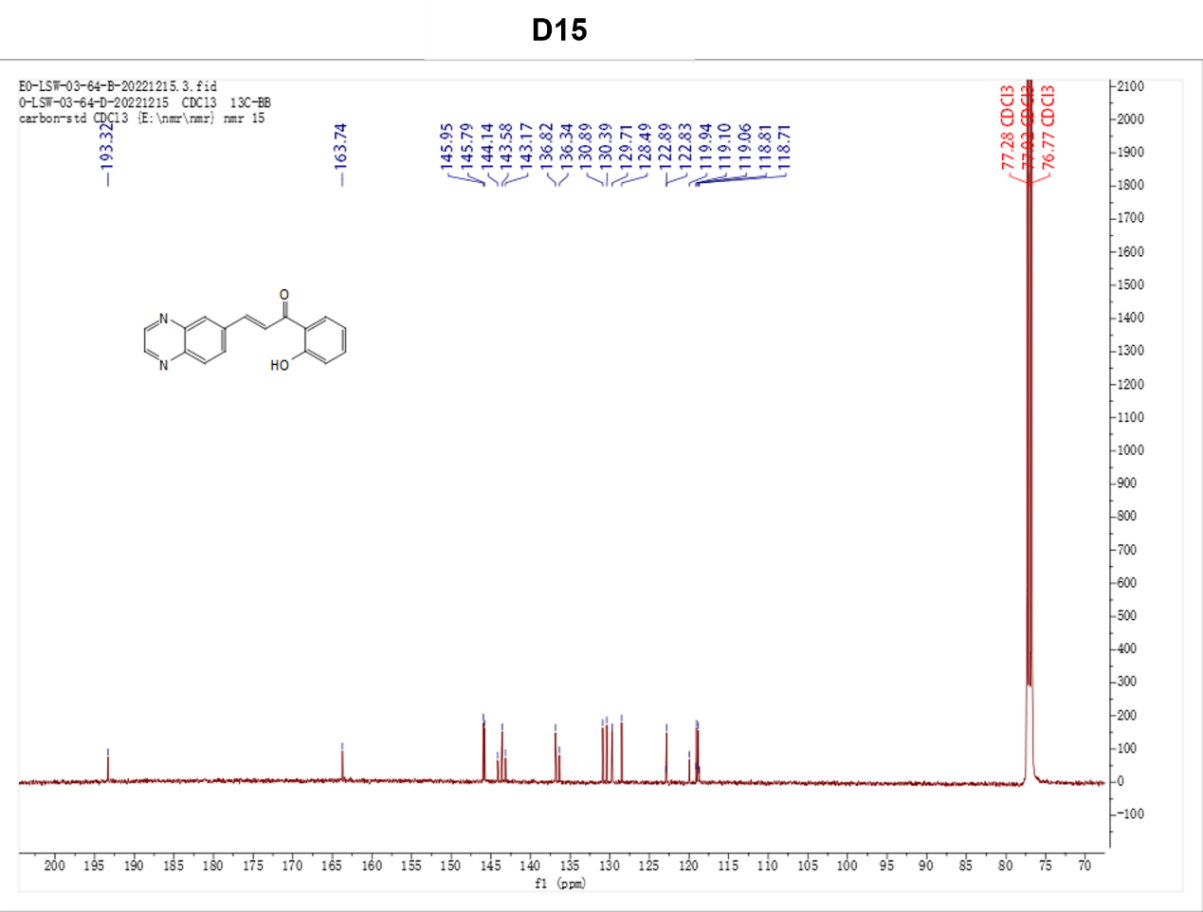


**Figure S74.** ^13^C NMR spectra of compound **D15**.


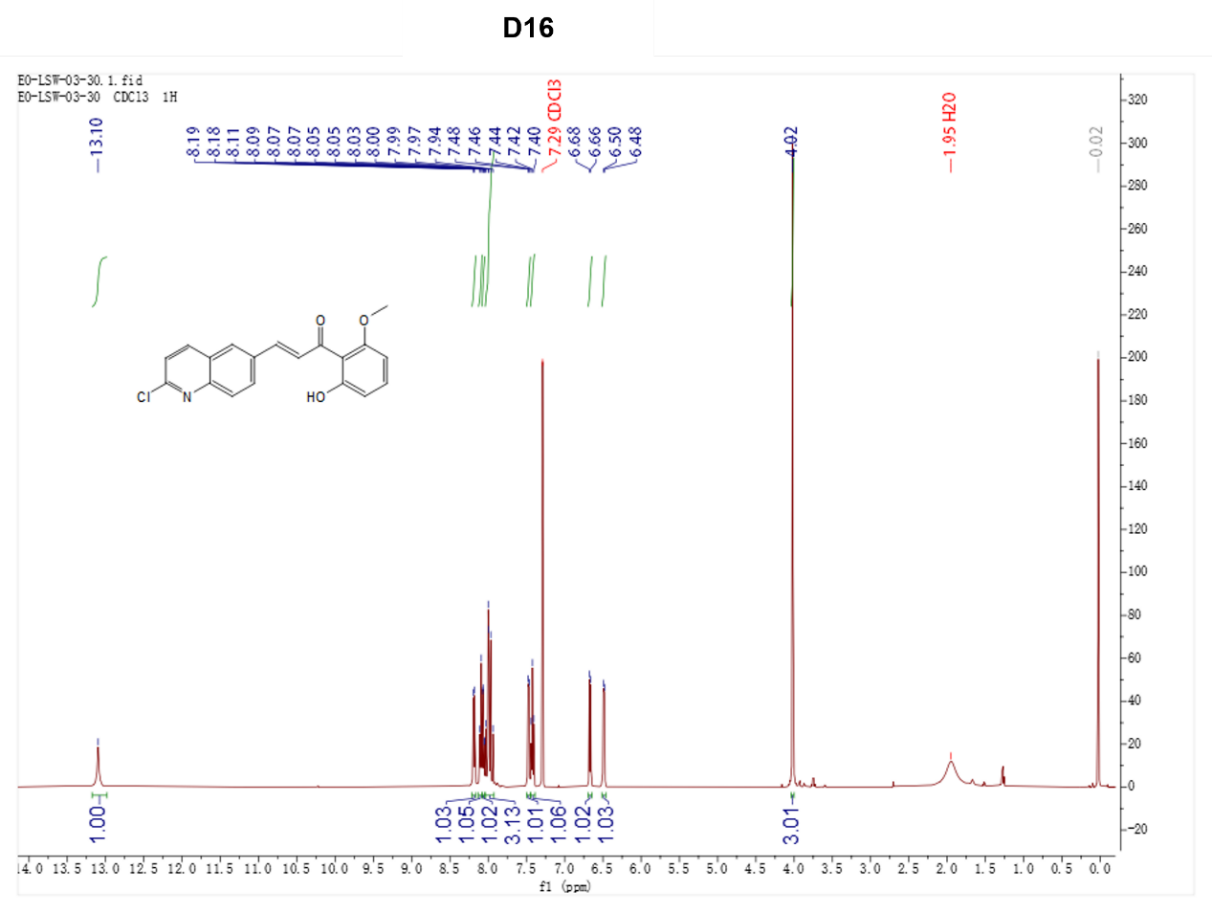


**Figure S75.** ^1^H NMR spectra of compound **D16**.


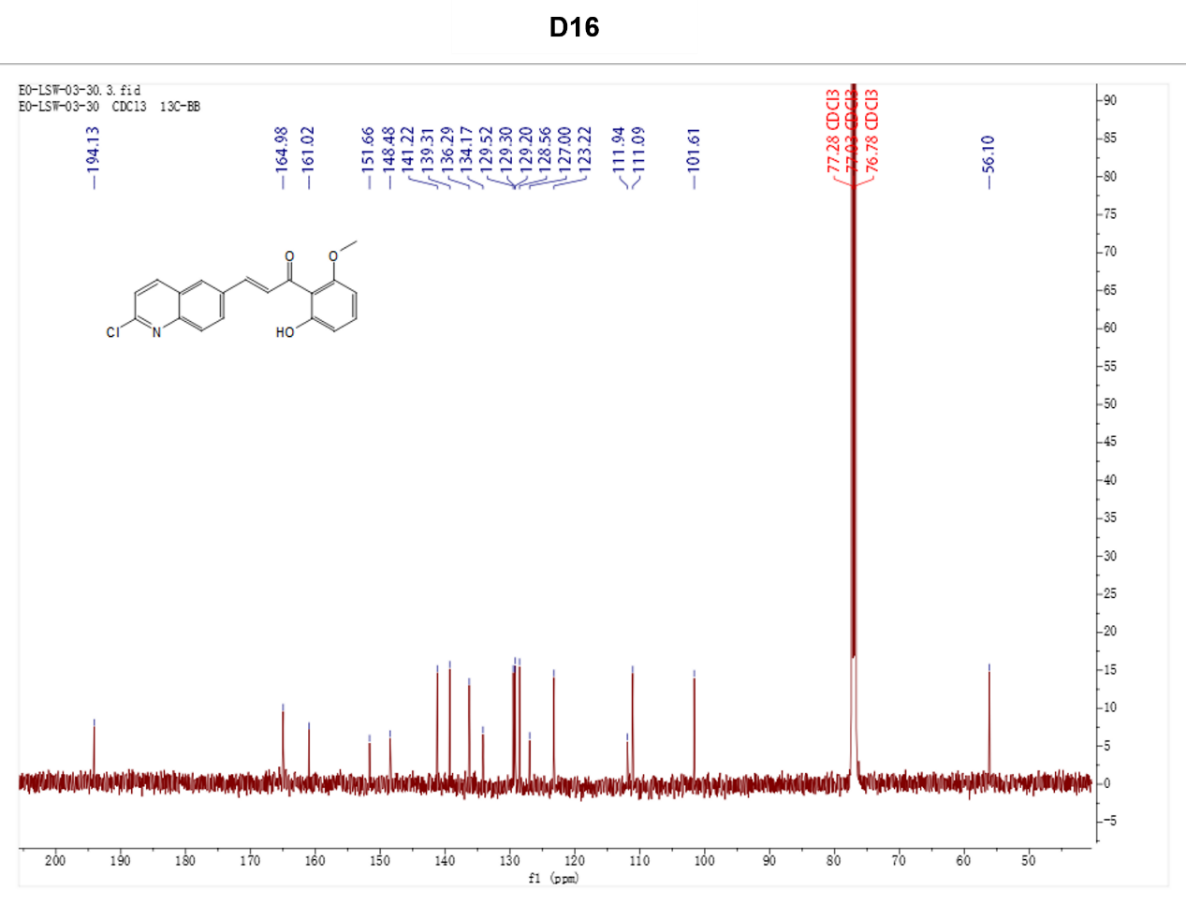


**Figure S76.** ^13^C NMR spectra of compound **D16**.


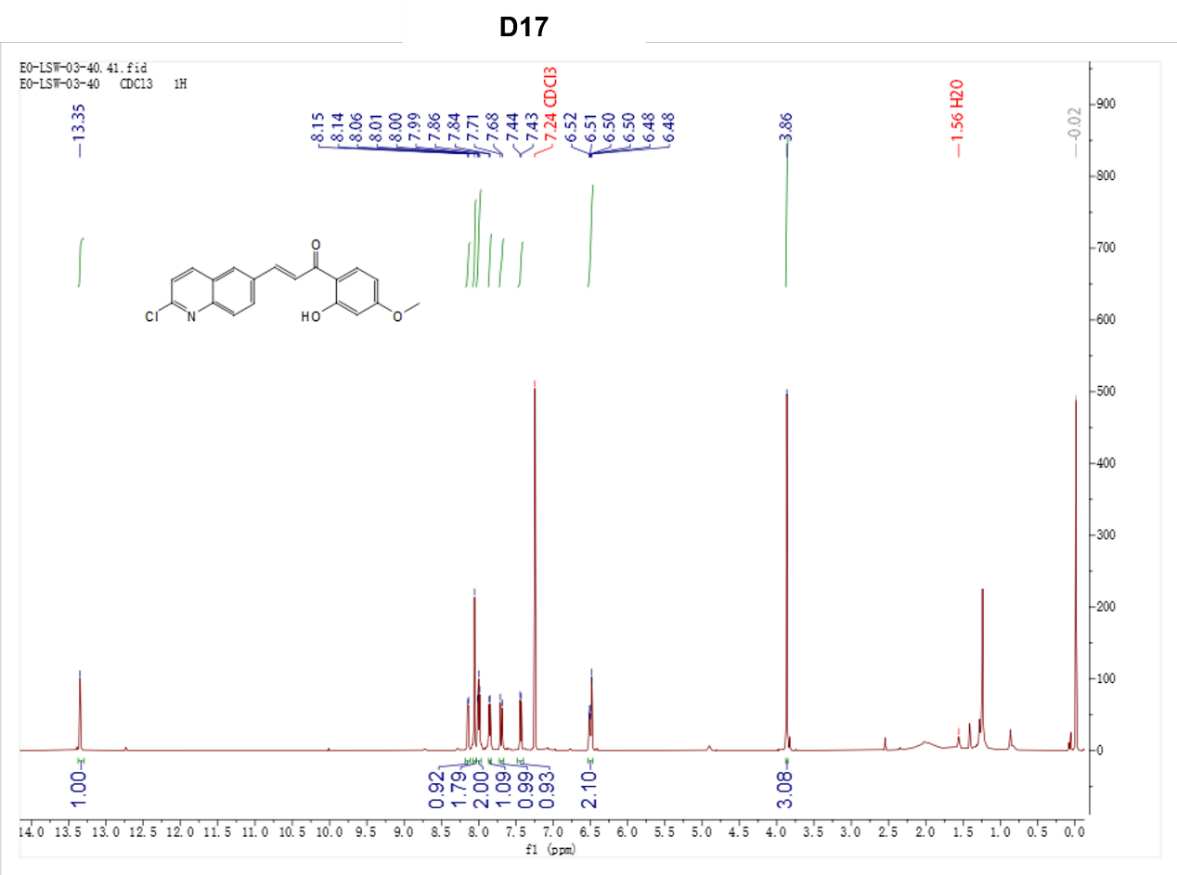


**Figure S77.** ^1^H NMR spectra of compound **D17**.


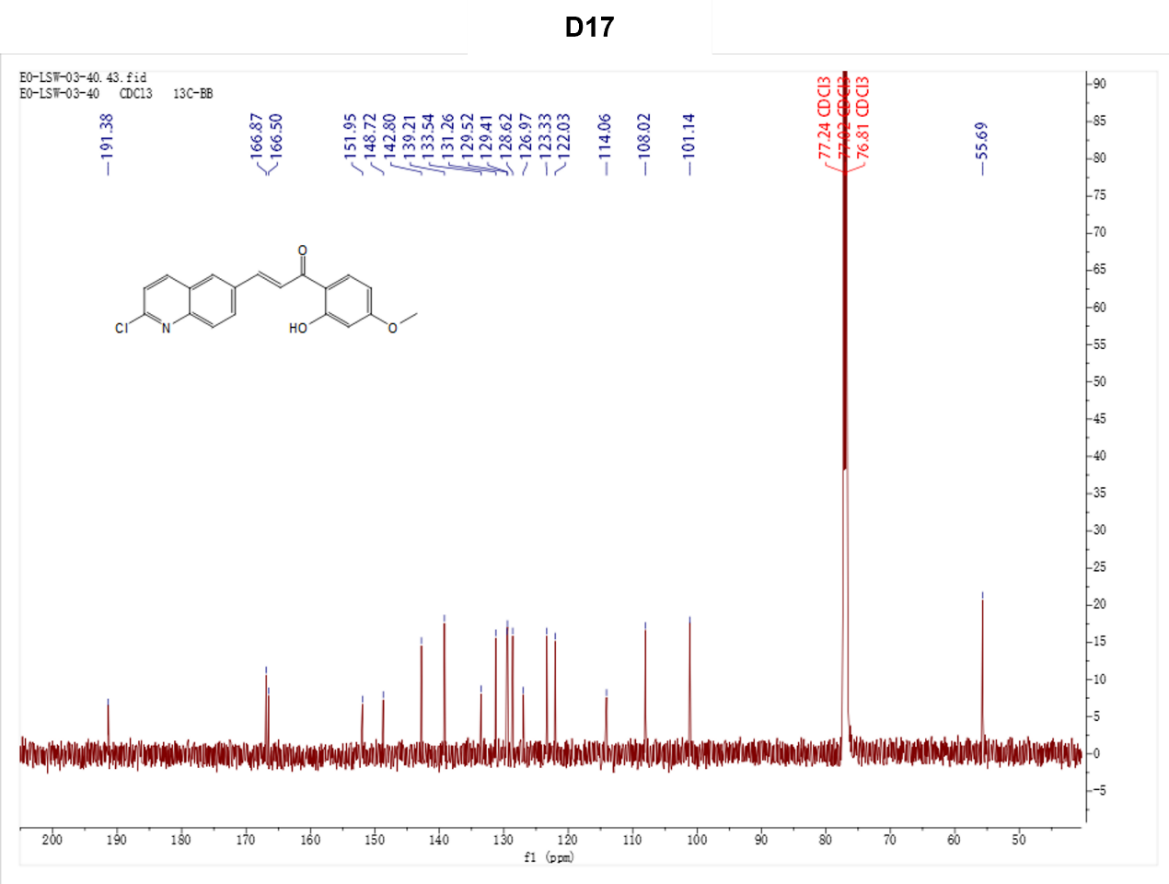


**Figure S78.** ^13^C NMR spectra of compound **D17**.


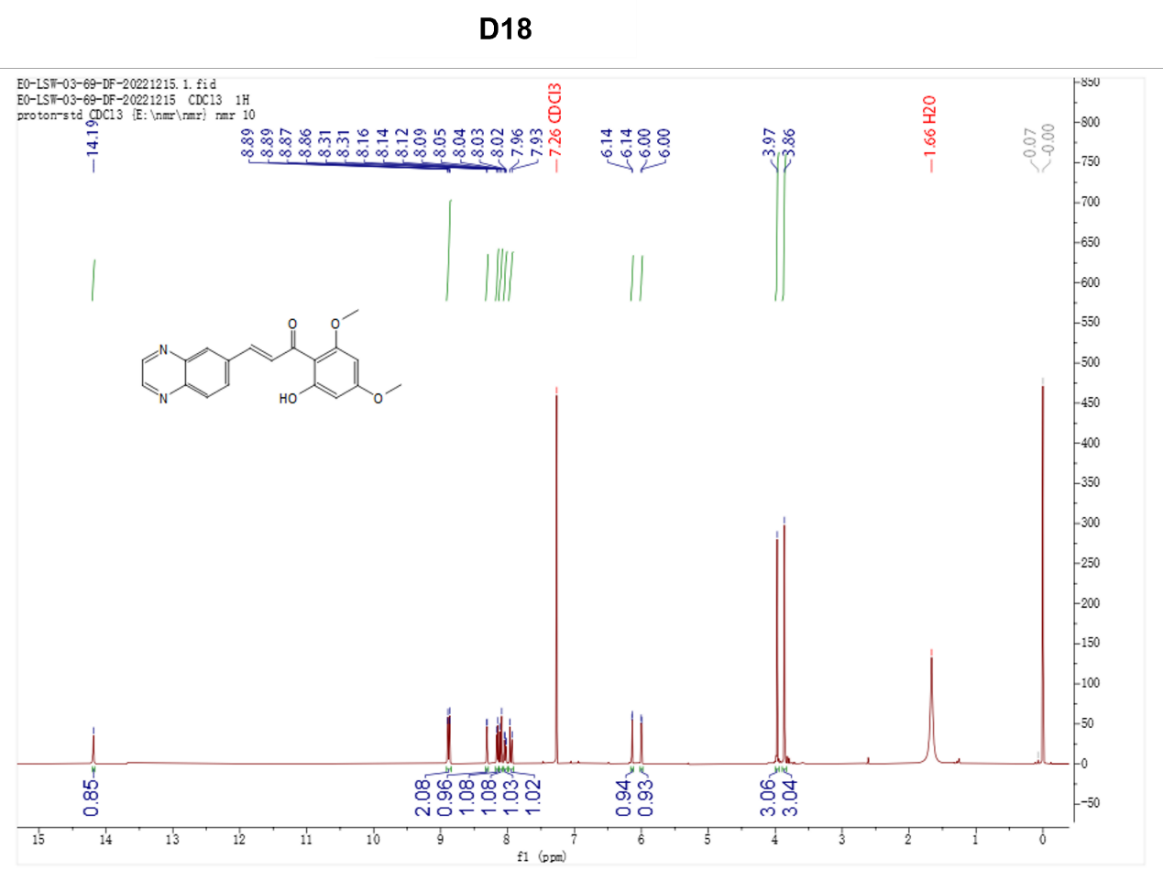


**Figure S79.** ^1^H NMR spectra of compound **D18**.


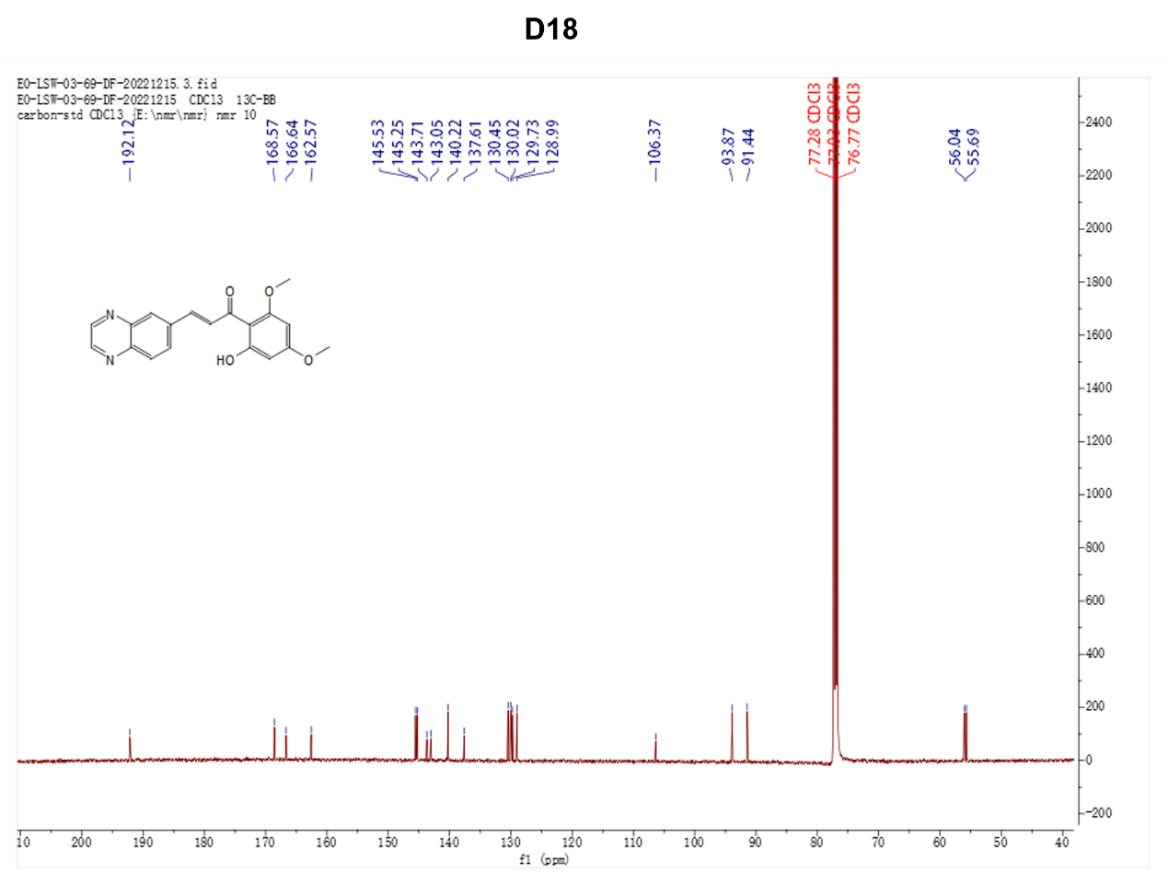


**Figure S80.** ^13^C NMR spectra of compound **D18**.
